# Whole-genome-based phylogenomic analysis of the Belgian 2016–2017 influenza A(H3N2) outbreak season allows improved surveillance

**DOI:** 10.1099/mgen.0.000643

**Published:** 2021-09-03

**Authors:** Laura A. E. Van Poelvoorde, Bert Bogaerts, Qiang Fu, Sigrid C. J. De Keersmaecker, Isabelle Thomas, Nina Van Goethem, Steven Van Gucht, Raf Winand, Xavier Saelens, Nancy Roosens, Kevin Vanneste

**Affiliations:** ^1^​ Transversal Activities in Applied Genomics, Sciensano, Juliette Wytsmanstraat 14, Brussels, Belgium; ^2^​ National Influenza Centre, Sciensano, Juliette Wytsmanstraat 14, Brussels, Belgium; ^3^​ Department of Biochemistry and Microbiology, Ghent University, Ghent, Belgium; ^4^​ VIB-UGent Center for Medical Biotechnology, VIB, Ghent, Belgium; ^5^​ Department of Plant Biotechnology and Bioinformatics, Ghent University, Ghent, Belgium; ^6^​ Department of Information Technology, IDLab, IMEC, Ghent University, Ghent, Belgium; ^7^​ Public Health and Genome, Sciensano, Brussels, Belgium

**Keywords:** beast, influenza, nextstrain, next-generation sequencing, surveillance

## Abstract

Seasonal influenza epidemics are associated with high mortality and morbidity in the human population. Influenza surveillance is critical for providing information to national influenza programmes and for making vaccine composition predictions. Vaccination prevents viral infections, but rapid influenza evolution results in emerging mutants that differ antigenically from vaccine strains. Current influenza surveillance relies on Sanger sequencing of the haemagglutinin (HA) gene. Its classification according to World Health Organization (WHO) and European Centre for Disease Prevention and Control (ECDC) guidelines is based on combining certain genotypic amino acid mutations and phylogenetic analysis. Next-generation sequencing technologies enable a shift to whole-genome sequencing (WGS) for influenza surveillance, but this requires laboratory workflow adaptations and advanced bioinformatics workflows. In this study, 253 influenza A(H3N2) positive clinical specimens from the 2016–2017 Belgian season underwent WGS using the Illumina MiSeq system. HA-based classification according to WHO/ECDC guidelines did not allow classification of all samples. A new approach, considering the whole genome, was investigated based on using powerful phylogenomic tools including beast and Nextstrain, which substantially improved phylogenetic classification. Moreover, Bayesian inference via beast facilitated reassortment detection by both manual inspection and computational methods, detecting intra-subtype reassortants at an estimated rate of 15 %. Real-time analysis (i.e. as an outbreak is ongoing) via Nextstrain allowed positioning of the Belgian isolates into the globally circulating context. Finally, integration of patient data with phylogenetic groups and reassortment status allowed detection of several associations that would have been missed when solely considering HA, such as hospitalized patients being more likely to be infected with A(H3N2) reassortants, and the possibility to link several phylogenetic groups to disease severity indicators could be relevant for epidemiological monitoring. Our study demonstrates that WGS offers multiple advantages for influenza monitoring in (inter)national influenza surveillance, and proposes an improved methodology. This allows leveraging all information contained in influenza genomes, and allows for more accurate genetic characterization and reassortment detection.

## Data Summary

All sequencing reads have been deposited in the National Center for Biotechnology Information (NCBI) Sequence Read Archive (SRA) under BioProject accession number PRJNA615341. All 253 generated consensus genome sequences have been deposited in the GISAID (Global Initiative on Sharing All Influenza Data) database (samples EPI_ISL_415199 to EPI_ISL_415452).

**Impact Statement**

Each year, seasonal influenza results in a high mortality and morbidity in humans. Vaccination still remains the best way to prevent viral infections and associated severe complications. The rapid influenza evolution gives rise to mutants that escape immunity and antigenically differ from vaccine strains. Worldwide virological surveillance during each epidemic allows predictions and recommendations to be made for the ideal vaccine composition for the next influenza season. Although the haemagglutinin (HA) segments remain the principal regions for Sanger sequencing in classical influenza surveillance programmes, we illustrated the feasibility and benefit of switching towards whole-genome-sequencing-based surveillance. This study proposed an improved method for classification of circulating strains with higher resolution for genetic characterization and reassortment detection. This also allows a better insight into viral spread and improved detection of transmission clusters that would not have been possible when solely sequencing the HA segments. Additionally, whole-genome information from all eight segments will not only improve current vaccine strain selection, but could potentially become a requirement in the future as next-generation vaccines and antiviral drugs start appearing that do not focus solely on the HA and NA (neuraminidase) segments. Lastly, integration of whole-genome data with patient information also allows investigation of associations of genetic groups based on the whole genome with host characteristics.

## Introduction

Every year, 5–20 % of the human population becomes infected with influenza. Worldwide, 3 to 5 million infections yearly progress into severe cases [1]. Case-fatality rates are <0.1 % during a typical influenza pandemic [2]. Severe cases predominate in certain risk groups, including the very young and very old, and patients with comorbidities such as chronic cardiac, respiratory and metabolic diseases, obese patients, immunocompromised patients and pregnant women [3]. Influenza A and B viruses are a major cause of respiratory tract infections in humans. The influenza A genome consists of eight segments, including two segments encoding haemagglutinin (HA) and neuraminidase (NA) proteins. HA and NA are considered the most important viral components because they represent key antigens due to their location on the viral envelope, rendering them the main immune response targets [4–6]. Influenza A viruses are further classified into subtypes based on the combination of their HA and NA segments. Currently, influenza A(H1N1)pdm09 and A(H3N2) are the two main influenza A subtypes circulating in humans [1].

Influenza surveillance is important to determine the vaccine composition based on circulating influenza virus strains, and to provide information to national influenza prevention and control programmes regarding the timing, impact and severity of seasonal epidemics. Additionally, surveillance allows the detection of emerging zoonotic and potentially pandemic influenza viruses [7]. According to the guidelines of the World Health Organization (WHO) and the European Centre for Disease Prevention and Control (ECDC), influenza surveillance requires classification of new samples into different clades and subclades within each influenza subtype. This surveillance is based on the combination of certain predefined genotypic amino acid variants present in the HA segment, and phylogenetic analysis for which the HA segment of samples should cluster within clades represented by indicated vaccine or reference virus strains. Additionally, the HA gene should exhibit neither many (although an exact threshold is not defined) nor critical (i.e. those that significantly affect antigenicity) amino acid differences compared to the indicated vaccine or reference strain with which they associate [8]. Based on circulating strains identified in surveillance programmes, respective HA-based clade classification, and availability of vaccine viruses, the vaccine composition for the following season is determined.

Consequently, the main focus of genetic surveillance is the HA gene, with proportionally limited data available for the other seven segments [9]. Sanger sequencing currently remains the principal approach for genetic influenza surveillance. However, next-generation sequencing (NGS) technologies are increasingly used in many countries [10–13] and constitute a promising alternative, offering the possibility to simultaneously obtain the sequence of all eight segments. Whole-genome sequences can provide much additional information for influenza surveillance compared to solely sequencing the HA gene. These whole-genome sequences can assist in inferring potential links between genomic data and host characteristics, including epidemiological effect exploration of inter- and intra-seasonal evolutionary dynamics, inter- and intra-subtype reassortment detection, identification of mutations located anywhere in the genome [14–16], and genetic group strain classification based on whole-genome information. Additionally, whole-genome sequences can improve vaccine strain selection and enhance vaccine efficacy [17]. Lastly, multiple next-generation vaccines target other segments than the HA segment [18], requiring new approaches for influenza monitoring based on the whole genome. Most of these sequences are deposited in the database maintained by the Global Initiative on Sharing All Influenza Data (GISAID), which contains genome sequences of all influenza types and includes outbreaks and surveillance studies [19].

Since the 1968 influenza A(H3N2) pandemic, the A(H3N2) subtype has led to numerous seasonal epidemics and is considered to evolve faster than other subtypes [20]. A(H3N2) has shown extensive genetic diversity and increased morbidity and mortality in recent years, especially in the elderly [21]. Identifying and predicting current epidemiological threats from A(H3N2) is challenging due to the strain’s rapid evolution and the current limitations of existing methods for analysing the antigenic characteristics of influenza A(H3N2) [22]. This rapid evolution is caused by mutations but also reassortments. Reassortments can occur due to the segmented genome when cells are infected with different influenza viruses, when new viral particles are assembled with a mix of segments from these different viruses [23]. This rapid evolution is reflected by the continuous updates of WHO recommendations regarding vaccine strains for influenza A(H3N2) [24]. Sporadically, inter-subtype reassortment occurs, which may give rise to viruses with pandemic potential. Two recent examples of inter-subtype reassortment include a case in the Netherlands in March 2018 and a case in Sweden in January 2019, both of which resulted in influenza subtype A(H1N2) [25]. Nevertheless, inter-subtype reassortments are rare due to potential segment incompatibility between heterologous viral components resulting in RNA or protein mismatches that decrease viral fitness and limit dispersion in the human population [26]. In contrast, intra-subtype reassortments between various lineages of the same subtype happen more frequently because of the higher genetic relatedness and functional compatibility of their segments. Intra-subtype reassortments can increase the adaptive potential and genetic diversity of circulating viruses [15]. For the A(H3N2) subtype, it was suggested that the rate of adaptive amino acid replacements within reassorted strains is temporarily increased. Although intra-subtype reassortments have not been systematically evaluated, they had a major impact on virus evolution [15].

As the role of intra-subtype reassortment is becoming increasingly clear, identifying and monitoring reassortments with whole-genome-based surveillance becomes necessary [27, 28]. However, implementing this whole-genome-based surveillance in routine surveillance requires multiple adaptations in the laboratory workflow. Additionally, in-depth bioinformatics expertise is required to process sequencing results. Hence, the availability of user-friendly tools and pipelines is paramount for the incorporation of whole-genome sequencing (WGS) into routine surveillance [29]. In this study, the feasibility of WGS is evaluated for routine influenza surveillance based on the WGS of 253 A(H3N2) samples from the 2016–2017 Belgian influenza season [30]. In particular, the suitability of current methods for defining phylogenetic groups based on the HA segment versus their whole genome is evaluated to assess the added value of incorporating whole-genome information for interpretation of strain clusters and their reassortment status. Several high-end computational methods are explored to improve classification and detection of reassortments, and this study proposes a new methodology based on WGS for genetic influenza surveillance.

## Methods

### Sample selection, RNA isolation, PCR amplification and WGS

#### Sample selection

Two main surveillance systems exist in Belgium, ‘influenza-like-illness’ (ILI) and ‘severe-acute-respiratory-infection’ (SARI). A sudden onset of symptoms, including fever and respiratory and systemic symptoms, define ILI cases. A SARI case is an acute respiratory illness requiring hospitalization with fever and respiratory symptoms onset within the previous 10 days. A standard questionnaire accompanied all samples with patient information on sex, birth date, clinical features, vaccination status, administration of antiviral treatment or antibiotics, date of symptom onset and date of sample collection (Supplementary Methods).

From these two surveillance systems, 253 samples were selected with a *C*_q_ <32 as detected with quantitative reverse transcription PCR (RT-qPCR). Samples were mainly selected by stratifying based on the severity, patient age and sampling date. Samples from outpatients (ILI) were all categorized as mild cases (*n*=93), whereas samples from hospitalized patients (SARI) were categorized as either moderate (*n*=122) or severe (*n*=38) cases. Patient age and sampling dates were stratified into three groups: patients <15 years, patients between 15 and 59 years, and patients ≥60 years. Samples were categorized based on their sampling history: before, during (week 4 to 6 2017) and after the epidemic peak. An overview of available host characteristics for all 253 samples is provided in Table 1.

**Table 1.** Samples stratified according to host characteristics

Epidemic stage	No. of samples		
		Age (Years)	
	<15	15–59	≥60
Beginning of epidemic (< week 4)	12	17	35
Peak of epidemic (week 4–6)	16	26	86
End of epidemic (> week 6)	11	16	34
**Characteristic**	**No.**	**Characteristic**	**No.**
ILI	93	SARI	160
Male	122	Female	122
Vaccinated	52	Not vaccinated	130
Antibiotics administered	100	No antibiotics administered	126
Respiratory disease	50	No respiratory disease	199
Cardiac disease	54	No cardiac disease*	195
Obesity	20	No obesity	233
Renal insufficiency	35	No renal insufficiency	218
Hepatic insufficiency	6	No hepatic insufficiency	247
Diabetes	27	No Diabetes	226
Immuno-deficiency	23	No immuno-deficiency	230
Neuromuscular disease	21	No neuromuscular disease	232
Stay in ICU	22	No stay in ICU	231
Resulting in death	19	Not resulting in death	234

*Samples for which certain host characteristics were unknown, were excluded for analysing that particular host characteristic.

#### RNA isolation, PCR amplification and WGS

Nucleic acids of samples were extracted directly from the clinical specimens [31] using a viral RNA/DNA isolation kit (Macherey Nagel). RNA extraction was performed according to the manufacturer’s instructions, except that beads were not washed in buffer MV5 but instead dried for at least 10 min or longer, until the pellet did not appear shiny anymore, before continuing.

Sequencing amplicons were generated in a one-step reverse transcription PCR (RT-PCR), in a 50 µl reaction volume with three primers allowing reverse transcription and amplification of each segment. This protocol is based on that of Van den Hoecke *et al*. [32] with optimized volumes and RT-PCR conditions (Supplementary Methods).

Amplified products were purified using a NucleoSpin Gel and PCR Clean-up kit (Macherey Nagel), according to the manufacturer’s instructions. Purified products were examined with the Agilent 4200 TapeStation (Agilent Technologies) using the Agilent D5000 ScreenTape system. The concentration of each purified product was quantified with a Qubit 4 fluorometer (Invitrogen) using the Qubit broad-range assay.

The purified RT-PCR products were used to prepare sequencing libraries with a Nextera XT DNA sample preparation kit (Illumina), according to the manufacturer’s instructions. All libraries were sequenced on a MiSeq (Illumina) using the MiSeq v3 chemistry according to the manufacturer’s protocol, producing 2×250 bp paired-end reads. All generated WGS data have been deposited in the National Center for Biotechnology Information (NCBI) Sequence Read Archive (SRA) [33] under BioProject accession number PRJNA615341.

### Generation of consensus genome sequences

Raw (paired-end) reads were trimmed using Trimmomatic v0.32 [34] with the following settings: ‘ILLUMINACLIP:NexteraPE-PE.fa:2 : 30 : 10’, ‘LEADING: 10’, ‘TRAILING: 10’ ‘SLIDINGWINDOW: 4 : 20’, and ‘MINLEN: 40’ retaining only paired-end reads. For each sample, a suitable reference genome for read mapping was selected from the NCBI viral genomes resource [35] (Supplementary Methods). Consensus sequences for all samples were obtained following the GATK ‘best practices’ protocol (Supplementary Methods). All 253 generated consensus genomes were deposited in the GISAID database (i.e. samples EPI_ISL_415199 to EPI_ISL_415452) [19]. Sequencing coverage was extracted for each position from each sample using SAMtools depth 1.3.1 [36] and positions were normalized against the vaccine strain length. The percent identity matrix was calculated using the online muscle program hosted by the EBI (European Bioinformatics Institute) [37] for each segment.

### Phylogenomic analysis

The WHO/ECDC provide a list of references representing each HA clade [38], but provisional clustering tests indicated that these did not allow the defining of phylogenetic groups for all samples. Additional reference sequences were selected from phylogenetic trees in ECDC reports and Nextstrain (www.nextstrain.org) [39] (Table S1, available in the online version of this article). Alignments of sequenced samples and reference sequences were generated for all segments employing mega 7.0.18 [40] using default parameters for ClustalW [41] alignment. Only protein-encoding sequences of each segment were retained. beast v1.10.4 [42] was used to create phylogenetic trees for every segment individually, and the whole genome, with underlying evolutionary model and other settings as listed in detail in the Supplementary Methods. Maximum clade credibility trees were generated afterwards using the TreeAnnotator program of beast with default settings. Generated trees were visualized in iTOL (https://itol.embl.de/login.cgi) [43].

A local Nextstrain instance [39], allowing lightweight phylogenomics comparison with much more genomes than possible with beast, was built using sequenced samples complemented with GISAID sequences. Only those GISAID whole genomes were retained that included patient sex and age information, directly sequenced without passaging in cells or eggs, resulting in 14 157 genomes. All sequences were aligned with CLC Genomics Workbench 20.0.2 with default parameters, and UTRs (untranslated regions) were stripped. Aligned segments were concatenated into a single sequence for all samples retaining only sequences with ˂3 gaps and/or ‘N’ characters. Genomes were clustered based on sequence identity with cd-hit 4.6.8 using different cut-offs to retrieve ~3000 genomes, which was reached at 99.83 % sequence identity. Sequenced Belgian samples were retained irrespective of their sequence similarity (Table S2). The local Nextstrain instance was then constructed as detailed in the Supplementary Methods. The generated tree was visualized in R using the packages ‘ggtree’ and ‘phytools’.

### Reassortment detection

**Fig. 1.**
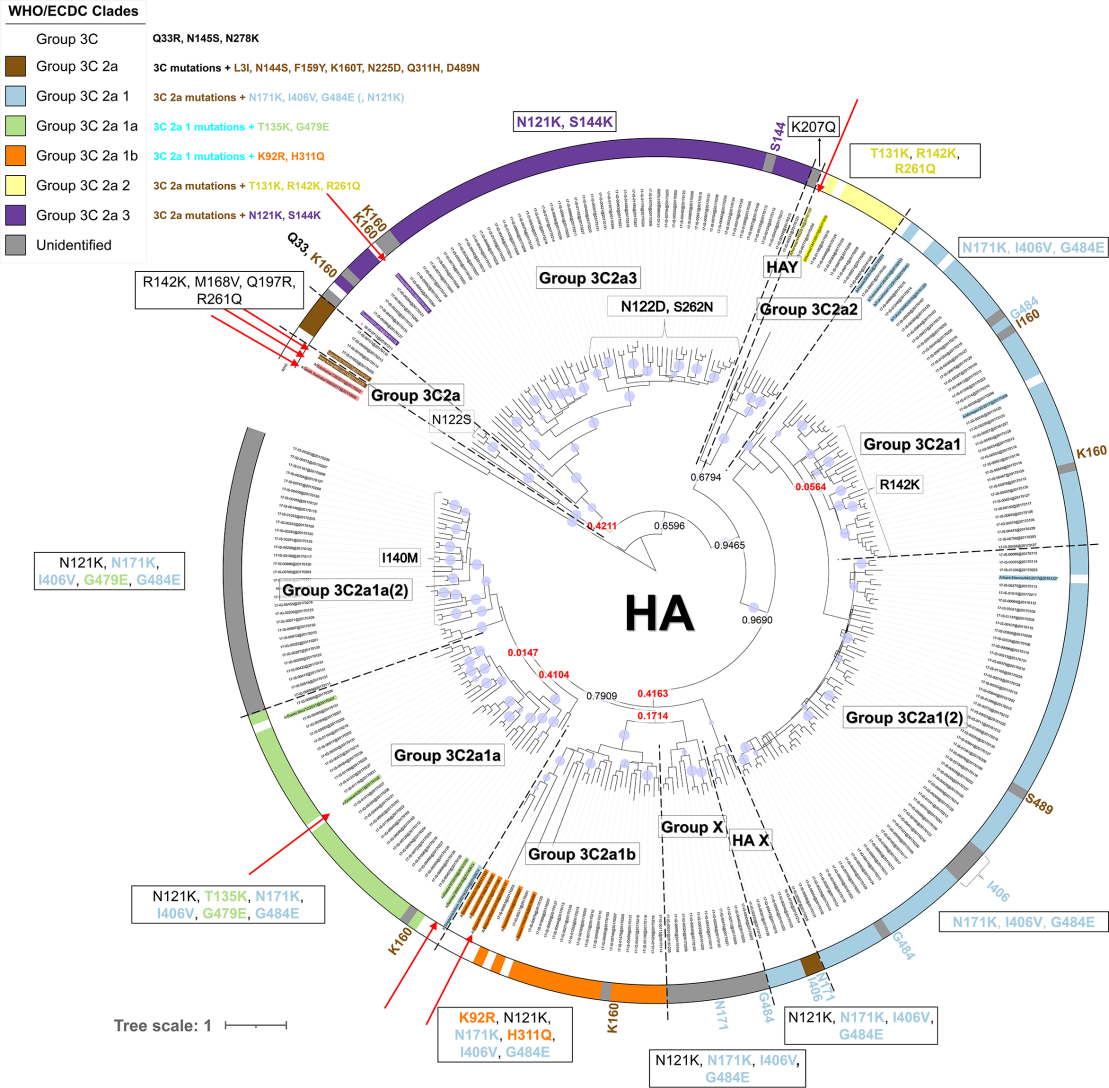
Phylogenetic HA gene tree. Red arrows indicate (coloured) reference names assigned to the various groups as defined by the WHO/ECDC, and coloured names without red arrows indicate additional references selected from ECDC reports and nextstrain.org. Specific amino acid substitutions designated to groups according to WHO/ECDC guidelines are indicated on the figure, and the circular coloured outer strip around the tree represents the assigned groups based on amino acid substitutions defined in the WHO/ECDC guidelines, according to the colour legend. Within the tree, the group labels represent the 11 phylogenetic groups that were assigned to their respective samples according to their classification based on references (coloured names) and the support of nodes by posterior probability values. Group ‘X’ clustered together in a separate cluster from the other groups. Groups ‘HAX’ and ‘HAY’ contain samples that could not be classified. Posterior probability values are indicated on key nodes that separate groups, and are coloured red if below 0.5. The size of the blue discs on nodes represents the posterior probability scaled between 0.5 and 1. The scale bar represents the mean number of substitutions per site.

**Fig. 2.**
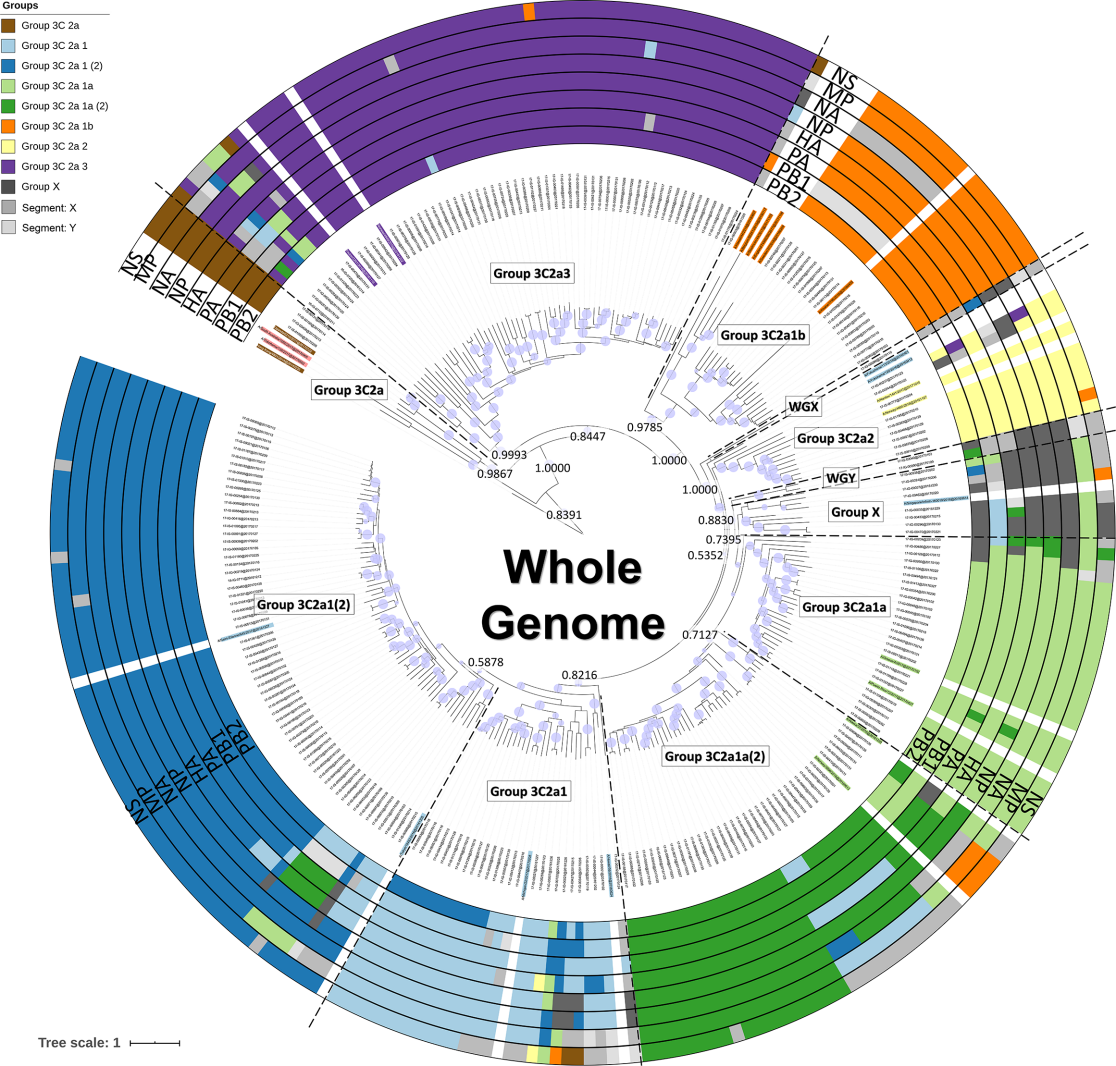
Phylogenetic tree based on the whole genome. Coloured names indicate additional references selected from ECDC reports and nextstrain.org. Coloured rings around the tree represent classification results for the eight segments separately (Fig. 2 for the HA gene, and Figs S2–S8 for the seven other genes). Group ‘X’ clustered together in a separate cluster from the other phylogenetic groups. Groups ‘WGX’ and ‘WGY’ contain samples that could not be classified. Groups labelled with the segment name and a single letter (e.g. PB1X) similarly represent any remaining samples that could not be confidently assigned into phylogenetic groups according to their segment trees (Figs S2–S8). Within the tree, the group labels represent the phylogenetic groups that were assigned to their respective samples according to their classification based on references (coloured names) and the support of nodes by posterior probability values. Posterior probability values are indicated on key nodes that separate phylogenetic groups. The size of the blue discs on nodes represents the posterior probability scaled between 0.5 and 1. The scale bar represents the mean number of substitutions per site.

For manual reassortment detection, individual segment trees (Figs 1 and S2–S8) obtained with beast were compared visually to the whole-genome tree (Fig. 2), and reconciliation of topologies was sought between the eight influenza segment trees. When a topological inconsistency in sample positioning in one of the segment trees was present, indicative of belonging to another phylogenetic group, the posterior probability value of the ancestral nodes in the segment tree between the group of the sample defined by the whole-genome tree and the group in which the reassorted genome was present was checked. Only if this posterior probability value was ≥0.95, was the genome retained as an intra-subtype reassortant. Reassortment detection was also computationally independently performed using Graph-incompatibility-based Reassortment Finder (GiRaF) software v1.02 [44]. Tree files from two randomly selected replicates of the whole-genome analysis produced by beast were downsampled to 1000 trees and analysed with GiRaF using default settings. Only reassortments with a confidence level ≥0.95 were accepted. Subsequently, a consensus approach was applied by retaining only reassortments detected by both methods. To estimate the reassortment frequency, the number of reassortant genomes was divided by the total number of genomes.

### Inference of host characteristic associations

All statistical analyses were performed using R software (RStudio 1.0.153; R3.6.1). The two-sided Fisher’s exact test was used to assess associations between host characteristics and phylogenetic groups for both the whole genome and each segment individually. The same analysis was performed to assess associations between severity outcome and reassortment presence/absence. Host characteristics included infection severity (classified into mild, moderate and severe), patient age (categorized into <15, 15–59 and ≥60 years), sampling date (before, during and after the epidemic peak), vaccination status, presence of comorbidities and severity indicators. Multiple testing correction was conducted by applying the Benjamini–Hochberg method [45] and controlling the false discovery rate (FDR) at 5 %. For statistically significant associations identified during the univariate analysis, multiple linear regression was used to identify potential confounding variables and effect modifications.

Permutation analyses were performed to investigate the effect of different sample sizes for ILI (*n*=93) and SARI (*n*=160) cases. SARI and ILI samples were randomly selected 10 000 times with replacement for *x*=1, 2, …, 93 cases of both the total ILI and SARI population. In addition, at *x*=93, a two-sided permutation test at a significance level of 0.05 was performed (i.e. attempting to answer the question, does the observed value for 93 samples lay within the 95 % confidence interval when 10 000 times 93 samples are randomly selected with replacement from the respective ILI/SARI case population?). All analysis scripts are provided in the Supplementary Material.

## Results

### WGS of clinical influenza A(H3N2) samples

Whole-genome A(H3N2) sequences were successfully obtained for all 253 samples used in this study. Fig. S1 provides an overview of the sequencing coverage for each position from each sample. Obtained sequences had a median depth (i.e. number of times each base shows up in individual reads) and breadth (i.e. total recovered genome sequence length) of coverage >9077× and >99 %, respectively. Sequencing efficiency varied slightly inversely with segment size, with smaller fragments such as the M segment generally displaying a slightly greater depth. For two samples only, a part of the genes encoding PB2, PB1 or NP had a coverage <100×. Consequently, all samples were retained for further analysis.

### Classification using WHO/ECDC guidelines for the HA segment

Samples were first classified according to WHO/ECDC guidelines by creating a phylogenetic tree with beast software [42] for the HA segment. The terms ‘clade’ and ‘phylogenetic’ group specifically refer to grouped samples using either the current or our newly proposed classification method, respectively, whereas the term ‘genetic group’ refers to samples that cluster together without specifically considering the classification method. WHO/ECDC provide a list of influenza references representing each HA clade [38], but provisional clustering tests indicated that these did not allow proper definition of the Belgian samples within clades (data not shown). Therefore, more reference sequences were selected manually from phylogenetic trees in ECDC reports and nextstrain.org [39] by building provisional trees to evaluate whether the Belgian samples could be classified. Fig. 1 presents the resulting phylogenetic tree based on the HA segment obtained with beast for all sequenced samples and employed additional reference/vaccine sequences. Classification following the WHO/ECDC guidelines based on clustering with reference and vaccine strains (references provided by WHO/ECDC are indicated specifically with red arrows in Fig. 1), and identification of specific amino acid substitutions linked to clades, resulted in the identification of four clades. In total, 26, 18, 7 and 49 samples, respectively, could be attributed to the HA groups ‘3C2a1a’, ‘3C2a1b’, ‘3C2a2’ and ‘3C2a3’. Four samples clustered with a WHO/ECDC reference for group ‘3C2a’, but were phylogenetically too distant to motivate their inclusion in this group. The employed WHO/ECDC reference for group ‘3C2a1’ did not cluster with samples that should belong to this group, but rather with samples of group ‘3C2a1a’. Belgian samples belonging to group ‘3C2a1’, however, could be classified because they clustered with some of the additional reference strains that were selected from ECDC reports and nextstrain.org [39]. In total, the approach using solely WHO/ECDC information resulted in 153 unassigned samples, of which 59 lacked at least one clade-defining amino acid substitution (highlighted by a grey strip in Fig. 1) and 144 did not cluster with any reference strain provided by WHO/ECDC guidelines.

Considering the support of nodes by posterior probability values in relation to the additionally employed references (indicated in Fig. 1 with the colour of their corresponding phylogenetic group but without a red arrow), and specific additionally identified substitutions, resulted in classification of 11 phylogenetic groups. Group ‘3C2a’ could now be defined because samples clustered properly with one of the additionally selected references that was defined as clade ‘3C2a’. Group ‘3C2a1’ and group ‘3C2a1(2)', and group ‘3C2a1a’ and group ‘3C2a1a(2)’, are phylogenetically closely related to each other, but were both split into two subparts denoted with the suffix ‘(2)’ because they were clearly delineated in the phylogeny, albeit supported by nodes with low posterior probability values. Additionally, most samples of group ‘3C2a1’ possessed an additional amino acid substitution R142K compared to group ‘3C2a1(2)’, and group ‘3C2a1a(2)’ lacked the clade-specific substitution T135K of group ‘3C2a1a’. Group ‘X’ consists of samples that similarly clustered together in most segment trees, except the PB1 and M trees. Groups ‘HAX’ and ‘HAY’ include samples difficult to classify into the other phylogenetic groups. The classification uncertainty of these samples was often reflected in other segment trees. Additionally, these samples possess different mutations in the HA segment and in the whole genome compared to samples in other phylogenetic groups, which supports their designation to separate phylogenetic groups.

### Using whole-genome sequences and beast allows improved phylogenetic classification

WGS enables construction of phylogenies for the other seven segments separately, as well as the combined whole genome. A phylogenetic tree was constructed with beast using the whole-genome sequences of the Belgian isolates rather than solely the HA segment, after which classification was performed similarly by considering the support of nodes by posterior probability values in relation to employed reference/vaccine genomes, and specific additionally identified substitutions. The resulting classification is presented in Fig. 2. Additionally, the same approach for the other seven segments was individually applied, and is presented in Figs S2–S8. Comparison of the HA (Fig. 1) and whole-genome (Fig. 2) trees indicates that approximately 83.4 % of samples were classified in the same phylogenetic groups. Additionally, the whole-genome tree overall has higher posterior probability values [posterior(nodes) ≥ 0.5 : 203 (whole genome) vs 94 (HA)], increasing confidence in the overall topology, because the posterior probability of a tree is the probability that the tree is correct given the data if the underlying model is correct [46, 47].

### Increasing the number of genomes considered with a custom-built Nextstrain instance

**Fig. 3.**
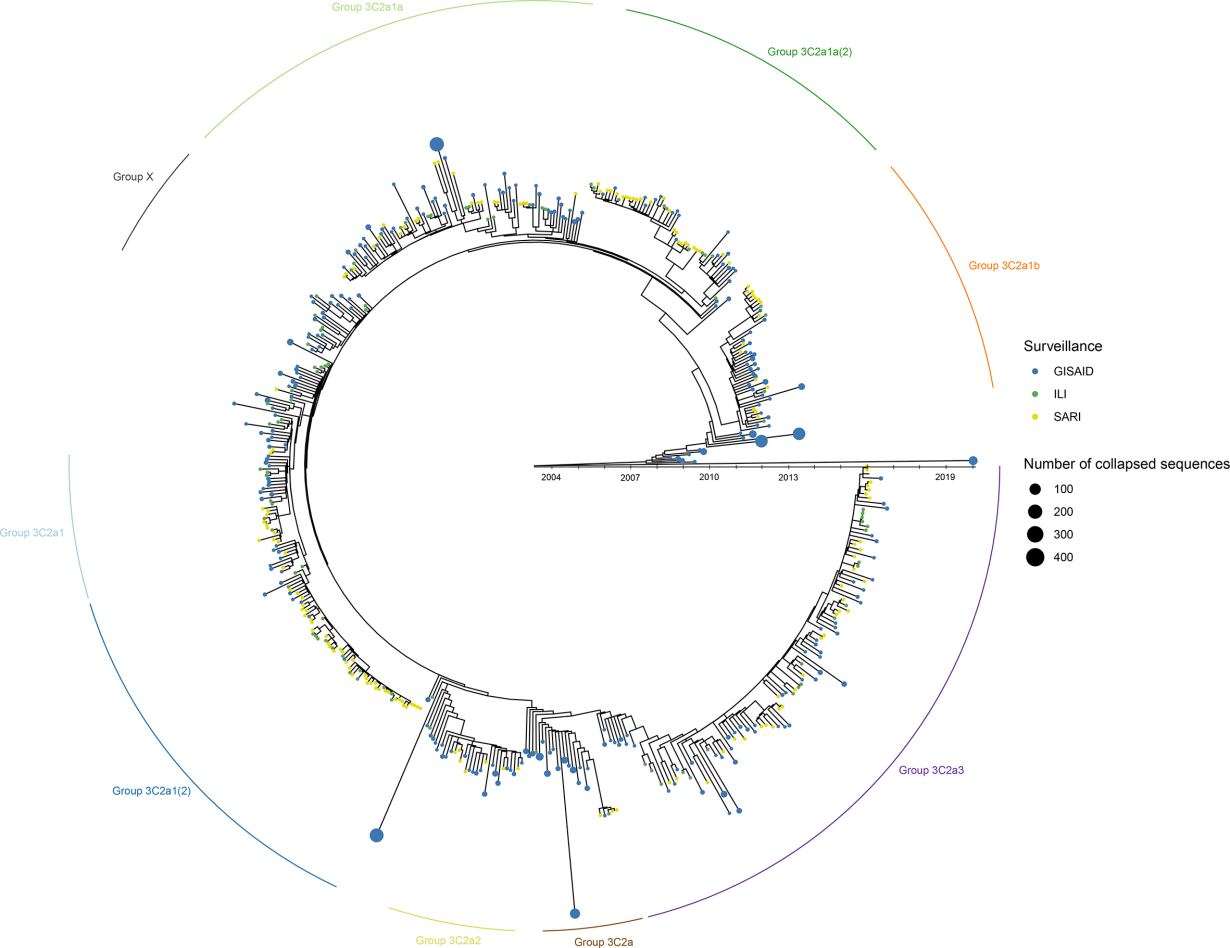
Time-resolved overview of influenza samples from the Belgian 2016–2017 outbreak season in the context of globally circulating influenza strains based on an in-house Nextstrain instance using only whole-genome sequences. Green- and yellow-coloured dots represent ILI and SARI samples sequenced in this study, respectively. Blue coloured dots represent GISAID samples. If an ancestor only included GISAID sequences, these nodes were collapsed for better visualization with the size of such nodes proportional to the number of included samples. Phylogenetic groups based on the whole genome (Fig. 2) are indicated around the tree. The branch lengths correspond to the sampling date of the sample. In case of grouped GISAID samples, the sampling date of the latest sample is used.

Proper reference selection was extremely arduous, requiring multiple explorative analysis iterations to select suitable reference genomes allowing classification to arrive at the phylogenies and resulting classification presented for HA (Fig. 1) and the whole genome (Fig. 2). An alternative approach was explored by using Nextstrain to reconstruct an in-house instance of the Belgian samples supplemented with more than 2500 publicly available A(H3N2) genomes available in the GISAID database, because Nextstrain is a framework meant for real-time analysis (i.e. as an outbreak is ongoing) of several hundreds to even thousands of genomes. This allowed positioning of the Belgian samples within the globally circulating context. When comparing the custom-built Nextstrain tree (Fig. 3) with the whole-genome tree of the Belgian samples (Fig. 2), 217 Belgian samples clustered congruently in both trees. Six samples from group ‘X’ clustered together in a separate cluster. The remaining 30 samples were placed differently between both trees. The high congruence between both trees enabled to more easily identify suitable references. Fig. 3 illustrates considerable diversity amongst the Belgian samples, belonging to five major phylogenetic groups: (i) samples belonging to group ‘3C2a3’; (ii) group ‘3C2a1’ and group ‘3C2a1(2)'; (iii) group ‘3C2a1a’ and group ‘3C2a1a(2)'; (iv) group ‘3C2a1b’; and (v) group ‘3C2a2’. Group ‘3C2a1’ and group ‘3C2a1(2)', and group ‘3C2a1a’ and group ‘3C2a1a(2)', formed two distinct clusters each time, supporting their separation into different phylogenetic groups as previously suggested by both the HA (Fig. 1) and whole-genome (Fig. 2) trees. Lastly, four samples belonged to an additional phylogenetic group corresponding to group ‘3C2a’. The root for the samples from the Belgian 2016–2017 outbreak season goes back to 2003–2004, and phylogenetic groups containing Belgian samples are interspersed with many samples isolated in other countries, indicating that phylogenetic groups are not limited by country boundaries and that these groups have already co-circulated in the Belgian population for several years.

### Detection of intra-subtype reassortments

**Fig. 4.**
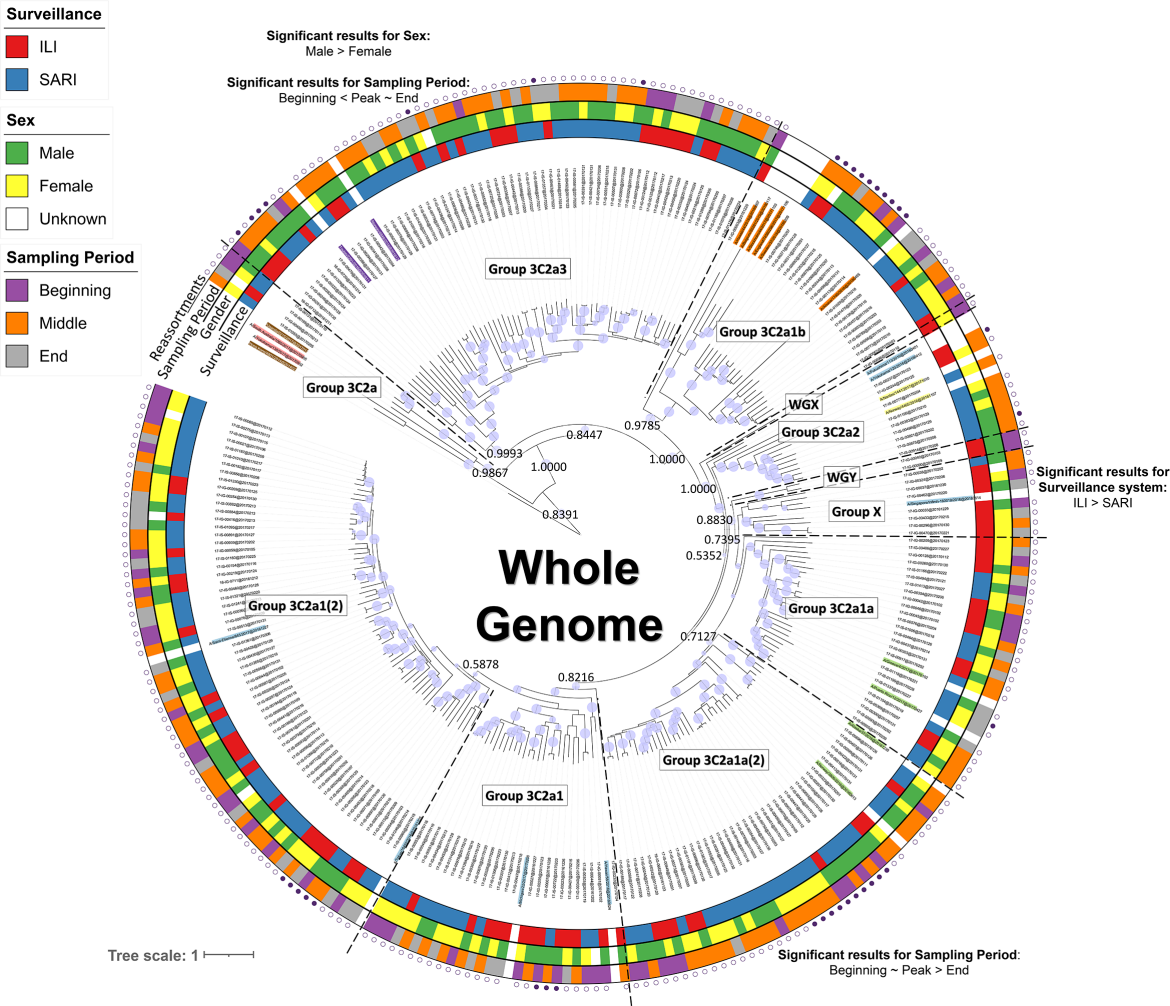
Phylogenetic tree based on the whole-genome annotated patient data for which significant associations with phylogenetic groups were detected. Coloured taxon labels indicate additional references selected from ECDC reports and nextstrain.org. Coloured rings around the tree represent patient data, including the surveillance system, sex and sampling period, for samples where this information was available. Around the outside of these strips, the presence (filled circle) or absence (empty circle) of reassorted genomes is indicated based on the consensus of both manual inspection and computational analysis with GiRaF. Statistically significant results using the Fisher’s exact test with FDR correction for associations between host characteristics and sample parameters with the newly defined phylogenetic groups for the whole genome (see Fig. 2) are presented on the figure near their respective phylogenetic group. Results for individual segments and more detailed information, including the effect size and confidence interval, are presented in Table S4. The host characteristics and sample parameters for the reference genomes were excluded. Posterior probability values are indicated on key nodes that separate phylogenetic groups. The size of the blue discs on nodes represents the posterior probability scaled between 0.5 and 1. The scale bar represents the mean number of substitutions per site.

WGS enabled identification of (intra-subtype) reassortments within the Belgian A(H3N2) samples, allowing for investigation of the influence of reassortments on viral evolution through both manual inspection by visually comparing individual segment trees obtained with beast to the whole-genome tree, as well as through computational analysis by using the GiRaF software. For both methods, the genome was retained as an intra-subtype reassortment if the posterior probability value was ≥0.95. Results of the combination of the manual and computational analysis are visualized in Fig. 4, and a detailed list of reassortments detected by both manual inspection and computational methods is provided in Table S3. Using the combination of the manual and computational approach, 38 strains were characterized as intra-subtype reassortants resulting in an intra-subtype reassortment rate of 15.02 %. With the manual detection method, 57 reassorted genomes were detected (22.53 %), whereas with GiRaF 39 reassorted genomes were identified (15.42 %).

No statistically significant result was obtained using the Fisher’s exact test between the surveillance system and reassortment status. Permutation analyses were performed to investigate the association between disease severity and reassortment status while correcting for the different sample sizes of ILI and SARI samples. For all sample sizes ranging from 1 to 93 (the maximum number of ILI samples), the number of reassorted genomes was consistently higher for SARI samples compared to ILI samples (see Supplementary Materials). Moreover, at a sample size of 93, a two-sided permutation test at a significance level of 0.05 indicated that significantly more reassorted genomes were present in the SARI population (*P* value=0.0208; Supplementary Materials).

### Associations between host characteristics and phylogenetic groups

Associations between host characteristics and phylogenetic groups for both the whole genome and each segment individually were inferred using the Fisher’s exact test with FDR correction. Statistically significant results for associations between phylogenetic groups and host characteristics are presented in Fig. 4 for the whole genome. Results for the individual segments are presented in Table S4. Significantly more male than female patients were infected with strains belonging to group ‘3C2a3’ not only for the whole genome, but also for the HA segment with the same confounding effects (i.e. variables that have an influence on the correlation between the group and the host characteristic) (Fig. 4, Table S4). Group ‘X’ mainly consists of samples from ILI cases not only for the whole genome but also for the HA segment with the same confounding effects (Fig. 4). A significant association was detected between group ‘3C2a1a(2)’ and sampling period for the whole genome, with samples being present mainly during the seasonal beginning and peak, but decreasing in presence towards the end. Although this association was not detected for the HA segment, it was detected when samples were classified according to the PB2 and PB1 segments (Table S4). A significant association between group ‘3C2a3’ and sampling period for the whole genome was also detected, with an increasing number of samples towards the end. This significant association was also detected when samples were classified according to the PB1 segment (Table S4). Although not significant, group ‘3C2a1a’ was emerging during the course of the influenza season (Fig. S9), while group ‘3C2a2’ only appeared during the seasonal peak. Several additional significant associations were detected between host characteristics and the individual segments (Table S4). All genomes were assessed regarding antiviral resistance by comparing to a previously composed database of antiviral resistant mutations [16], but no known antiviral resistance mutations were observed within the genomes (results not shown).

## Discussion

The field of microbiology is transforming due to the decreasing turnaround times and costs, and increasing availability, of whole-genome information and user-friendly data analysis tools. The added value of genomic surveillance has been showcased during the SARS-CoV-2 pandemic by tracking the virus, detecting emerging variants of concern, and applying the genomic data to a wide variety of associated biological questions [48]. As the COVID-19 restrictions also limited the spread of influenza, genomic surveillance of influenza is likely to become even more important when COVID-19 restrictions ease [49]. Although the HA gene remains the principal region for Sanger sequencing in classical influenza surveillance programmes, this study illustrates the feasibility and benefit of switching towards WGS-based surveillance. Sanger sequencing the HA gene has a lower cost compared to WGS, but when several samples are multiplexed in the same WGS run, the overall cost of the latter is lower compared to Sanger sequencing every gene separately [19]. This study proposed an improved methodology for classification of circulating strains with higher resolution for genetic characterization and reassortment detection. Moreover, integration of WGS data with patient information allowed investigation of the associations of phylogenetic groups based on the whole genome with host characteristics.

Influenza classification according to WHO/ECDC guidelines is currently based on phylogenetic analysis of the HA segment through clustering with vaccine/reference strains and detection of predefined amino acid substitutions [8]. However, this approach enabled only classification of a small subset of the Belgian influenza 2016–2017 outbreak samples because the majority of samples did not cluster with reference strains and/or lacked specific clade-defining HA amino acid substitutions. Our study evaluated the feasibility of shifting classification for influenza surveillance to a whole-genome-based approach rather than solely the HA gene, also incorporating many more references selected from ECDC reports and nextstrain.org [39], to improve definition of genetic groups. We have demonstrated that employing whole-genome information offers improved classification performance because more samples could be characterized into well-supported phylogenetic groups compared to only considering the HA gene.

Current surveillance programmes typically employ relatively simple phylogenetic tree reconstruction methods based on distance estimation and maximum parsimony, such as mega neighbour joining [50, 51]. More advanced methods such as RAxML maximum likelihood are used only rarely [8]. In contrast, phylogenetic tree construction through Bayesian inference has emerged as a standard in recent years for fundamental viral genomics studies. This powerful but resource-intensive approach allows robust phylogenomic investigation and facilitates deep exploration of the circulating genetic diversity [52, 53]. Here, beast software [42] was used, which takes phylogenetic uncertainty into account and incorporates complex evolutionary models [54]. beast was, for instance, used to find the origin and map avian influenza A(H7N9) diversity causing human infection [55], simulating real-time evolutionary rate estimates and dating the emergence and intrinsic growth rate of the A(H1N1)pdm09 pandemic [56]. Our case study, therefore, demonstrates the feasibility of switching to more robust phylogenetic tree reconstruction based on Bayesian inference for genetic influenza surveillance. Ideally, more rigid model selection is performed for validating the underlying model assumptions, but this still requires computational resources beyond the capacity of the Belgian, and most other National Reference Centers (NRCs).

Being time-consuming and computationally intensive, Bayesian inference is, however, infeasible for larger datasets containing several hundreds of samples or if a quick response is required. Selection of suitable genome references for phylogenetic classification poses another challenge. Therefore, an alternative approach was explored using a custom-built Nextstrain instance, which offers several advantages. Nextstrain can analyse several hundreds to thousands of genomes much faster compared to beast and presents a real-time view. Although the public influenza instance hosted at nextstrain.org [39] is based solely on the HA and NA genes, we demonstrated the feasibility of using whole-genome information. The feasibility of using Nextstrain with whole genomes also allows provisionally assigning samples to already existing or newly emerging groups to select suitable references to perform more powerful phylogenomics investigation with methods such as beast.

Consistent with previous studies, we found co-circulation of different A(H3N2) phylogenetic groups during influenza epidemics [52, 57, 58]. A total of 211 of 253 samples (83.4 %) classified based on the HA segment belonged to the same phylogenetic group based on the whole genome, suggesting that the other seven segments contribute important additional genetic information. Intra-subtype reassortments were observed at a rate of ~15 % in the Belgian 2016–2017 season, likely facilitated by the co-circulation of several phylogenetic groups. Although intra-subtype reassortment detection remains challenging, posterior probability values derived from beast aided reassortment detection with both manual inspection and computational approaches to avoid selecting uncertain reassortments. Intra-subtype reassortments are currently typically studied using manual inspection of different segment phylogenetic trees by checking for positional inconsistencies. This is highly time-consuming, error-prone and aggravated by unclear phylogenetic trends in segments that exhibit limited genetic diversity. Recent subtle reassortments are more challenging to detect because sequences from the same subtype are more similar [44, 59]. Through our strict requirements for reassortment detection by both a computational and manual method requiring high support values, the resulting reassortment rate of 15 % is likely underestimated. Goldstein *et al*. [52] studied A(H3N2) in Scotland for the 2014–2015 season, and observed less intra-subtype reassortments (5.3 %) using a similar methodology. However, Berry *et al*. [15] studied reassortments in 2091 A(H3N2) globally circulating strains collected between 2009 and 2014, and observed a higher rate of intra-subtype reassortments (39.1 %) by using a computational approach. However, it should be noted that, similar to recombination for bacterial pathogens, reassortment can disturb the true phylogenetic signal in whole-genome-based phylogenetic investigations because it disturbs the underlying assumptions of the tree model, resulting in biased topologies. One strategy to circumvent this is by removing all reassorted samples, but this would typically remove large proportions of the input dataset for influenza due to its relatively high rate of reassortment. Therefore, a need exists for development of new tools and models that can take this effect into account, such as the Bacter package in beast2 developed for bacterial pathogens [60].

SARI samples were found to be more likely to comprise reassortments in agreement with Goldstein *et al*. [52]. Nelson *et al*. similarly found that reassortments could potentially trigger emergence of unusually severe seasonal A(H1N1) epidemics [61]. Several statistically significant associations were detected between patient data and the whole genomes that could not be identified using solely HA data: more males were associated with group ‘3C2a3’ and more samples occurred near the seasonal beginning in group ‘3C2a1a(2)', which was observed for neither the HA nor NA segments, but rather the PB2 and PB1 segments, suggesting that the potential value of other segments is undervalued. Other examples include more ILI than SARI cases for group ‘3C2a1a’ for the M segment, and more intensive care unit (ICU) than non-ICU cases for group ‘3C2a1a’ for the NA segment. Several other host characteristic associations with particular segments were observed (Table S4). Additional information on associations of certain groups with host characteristics can be potentially useful, for instance, to detect groups more prone to result in severe disease allowing implementation of preventive measures. To the best of our knowledge, no other studies have explored the relationship between phylogenetic groups and host data for influenza. However, it should be noted that this dataset includes a limited number of samples as sequencing all samples would still be too expensive. Although all patient and sample information was used in the analyses, the samples were selected based on the severity, patient age and sampling date. Other patient and sample information was occasionally unknown in addition to an unequal distribution for some parameters. Additionally, some phylogenetic groups contained a limited number of samples. Consequently, the sample selection was underpowered for some parameters, which could be mitigated by a larger sample dataset.

Our study illustrates that genetic surveillance should gradually shift to WGS for seasonal influenza surveillance. WGS enables employment of powerful phylogenomics methods that substantially improve phylogenetic classification, thereby providing more information to national influenza prevention and control programmes regarding the timing, impact and severity of seasonal epidemics. WGS can also improve vaccine strain selection, which will be especially relevant for next-generation vaccines that do not focus solely on the HA segment. Bayesian inference using beast facilitates reassortment detection with both manual inspection and computational methods, enabling investigation of intra-subtype reassortment effects on public health. Tools optimized for real-time analysis, such as Nextstrain, facilitate contrasting seasonal local outbreaks to the globally circulating context and can provide a quick response. Lastly, incorporating whole-genome information allows association of phylogenetic groups with host characteristics with particular epidemiological value, such as disease severity. Future research should consider investigating whether the high diversity within the A(H3N2) influenza subtype should be considered by using the phylogenomic groups to study mutations found in the genomes.

## Supplementary Data

Supplementary material 1

Supplementary material 2
