# Supplementary material for: Whole-genome-based phylogenomic analysis of the Belgian 2016–2017 influenza A(H3N2) outbreak season allows improved surveillance
Source: Microb Genom. 2021 Sep 3;7(9):000643. doi: 10.1099/mgen.0.000643 (PMC8715427; doi:10.1099/mgen.0.000643)
Supplement: Supplementary material 1 [file mgen-7-0643-s001.pdf]

Table of Contents

Supplementary Methods 3
Methods 3
References 9
R scripts used for statistical analysis 12
Libraries 12
Read in data 12
Groups vs Metadata 12
Reassormtents vs Metadata 14
Fisher tests 14
WG 14
PB2 15
PB1 16
PA 17
HA 18
NP 19
NA 21
MP 22
NS 23
Reassortments 24
Permutation tests 25
Reassortments 25
Supplementary Tables and Figures 29
Table S1: Additional reference sequences used for phylogenomic analysis 29
Table S2: Sequences extracted from GISAID to construct the custom-built Nextstrain instance 30
Table S3: Detailed list of reassortments detected by both manual inspection and computational methods 42
Table S4: Statically significant results using the Fisher's exact test with FDR correction for association between host characteristics and sample parameters with the newly-defined phylogenetic groups for the whole-genome and all the individual segments. 46
Figure S1: Coverage depth and breadth for the 253 sequenced Influenza A(H3N2) samples using WGS stratified per segment. 47
Figure S2: Phylogenetic tree based on the PB2 gene. 48
Figure S3: Phylogenetic tree based on the PB1 gene. 49

Figure S4: Phylogenetic tree based on the PA gene. 50
Figure S5: Phylogenetic tree based on the NP gene. 51
Figure S6: Phylogenetic tree based on the NA gene. 52
Figure S7: Phylogenetic tree based on the M gene. 53
Figure S8: Phylogenetic tree based on the NS gene. 54
Figure S9: Proportion of phylogenetic groups based on the whole genome tree per sampling period. 55

Supplementary Methods

Methods

Sample selection. Two main surveillance systems exist in Belgium, ILI for mild infections and SARI for moderate and severe infections. ILI cases are defined by a sudden onset of symptoms, including fever and respiratory and systemic symptoms. A SARI case is an acute respiratory illness with onset within the last 10 days of fever, respiratory symptoms, and requiring hospitalization. These two surveillance systems are essential to follow trends of viral spread and changes in circulating influenza viruses. In the 2016-2017 season (week 40-2016 to week 20-2017), 646 ILI samples were collected by sentinel general practitioners and sent to the Belgian National Influenza Centre (NRC), of which 323 tested positive for Influenza A(H3N2) based on routine diagnostic surveillance with qPCR [1]. From SARI surveillance, 1,422 samples with correct information and corresponding to the case definition were collected during the 2016-2017 influenza season by six sentinel hospitals located across the country and similarly sent to the NRC, of which 526 tested positive for Influenza A(H3N2). A standard survey accompanied all samples with patient information on sex, birth date, clinical features, vaccination status, administration of antiviral treatment or antibiotics, date of symptom onset and date of sample collection. More information about the host data and sampling information can be found in the main manuscript.

RNA isolation, PCR amplification and WGS. The samples were extracted as described in the main manuscript. Sequencing amplicons were generated in a one-step RT-PCR, in a 50 µL reaction volume with a combination of three primers that allow to reverse transcribe and amplify each segment of the Influenza A genome. This protocol is based on Van den Hoecke et al. (2015) [2] with optimized volumes and RT-PCR conditions. Primers included CommonA-

Uni12G (GCCGGAGCTCTGCAGATATCAGCGAAAGCAGG), CommonA-Uni12 (GCCAGAGCTCTGCAGATATCAGCAAAAGCAGG) and CommonA-Uni13G (GCCGGAGCTCTGCAGATATCAGTAGAAACAAGG) [2]. Reaction volumes contained 17.375 µL water, 25 µL 2X RT-PCR buffer, 1 µL SuperScript III One-Step RT-PCR Platinum® Taq HiFi DNA Polymerase (Invitrogen, USA), 0.5 µL RnaseOUT Recombinant Ribonuclease Inhibitor (Invitrogen, USA), 0.375 µL of each primer (20 µM) and 5 µL of RNA extract. The following thermocycler conditions were used: one cycle at 42°C for 15 minutes, one cycle at 55°C for 15 minutes, one cycle at 60°C for 5 minutes, one cycle at 94°C for 2 minutes (ramp rate: 2.5 °C/s); 5 cycles at 94°C for 30 seconds, 45°C for 30 seconds (ramp rate: 2.5 °C/s) and 68°C for 5 minutes (ramp rate: 0.5 °C/s); 37 cycles at 94°C for 30 seconds, 55°C for 30 seconds and 68°C for 5 minutes; and one cycle at 68°C for 5 minutes (ramp rate: 2.5 °C/s). Finally, the purified RT-PCR products were sequenced according to the instructions in the main manuscript.

Generation of consensus genome sequences. Raw (paired-end) reads were first trimmed using Trimmomatic v0.32 [3] setting the following options: 'ILLUMINACLIP:NexteraPE-PE.fa:2:30:10', 'LEADING:10', 'TRAILING:10' 'SLIDINGWINDOW:4:20', and 'MINLEN:40'. Only paired-end reads were retained for further analysis. A suitable reference genome for read mapping was selected from the NCBI viral genomes resource [4] for each sample as follows. Firstly, an in-house cleaned database dump for influenza A was created using in-house scripts to: (i) standardize sequence identifiers, (ii) remove incomplete sequences, (iii) remove sequences with ambiguous or inconsistent annotations, (iv) and remove sequences containing >1% and/or a stretch ≥5 consecutive 'N' characters. For every segment, all sequences deviating more than one standard deviation from the mean length were removed. The remaining sequences were clustered with CD-HIT v4.6 [5] using a sequence identity cutoff

of 0.98 to reduce sequence redundancy by retaining only the representative sequence for every cluster. Secondly, the best segment reference in this cleaned database was selected for every sample based on following procedure: 10% of (trimmed) reads were randomly selected using seqtk v1.2 (https://github.com/lh3/seqtk). High-quality alignments of subsampled reads against the cleaned database were identified with Blastn v2.6.0 [6] using the following criteria: a sequence identity ≥97%, containing no gaps, and a query sequence coverage ≥85%. The database sequence containing the highest number of high-quality alignments was retained as reference for read mapping with BWA-MEM v0.7.15 (Li et al., 2009) using default settings. Consensus sequences for all samples were afterwards obtained following the GATK 'best practices' protocol [7] with Picard v2.8.3 (https://broadinstitute.github.io/picard/) and GATK v3.7 as follows. Firstly, duplicated reads were marked with PICARD MarkDuplicates before performing indel realignment with GATK and variant calling using the GATK UnifiedGenotyper with the following options: '-ploidy 1', '--stand_call_conf 30', and '--genotype_likelihoods_model BOTH'. GATK VariantFilter was used to retain only high-quality variants with a read depth ≥200. Secondly, the consensus sequence was obtained based on the called variants and selected reference sequence utilizing GATK FastaAlternateReferenceMaker. All 253 generated consensus genome sequences have been deposited in GISAID (samples EPI_ISL_415199 to EPI_ISL_415452) [8]. To extract the sequencing coverage for each position from each sample, Samtools depth 1.3.1 [9] was used on the bam files and positions were normalized against the vaccine strain length. The percent identity matrix was calculated using the web-based MUSCLE program hosted by EBI (https://www.ebi.ac.uk/Tools/msa/muscle/) for each segment [10].

Phylogenomic analysis. Alignments of sequenced samples and reference sequences were generated for all segments employing MEGA 7.0.18 [11] using default parameters for ClustalW [12] alignment. Only protein-coding sequences of each segment were retained by stripping all untranslated regions (UTRs). BEAST v1.10.4 [13] was used to create phylogenetic trees for every segment individually, and also the whole genome, with the following underlying evolutionary model and other settings: The general time-reversible (GTR) model [14] with a proportion of invariant sites and gamma-distributed rate heterogeneity across sites using four rate categories [15], an uncorrelated relaxed clock model assuming an underlying lognormal distribution [16], a coalescent piecewise-constant Bayesian Skyline model with 10 groups using a random starting tree [17], default priors and an auto-optimizing classic operator mix [18], and using tip dates. For the whole genome analysis, each segment was used as a different partition having its own site model, but sharing the same clock and tree models. For the nucleoprotein segment (NP), matrix segment (M), non-structural segment (NS), polymerase acidic subunit (PA), polymerase basic subunit 1 (PB1) and 2 (PB2) segments, the Markov-Chain-Monte-Carlo analysis was run for 500 million generations sampling every 5,000 generations for three independent replicates. For the NA and HA segments, three independent replicates were run for one billion generations sampling every 5,000 generations to accommodate for the larger phylogenetic diversity. For the whole genome, nine independent replicates were run for one billion generations sampling every 5,000 generations. The BEAGLE libraries were used for accelerating the computation [19]. Replicate trace files for individual segments and the whole genome were manually checked for arriving at the same stationary distribution using Tracer [20], and log and tree files were combined using the Logcombiner program of BEAST setting a 10% burn-in. For segments, all three segment replicates were always used. For the whole genome, only replicates with

Effective Sample Size (ESS) values for all statistics >100, i.e. six out of nine replicates, were employed to accommodate for the large computational requirements for processing these files. All combined traces always had ESS values >200 for all statistics. Maximum clade credibility (MCC) trees, i.e. the tree with the largest product of posterior clade probabilities, were afterwards generated using the TreeAnnotator program of BEAST with default settings. Generated trees were processed in ITOL (https://itol.embl.de/login.cgi) for visualization purposes [21].

A local Nextstrain instance [22], allowing light-weight phylogenomics comparison with much more genomes than is possible using the Bayesian framework implemented by BEAST, was built using the in-house sequenced samples complemented with GISAID sequences. Only GISAID genomes with whole genomes, patient sex and age information, and which were directly sequenced (i.e. no passaging in cells or eggs), were retained, resulting in 14,157 whole genomes. All sequences were aligned with CLC Genomics Workbench 20.0.2 with default parameters and UTRs were stripped on both sides. Aligned segments were then concatenated into a single sequence for all samples. Only sequences with <3 gaps and/or 'N' characters were retained. Genomes were clustered based on their sequence identity with CD-HIT 4.6.8 using different cutoffs aiming to retrieve ~3,000 genomes, which was reached with a cutoff value of 99.83%. All sequenced Belgian samples were retained irrespective of their sequence similarity (S2 Table). Some geographical metadata from GISAID was adjusted to match the countries and regions defined by default in Nextstrain. The local Nextstrain instance was constructed with Python v3.7.5 using the nextstrain augur v6.1.1 tool suite. The initial phylogenetic tree was created using the 'augur tree' command with the 'method' parameter set to 'iqtree' using IQ-TREE v1.6.12 [23]. Afterwards, the tree was refined using the 'augur

refine’ command with the ‘--timetree’ and ‘--date-confidence’ options enabled, the ‘--date-inference’ option set to ‘marginal', and the ‘--clock-filter-iqd’ option set to 4. Countries and regions for ancestral nodes were predicted using the ‘augur traits’ command with the ‘--confidence’ option enabled. Ancestral nucleotide sequences were inferred with the ‘augur ancestral’ command with the ‘--inference’ parameter set to ‘joint’. Protein sequences were translated from the nucleotide sequence with the ‘augur translate’ command using an in-house constructed GFF file containing segment coordinates in the concatenated alignment. The local instance was then exported with the ‘augur export’ command and visualized with Auspice version v2.5.2.

Reassortment detection. The reassortments were defined as described in the main manuscript.

Inference of host characteristic associations. All performed analyses to establish associations between the genomic data and the sampling information and host data are described in the main manuscript.

References

1 Thomas, I. *et al.* (2017) Virological Surveillance of Influenza in Belgium; Season 2016-2017. *Sciensano Influ. Rep.* at <https://epidemio.wiv-isp.be/ID/diseases/SiteAssets/Pages/Influenza/National Influenza Centre Belgium 2016-2017.pdf>

2 Van den Hoecke, S. *et al.* (2015) Analysis of the genetic diversity of influenza A viruses using next-generation DNA sequencing. *BMC Genomics* 16, 1–23

3 Bolger, A.M. *et al.* (2014) Trimmomatic: a flexible trimmer for Illumina sequence data. *Bioinformatics* 30, 2114–2120

4 Brister, J.R. *et al.* (2015) NCBI Viral Genomes Resource. *Nucleic Acids Res.* 43, D571–D577

5 Fu, L. *et al.* (2012) CD-HIT: accelerated for clustering the next-generation sequencing data. *Bioinformatics* 28, 3150–3152

6 Camacho, C. *et al.* (2009) BLAST+: architecture and applications. *BMC Bioinformatics* 10, 421

7 McKenna, A. *et al.* (2010) The Genome Analysis Toolkit: A MapReduce framework for analyzing next-generation DNA sequencing data. *Genome Res.* 20, 1297–1303

8 Shu, Y. and McCauley, J. (2017) GISAID: Global initiative on sharing all influenza data – from vision to reality. *Eurosurveillance* 22, 30494

9 Li, H. *et al.* (2009) The Sequence Alignment/Map format and SAMtools. *Bioinformatics* 25, 2078–2079

10 Madeira, F. *et al.* (2019) The EMBL-EBI search and sequence analysis tools APIs in 2019. *Nucleic Acids Res.* 47, W636–W641

11 Kumar, S. *et al.* (2016) MEGA7: Molecular Evolutionary Genetics Analysis Version 7.0 for Bigger Datasets. *Mol. Biol. Evol.* 33, 1870–1874

12 Larkin, M.A. *et al.* (2007) Clustal W and Clustal X version 2.0. *Bioinformatics* 23, 2947–2948

13 Suchard, M.A. *et al.* (2018) Bayesian phylogenetic and phylodynamic data integration using BEAST 1.10. *Virus Evol.* 4,

14 Tavare, S. (1986) Some probabilistic and statistical problems in the analysis of DNA sequences. *Lect. Math. life Sci.* 17,

15 Yang, Z. (1996) Among-site rate variation and its impact on phylogenetic analyses. *Trends Ecol. Evol.* 11, 367–72

16 Drummond, A.J. *et al.* (2006) Relaxed Phylogenetics and Dating with Confidence. *PLoS Biol.* 4, e88

17 Drummond, A.J. and Rambaut, A. (2007) BEAST: Bayesian evolutionary analysis by sampling trees. *BMC Evol. Biol.* 7, 214

18 Ferreira, M.A.R. and Suchard, M.A. (2008) Bayesian analysis of elapsed times in continuous-time Markov chains. *Can. J. Stat.* 36, 355–368

19 Ayres, D.L. *et al.* (2012) BEAGLE: An Application Programming Interface and High-Performance Computing Library for Statistical Phylogenetics. *Syst. Biol.* 61, 170–173

20 Rambaut, A. *et al.* (2018) Posterior Summarization in Bayesian Phylogenetics Using Tracer 1.7. *Syst. Biol.* 67, 901–904

21 Letunic, I. and Bork, P. (2019) Interactive Tree Of Life (iTOL) v4: recent updates and new developments. *Nucleic Acids Res.* 47, W256–W259

22 Hadfield, J. *et al.* (2018) Nextstrain: real-time tracking of pathogen evolution. *Bioinformatics* 34, 4121–4123

23 Chernomor, O. *et al.* (2016) Terrace Aware Data Structure for Phylogenomic Inference from Supermatrices. *Syst. Biol.* 65, 997–1008

24 Nagarajan, N. and Kingsford, C. (2011) GiRaF: robust, computational identification of influenza reassortments via graph mining. *Nucleic Acids Res.* 39, e34–e34

25 Benjamini, Y. and Hochberg, Y. Controlling the False Discovery Rate: A Practical and Powerful Approach to Multiple Testing. , *Journal of the Royal Statistical Society. Series B (Methodological)*, 57. (1995) , 289–300

R scripts used for statistical analysis

Input files: All the input files are collected in one xls file in the supplementary.

Libraries

```
library(adegenet)
library(glmnet)
library(ShortRead)
library("DECIPHER")
library(msa)
library("FactoMineR")
library("factoextra")
library(rcompanion)
library(RVAideMemoire)
library(fmsb)
library(reshape2)
library(stats)
library(dplyr)
library(ade4)
library(ape)
library("Biostrings")
library("ggplot2")
library("ggtree")
library("cluster")
library(seqinr)
library(matrixStats)
library(ggplot2)
library("lattice")
library(miscTools)
library(devtools)
library(ggpubr)
library("readxl")
library(tinytex)
```

Read in data

Groups vs Metadata

- 160 **SARI** samples
- 93 **ILI** samples

```
metadata=read_excel("8. Supplementary File - Input files statistical analysis.xlsx", sheet
= METADATA_20200102_corrected", header = TRUE, sep = ",", quote = "\"", dec = ".", stringsA
sFactors = FALSE)


# n=253

#We are excluding 4 samples, because groups and certainty of them are too uncertain
metadata <- metadata[-c(as.integer(which(metadata$Reference == "17-IG-00003")), as.integer(
which(metadata$Reference=="17-IG-00096")), as.integer(which(metadata$Reference=="17-IG-0023
7")), as.integer(which(metadata$Reference=="17-IG-00244"))),]
dim(metadata)

## [1] 249  85

metadata[metadata == "16-IG0682"] <- "16-IG-0682"
metadata[metadata == "16-IG0711"] <- "16-IG-0711"
metadata[metadata == "16-IG0712"] <- "16-IG-0712"
metadata[metadata == "16-IG0720"] <- "16-IG-0720"
```

Subset 1: Whole genome

```
WG= read_excel(“8. Supplementary File - Input files statistical analysis.xlsx”, sheet = "WG
", header = T,row.names=1, sep = ",", quote = "/", stringsAsFactors=F)

WG <- WG[-c(as.integer(which(rownames(WG) == "17-IG-00003")), as.integer(which(rownames(WG)
=="17-IG-00096")), as.integer(which(rownames(WG)=="17-IG-00237")), as.integer(which(rowname
s(WG)=="17-IG-00244"))), ,FALSE]
```

Subset 2: PB2

```
PB2= read_excel(“8. Supplementary File - Input files statistical analysis.xlsx”, sheet = "P
B2", header = T,row.names=1, sep = ",", quote = "/", stringsAsFactors=F)

PB2 <- PB2[-c(as.integer(which(rownames(PB2) == "17-IG-00003")), as.integer(which(rownames(
PB2)=="17-IG-00096")), as.integer(which(rownames(PB2)=="17-IG-00237")), as.integer(which(ro
wnames(PB2)=="17-IG-00244"))), ,FALSE]
```

Subset 3: PB1

```
PB1= read_excel(“8. Supplementary File - Input files statistical analysis.xlsx”, sheet = "P
B1", header = T,row.names=1, sep = ",", quote = "/", stringsAsFactors=F)

PB1 <- PB1[-c(as.integer(which(rownames(PB1) == "17-IG-00003")), as.integer(which(rownames(
PB1)=="17-IG-00096")), as.integer(which(rownames(PB1)=="17-IG-00237")), as.integer(which(ro
wnames(PB1)=="17-IG-00244"))), ,FALSE]
```

Subset 4: PA

```
PA= read_excel(“8. Supplementary File - Input files statistical analysis.xlsx”, sheet = "PA
", header = T,row.names=1, sep = ",", quote = "/", stringsAsFactors=F)

PA <- PA[-c(as.integer(which(rownames(PA) == "17-IG-00003")), as.integer(which(rownames(PA)
=="17-IG-00096")), as.integer(which(rownames(PA)=="17-IG-00237")), as.integer(which(rowname
s(PA)=="17-IG-00244"))), ,FALSE]
```

Subset 5: HA

```
HA= read_excel(“8. Supplementary File - Input files statistical analysis.xlsx”, sheet = "HA
", header = T,row.names=1, sep = ",", quote = "/", stringsAsFactors=F)

HA <- HA[-c(as.integer(which(rownames(HA) == "17-IG-00003")), as.integer(which(rownames(HA)
=="17-IG-00096")), as.integer(which(rownames(HA)=="17-IG-00237")), as.integer(which(rowname
s(HA)=="17-IG-00244"))), ,FALSE]
```

Subset 6: NP

```
NP= read_excel(“8. Supplementary File - Input files statistical analysis.xlsx”, sheet = "NP
", header = T,row.names=1, sep = ",", quote = "/", stringsAsFactors=F)

NP <- NP[-c(as.integer(which(rownames(NP) == "17-IG-00003")), as.integer(which(rownames(NP)
=="17-IG-00096")), as.integer(which(rownames(NP)=="17-IG-00237")), as.integer(which(rowname
s(NP)=="17-IG-00244"))), ,FALSE]
```

Subset 7: NA

```
NAs= read_excel(“8. Supplementary File - Input files statistical analysis.xlsx”, sheet = "N
A", header = T,row.names=1, sep = ",", quote = "/", stringsAsFactors=F)

NAs <- NAs[-c(as.integer(which(rownames(NAs) == "17-IG-00003")), as.integer(which(rownames(
NAs)=="17-IG-00096")), as.integer(which(rownames(NAs)=="17-IG-00237")), as.integer(which(ro
wnames(NAs)=="17-IG-00244"))), ,FALSE]
```

Subset 8: MP

```
MP= read_excel(“8. Supplementary File - Input files statistical analysis.xlsx”, sheet = "MP
", header = T,row.names=1, sep = ",", quote = "/", stringsAsFactors=F)
```

```
MP <- MP[-c(as.integer(which(rownames(MP) == "17-IG-00003")), as.integer(which(rownames(MP)
=="17-IG-00096")), as.integer(which(rownames(MP)=="17-IG-00237")), as.integer(which(rowname
s(MP)=="17-IG-00244"))), ,FALSE]
```

Subset 9: NS

```
NS= read_excel("8. Supplementary File - Input files statistical analysis.xlsx", sheet = "NS
", header = T,row.names=1, sep = ",", quote = "/", stringsAsFactors=F)

NS <- NS[-c(as.integer(which(rownames(NS) == "17-IG-00003")), as.integer(which(rownames(NS)
=="17-IG-00096")), as.integer(which(rownames(NS)=="17-IG-00237")), as.integer(which(rowname
s(NS)=="17-IG-00244"))), ,FALSE]
```

Reassormtents vs Metadata

```
metadataReassort= read_excel("8. Supplementary File - Input files statistical analysis.xlsx
", sheet = METADATA_20200102_corrected", header = TRUE, sep = ",", quote = "\"", dec = ".",
stringsAsFactors = FALSE)
```

Subset 10: Reassortments

```
metadataReassort= read_excel("8. Supplementary File - Input files statistical analysis.xlsx
", sheet = METADATA_20200102_corrected", header = TRUE, sep = ",", quote = "\"", dec = ".",
stringsAsFactors = FALSE)

metadataReassort[metadataReassort == "16-IG0682"] <- "16-IG-0682"
metadataReassort[metadataReassort == "16-IG0711"] <- "16-IG-0711"
metadataReassort[metadataReassort == "16-IG0712"] <- "16-IG-0712"
metadataReassort[metadataReassort == "16-IG0720"] <- "16-IG-0720"

Reassort= read_excel("8. Supplementary File - Input files statistical analysis.xlsx", sheet
= "ReassortmentsYN_Combined0.95", header = T,row.names=1, sep = ",", quote = "/", stringsAs
Factors=F)
```

Fisher tests

WG

```
metadata<- metadata[match(rownames(WG), as.character(metadata$Reference)),]

#ILI=0; SARI=1
#Male=0; Female=1
#No=0; Yes=1
#Alive=0; Deceased=1
#Beginning=0; Middle=1; End=2
#<15=0; 15-59=1; >=60=2
#Mild=0; Moderate=1; Severe=2

varlist2 <- c("metadata$Surveillance", "metadata$Sex", "metadata$ca_vacci", "metadata$ca_st
atus", "metadata$ca_antibio", "metadata$asthma_respi", "metadata$cardio", "metadata$obesity
", "metadata$diabete", "metadata$renal", "metadata$immuno", "metadata$neuro", "metadata$hep
ato", "metadata$ca_icuhosp")

varlist3 <- c("metadata$Period", "metadata$Age.cat", "metadata$MildModerateSevere")

varlist <- c("Surveillance", "Sex", "ca_vacci", "ca_status", "ca_antibio", "asthma_respi",
"cardio", "obesity", "diabete", "renal", "immuno", "neuro", "hepato", "ca_icuhosp","Period"
, "Age.cat", "MildModerateSevere")


WG.fun2 <- (paste("apply(WG,2,function(e) fisher.test(table(factor(e,levels=c(0, 1)), facto
r(", varlist2,", levels=c(0, 1))))$p.value)", sep=""))
```

```
WG.fun3 <- (paste("apply(WG,2,function(e) fisher.test(table(factor(e,levels=c(0, 1)), factor(", varlist3,", levels=c(0, 1, 2))))$p.value)", sep=""))
WG.fun <- c(WG.fun2,WG.fun3)
WG.fisher <- lapply(WG.fun, function(x) {eval(parse(text = x))})
names(WG.fisher) <- varlist
#WG.fisher

WG.fun.cont2 <- (paste("apply(WG, 2, function(e)table(factor(e, levels=c(0,1)), factor(", varlist2,", levels=c(0, 1))))[,c(names(WG))]", sep=""))
WG.fun.cont3 <- (paste("apply(WG, 2, function(e)table(factor(e, levels=c(0,1)), factor(", varlist3,", levels=c(0, 1, 2))))[,c(names(WG))]", sep=""))
WG.fun.cont<-c(WG.fun.cont2,WG.fun.cont3)
WG.fisher.cont <- lapply(WG.fun.cont, function(x) {eval(parse(text = x))})
names(WG.fisher.cont) <- varlist
WG.fisher.cont2 <- (melt(WG.fisher.cont))
WG.fisher.cont2$Name <- paste(WG.fisher.cont2$Var2,"_",WG.fisher.cont2$L1,"_",WG.fisher.cont2$Var1, sep="")
rownames(WG.fisher.cont2)<-WG.fisher.cont2$Name
WG.fisher.cont2$Var1<-NULL
WG.fisher.cont2$Var2<-NULL
WG.fisher.cont2$L1<-NULL
WG.fisher.cont2$Name<-NULL
#WG.fisher.cont2


WG.fun.fdr2 <- noquote(paste("p.adjust(apply(WG,2,function(e) fisher.test(table(factor(e,levels=c(0, 1)), factor(", varlist2,", levels=c(0, 1))))$p.value),method=\"fdr\")", sep=""))
WG.fun.fdr3 <- noquote(paste("p.adjust(apply(WG,2,function(e) fisher.test(table(factor(e,levels=c(0, 1)), factor(", varlist3,", levels=c(0, 1, 2))))$p.value),method=\"fdr\")", sep=""))
WG.fun.fdr <- c(WG.fun.fdr2,WG.fun.fdr3)
WG.fisher.fdr <- lapply(WG.fun.fdr, function(x) {eval(parse(text = x))})
names(WG.fisher.fdr) <- varlist
#WG.fisher.fdr

write.csv(WG.fisher, file.path("Results/WG.fisher.csv"))

write.csv(WG.fisher.cont2, file.path("Results/WG.fisher.cont.csv"))

write.csv(WG.fisher.fdr, file.path("Results/WG.fisher.fdr.csv"))
```

PB2

```
metadata<- metadata[match(rownames(PB2), as.character(metadata$Reference)),]

#ILI=0; SARI=1
#Male=0; Female=1
#No=0; Yes=1
#Alive=0; Deceased=1
#Beginning=0; Middle=1; End=2
#<15=0; 15-59=1; >=60=2
#Mild=0; Moderate=1; Severe=2

varlist2 <- c("metadata$Surveillance", "metadata$Sex", "metadata$ca_vacci", "metadata$ca_status", "metadata$ca_antibio", "metadata$asthma_respi", "metadata$cardio", "metadata$obesity", "metadata$diabete", "metadata$renal", "metadata$immuno", "metadata$neuro", "metadata$hepato", "metadata$ca_icuhosp")

varlist3 <- c("metadata$Period", "metadata$Age.cat", "metadata$MildModerateSevere")

varlist <- c("Surveillance", "Sex", "ca_vacci", "ca_status", "ca_antibio", "asthma_respi", "cardio", "obesity", "diabete", "renal", "immuno", "neuro", "hepato", "ca_icuhosp","Period", "Age.cat", "MildModerateSevere")
```

```
PB2.fun2 <- (paste("apply(PB2,2,function(e) fisher.test(table(factor(e,levels=c(0, 1)), factor(", varlist2,", levels=c(0, 1))))$p.value)", sep=""))
PB2.fun3 <- (paste("apply(PB2,2,function(e) fisher.test(table(factor(e,levels=c(0, 1)), factor(", varlist3,", levels=c(0, 1, 2))))$p.value)", sep=""))
PB2.fun <- c(PB2.fun2,PB2.fun3)
PB2.fisher <- lapply(PB2.fun, function(x) {eval(parse(text = x))})
names(PB2.fisher) <- varlist
#PB2.fisher

PB2.fun.cont2 <- (paste("apply(PB2, 2, function(e)table(factor(e, levels=c(0,1)), factor(", varlist2,", levels=c(0, 1))))[,c(names(PB2))]", sep=""))
PB2.fun.cont3 <- (paste("apply(PB2, 2, function(e)table(factor(e, levels=c(0,1)), factor(", varlist3,", levels=c(0, 1, 2))))[,c(names(PB2))]", sep=""))
PB2.fun.cont<-c(PB2.fun.cont2,PB2.fun.cont3)
PB2.fisher.cont <- lapply(PB2.fun.cont, function(x) {eval(parse(text = x))})
names(PB2.fisher.cont) <- varlist
PB2.fisher.cont2 <- (melt(PB2.fisher.cont))
PB2.fisher.cont2$Name <- paste(PB2.fisher.cont2$Var2,"_",PB2.fisher.cont2$L1,"_",PB2.fisher.cont2$Var1, sep="")
rownames(PB2.fisher.cont2)<-PB2.fisher.cont2$Name
PB2.fisher.cont2$Var1<-NULL
PB2.fisher.cont2$Var2<-NULL
PB2.fisher.cont2$L1<-NULL
PB2.fisher.cont2$Name<-NULL
#PB2.fisher.cont2


PB2.fun.fdr2 <- noquote(paste("p.adjust(apply(PB2,2,function(e) fisher.test(table(factor(e, levels=c(0, 1)), factor(", varlist2,", levels=c(0, 1))))$p.value),method=\"fdr\")", sep="")
)
PB2.fun.fdr3 <- noquote(paste("p.adjust(apply(PB2,2,function(e) fisher.test(table(factor(e, levels=c(0, 1)), factor(", varlist3,", levels=c(0, 1, 2))))$p.value),method=\"fdr\")", sep=
""))
PB2.fun.fdr <- c(PB2.fun.fdr2,PB2.fun.fdr3)
PB2.fisher.fdr <- lapply(PB2.fun.fdr, function(x) {eval(parse(text = x))})
names(PB2.fisher.fdr) <- varlist
#PB2.fisher.fdr

write.csv(PB2.fisher, file.path("Results/PB2.fisher.csv"))

write.csv(PB2.fisher.cont2, file.path("Results/PB2.fisher.cont.csv"))

write.csv(PB2.fisher.fdr, file.path("Results/PB2.fisher.fdr.csv"))
```

PB1

```
metadata<- metadata[match(rownames(PB1), as.character(metadata$Reference)),]

#ILI=0; SARI=1
#Male=0; Female=1
#No=0; Yes=1
#Alive=0; Deceased=1
#Beginning=0; Middle=1; End=2
#<15=0; 15-59=1; >=60=2
#Mild=0; Moderate=1; Severe=2

varlist2 <- c("metadata$Surveillance", "metadata$Sex", "metadata$ca_vacci", "metadata$ca_status", "metadata$ca_antibio", "metadata$asthma_respi", "metadata$cardio", "metadata$obesity", "metadata$diabete", "metadata$renal", "metadata$immuno", "metadata$neuro", "metadata$hepato", "metadata$ca_icuhosp")
```

```
varlist3   <-   c("metadata$Period",   "metadata$Age.cat",   "metadata$MildModerateSevere")

varlist <- c("Surveillance", "Sex", "ca_vacci", "ca_status", "ca_antibio", "asthma_respi", "cardio", "obesity", "diabete", "renal", "immuno", "neuro", "hepato", "ca_icuhosp","Period", "Age.cat", "MildModerateSevere")


PB1.fun2 <- (paste("apply(PB1,2,function(e) fisher.test(table(factor(e,levels=c(0, 1)), factor(", varlist2,", levels=c(0, 1))))$p.value)", sep=""))
PB1.fun3 <- (paste("apply(PB1,2,function(e) fisher.test(table(factor(e,levels=c(0, 1)), factor(", varlist3,", levels=c(0, 1, 2))))$p.value)", sep=""))
PB1.fun <- c(PB1.fun2,PB1.fun3)
PB1.fisher <- lapply(PB1.fun, function(x) {eval(parse(text = x))})
names(PB1.fisher) <- varlist
#PB1.fisher

PB1.fun.cont2 <- (paste("apply(PB1, 2, function(e)table(factor(e, levels=c(0,1)), factor(", varlist2,", levels=c(0, 1))))[,c(names(PB1))]", sep=""))
PB1.fun.cont3 <- (paste("apply(PB1, 2, function(e)table(factor(e, levels=c(0,1)), factor(", varlist3,", levels=c(0, 1, 2))))[,c(names(PB1))]", sep=""))
PB1.fun.cont<-c(PB1.fun.cont2,PB1.fun.cont3)
PB1.fisher.cont <- lapply(PB1.fun.cont, function(x) {eval(parse(text = x))})
names(PB1.fisher.cont) <- varlist
PB1.fisher.cont2 <- (melt(PB1.fisher.cont))
PB1.fisher.cont2$Name <- paste(PB1.fisher.cont2$Var2,"_",PB1.fisher.cont2$L1,"_",PB1.fisher.cont2$Var1, sep="")
rownames(PB1.fisher.cont2)<-PB1.fisher.cont2$Name
PB1.fisher.cont2$Var1<-NULL
PB1.fisher.cont2$Var2<-NULL
PB1.fisher.cont2$L1<-NULL
PB1.fisher.cont2$Name<-NULL
#PB1.fisher.cont2


PB1.fun.fdr2 <- noquote(paste("p.adjust(apply(PB1,2,function(e) fisher.test(table(factor(e, levels=c(0, 1)), factor(", varlist2,", levels=c(0, 1))))$p.value),method=\"fdr\")", sep="")
)
PB1.fun.fdr3 <- noquote(paste("p.adjust(apply(PB1,2,function(e) fisher.test(table(factor(e, levels=c(0, 1)), factor(", varlist3,", levels=c(0, 1, 2))))$p.value),method=\"fdr\")", sep=
""))
PB1.fun.fdr <- c(PB1.fun.fdr2,PB1.fun.fdr3)
PB1.fisher.fdr <- lapply(PB1.fun.fdr, function(x) {eval(parse(text = x))})
names(PB1.fisher.fdr) <- varlist
#PB1.fisher.fdr

write.csv(PB1.fisher, file.path("Results/PB1.fisher.csv"))

write.csv(PB1.fisher.cont2, file.path("Results/PB1.fisher.cont.csv"))

write.csv(PB1.fisher.fdr, file.path("Results/PB1.fisher.fdr.csv"))
```

PA

```
metadata<- metadata[match(rownames(PA), as.character(metadata$Reference)),]

#ILI=0; SARI=1
#Male=0; Female=1
#No=0; Yes=1
#Alive=0; Deceased=1
#Beginning=0; Middle=1; End=2
#<15=0; 15-59=1; >=60=2
#Mild=0; Moderate=1; Severe=2
```

```r
varlist2 <- c("metadata$Surveillance", "metadata$Sex", "metadata$ca_vacci", "metadata$ca_status", "metadata$ca_antibio", "metadata$asthma_respi", "metadata$cardio", "metadata$obesity", "metadata$diabete", "metadata$renal", "metadata$immuno", "metadata$neuro", "metadata$hepato", "metadata$ca_icuhosp")

varlist3 <- c("metadata$Period", "metadata$Age.cat", "metadata$MildModerateSevere")

varlist <- c("Surveillance", "Sex", "ca_vacci", "ca_status", "ca_antibio", "asthma_respi", "cardio", "obesity", "diabete", "renal", "immuno", "neuro", "hepato", "ca_icuhosp","Period", "Age.cat", "MildModerateSevere")


PA.fun2 <- (paste("apply(PA,2,function(e) fisher.test(table(factor(e,levels=c(0, 1)), factor(", varlist2,", levels=c(0, 1))))$p.value)", sep=""))
PA.fun3 <- (paste("apply(PA,2,function(e) fisher.test(table(factor(e,levels=c(0, 1)), factor(", varlist3,", levels=c(0, 1, 2))))$p.value)", sep=""))
PA.fun <- c(PA.fun2,PA.fun3)
PA.fisher <- lapply(PA.fun, function(x) {eval(parse(text = x))})
names(PA.fisher) <- varlist
#PA.fisher

PA.fun.cont2 <- (paste("apply(PA, 2, function(e)table(factor(e, levels=c(0,1)), factor(", varlist2,", levels=c(0, 1))))[,c(names(PA))]", sep=""))
PA.fun.cont3 <- (paste("apply(PA, 2, function(e)table(factor(e, levels=c(0,1)), factor(", varlist3,", levels=c(0, 1, 2))))[,c(names(PA))]", sep=""))
PA.fun.cont<-c(PA.fun.cont2,PA.fun.cont3)
PA.fisher.cont <- lapply(PA.fun.cont, function(x) {eval(parse(text = x))})
names(PA.fisher.cont) <- varlist
PA.fisher.cont2 <- (melt(PA.fisher.cont))
PA.fisher.cont2$Name <- paste(PA.fisher.cont2$Var2,"_",PA.fisher.cont2$L1,"_",PA.fisher.cont2$Var1, sep="")
rownames(PA.fisher.cont2)<-PA.fisher.cont2$Name
PA.fisher.cont2$Var1<-NULL
PA.fisher.cont2$Var2<-NULL
PA.fisher.cont2$L1<-NULL
PA.fisher.cont2$Name<-NULL
#PA.fisher.cont2


PA.fun.fdr2 <- noquote(paste("p.adjust(apply(PA,2,function(e) fisher.test(table(factor(e,levels=c(0, 1)), factor(", varlist2,", levels=c(0, 1))))$p.value),method=\"fdr\")", sep=""))
PA.fun.fdr3 <- noquote(paste("p.adjust(apply(PA,2,function(e) fisher.test(table(factor(e,levels=c(0, 1)), factor(", varlist3,", levels=c(0, 1, 2))))$p.value),method=\"fdr\")", sep=""))
PA.fun.fdr <- c(PA.fun.fdr2,PA.fun.fdr3)
PA.fisher.fdr <- lapply(PA.fun.fdr, function(x) {eval(parse(text = x))})
names(PA.fisher.fdr) <- varlist
#PA.fisher.fdr

write.csv(PA.fisher, file.path("Results/PA.fisher.csv"))

write.csv(PA.fisher.cont2, file.path("Results/PA.fisher.cont.csv"))

write.csv(PA.fisher.fdr, file.path("Results/PA.fisher.fdr.csv"))
```

HA

```r
metadata<- metadata[match(rownames(HA), as.character(metadata$Reference)),]

#ILI=0; SARI=1
#Male=0; Female=1
#No=0; Yes=1
#Alive=0; Deceased=1
```

```
#Beginning=0;                          Middle=1;                          End=2
#<15=0;                                15-59=1;                           >=60=2
#Mild=0;                               Moderate=1;                        Severe=2

varlist2 <- c("metadata$Surveillance", "metadata$Sex", "metadata$ca_vacci", "metadata$ca_st
atus", "metadata$ca_antibio", "metadata$asthma_respi", "metadata$cardio", "metadata$obesity
", "metadata$diabete", "metadata$renal", "metadata$immuno", "metadata$neuro", "metadata$hep
ato",                                                              "metadata$ca_icuhosp")

varlist3   <-   c("metadata$Period",    "metadata$Age.cat",    "metadata$MildModerateSevere")

varlist <- c("Surveillance", "Sex", "ca_vacci", "ca_status", "ca_antibio", "asthma_respi",
"cardio", "obesity", "diabete", "renal", "immuno", "neuro", "hepato", "ca_icuhosp","Period"
,                          "Age.cat",                              "MildModerateSevere")


HA.fun2 <- (paste("apply(HA,2,function(e) fisher.test(table(factor(e,levels=c(0, 1)), facto
r(",        varlist2,",          levels=c(0,          1))))$p.value)",          sep=""))
HA.fun3 <- (paste("apply(HA,2,function(e) fisher.test(table(factor(e,levels=c(0, 1)), facto
r(",       varlist3,",        levels=c(0,        1,        2))))$p.value)",        sep=""))
HA.fun                                   <-                               c(HA.fun2,HA.fun3)
HA.fisher      <-      lapply(HA.fun,      function(x)      {eval(parse(text      =      x))})
names(HA.fisher)                                <-                                   varlist
#HA.fisher

HA.fun.cont2 <- (paste("apply(HA, 2, function(e)table(factor(e, levels=c(0,1)), factor(", v
arlist2,",            levels=c(0,             1))))[,c(names(HA))]",             sep=""))
HA.fun.cont3 <- (paste("apply(HA, 2, function(e)table(factor(e, levels=c(0,1)), factor(", v
arlist3,",          levels=c(0,          1,          2))))[,c(names(HA))]",          sep=""))
HA.fun.cont<-c(HA.fun.cont2,HA.fun.cont3)
HA.fisher.cont    <-    lapply(HA.fun.cont,    function(x)    {eval(parse(text    =    x))})
names(HA.fisher.cont)                             <-                                 varlist
HA.fisher.cont2                          <-                           (melt(HA.fisher.cont))
HA.fisher.cont2$Name <- paste(HA.fisher.cont2$Var2,"_",HA.fisher.cont2$L1,"_",HA.fisher.con
t2$Var1,                                                                             sep="")
rownames(HA.fisher.cont2)<-HA.fisher.cont2$Name
HA.fisher.cont2$Var1<-NULL
HA.fisher.cont2$Var2<-NULL
HA.fisher.cont2$L1<-NULL
HA.fisher.cont2$Name<-NULL
#HA.fisher.cont2


HA.fun.fdr2 <- noquote(paste("p.adjust(apply(HA,2,function(e) fisher.test(table(factor(e,le
vels=c(0, 1)), factor(", varlist2,", levels=c(0, 1))))$p.value),method=\"fdr\")", sep=""))
HA.fun.fdr3 <- noquote(paste("p.adjust(apply(HA,2,function(e) fisher.test(table(factor(e,le
vels=c(0, 1)), factor(", varlist3,", levels=c(0, 1, 2))))$p.value),method=\"fdr\")", sep=""
))
HA.fun.fdr                           <-                           c(HA.fun.fdr2,HA.fun.fdr3)
HA.fisher.fdr     <-     lapply(HA.fun.fdr,     function(x)     {eval(parse(text     =     x))})
names(HA.fisher.fdr)                              <-                                 varlist
#HA.fisher.fdr

write.csv(HA.fisher,                                   file.path("Results/HA.fisher.csv"))

write.csv(HA.fisher.cont2,                        file.path("Results/HA.fisher.cont.csv"))

write.csv(HA.fisher.fdr, file.path("Results/HA.fisher.fdr.csv"))
```

NP

```
metadata<-         metadata[match(rownames(NP),         as.character(metadata$Reference)),]
```

```
#ILI=0;                                        SARI=1
#Male=0;                                       Female=1
#No=0;                                         Yes=1
#Alive=0;                                      Deceased=1
#Beginning=0;             Middle=1;            End=2
#<15=0;                   15-59=1;             >=60=2
#Mild=0;                  Moderate=1;          Severe=2

varlist2 <- c("metadata$Surveillance", "metadata$Sex", "metadata$ca_vacci", "metadata$ca_status", "metadata$ca_antibio", "metadata$asthma_respi", "metadata$cardio", "metadata$obesity", "metadata$diabete", "metadata$renal", "metadata$immuno", "metadata$neuro", "metadata$hepato", "metadata$ca_icuhosp")

varlist3 <- c("metadata$Period", "metadata$Age.cat", "metadata$MildModerateSevere")

varlist <- c("Surveillance", "Sex", "ca_vacci", "ca_status", "ca_antibio", "asthma_respi", "cardio", "obesity", "diabete", "renal", "immuno", "neuro", "hepato", "ca_icuhosp","Period", "Age.cat", "MildModerateSevere")


NP.fun2 <- (paste("apply(NP,2,function(e) fisher.test(table(factor(e,levels=c(0, 1)), factor(", varlist2,", levels=c(0, 1))))$p.value)", sep=""))
NP.fun3 <- (paste("apply(NP,2,function(e) fisher.test(table(factor(e,levels=c(0, 1)), factor(", varlist3,", levels=c(0, 1, 2))))$p.value)", sep=""))
NP.fun <- c(NP.fun2,NP.fun3)
NP.fisher <- lapply(NP.fun, function(x) {eval(parse(text = x))})
names(NP.fisher) <- varlist
#NP.fisher

NP.fun.cont2 <- (paste("apply(NP, 2, function(e)table(factor(e, levels=c(0,1)), factor(", varlist2,", levels=c(0, 1))))[,c(names(NP))]", sep=""))
NP.fun.cont3 <- (paste("apply(NP, 2, function(e)table(factor(e, levels=c(0,1)), factor(", varlist3,", levels=c(0, 1, 2))))[,c(names(NP))]", sep=""))
NP.fun.cont<-c(NP.fun.cont2,NP.fun.cont3)
NP.fisher.cont <- lapply(NP.fun.cont, function(x) {eval(parse(text = x))})
names(NP.fisher.cont) <- varlist
NP.fisher.cont2 <- (melt(NP.fisher.cont))
NP.fisher.cont2$Name <- paste(NP.fisher.cont2$Var2,"_",NP.fisher.cont2$L1,"_",NP.fisher.cont2$Var1, sep="")
rownames(NP.fisher.cont2)<-NP.fisher.cont2$Name
NP.fisher.cont2$Var1<-NULL
NP.fisher.cont2$Var2<-NULL
NP.fisher.cont2$L1<-NULL
NP.fisher.cont2$Name<-NULL
#NP.fisher.cont2


NP.fun.fdr2 <- noquote(paste("p.adjust(apply(NP,2,function(e) fisher.test(table(factor(e,levels=c(0, 1)), factor(", varlist2,", levels=c(0, 1))))$p.value),method=\"fdr\")", sep=""))
NP.fun.fdr3 <- noquote(paste("p.adjust(apply(NP,2,function(e) fisher.test(table(factor(e,levels=c(0, 1)), factor(", varlist3,", levels=c(0, 1, 2))))$p.value),method=\"fdr\")", sep=""))
NP.fun.fdr <- c(NP.fun.fdr2,NP.fun.fdr3)
NP.fisher.fdr <- lapply(NP.fun.fdr, function(x) {eval(parse(text = x))})
names(NP.fisher.fdr) <- varlist
#NP.fisher.fdr

write.csv(NP.fisher, file.path("Results/NP.fisher.csv"))

write.csv(NP.fisher.cont2, file.path("Results/NP.fisher.cont.csv"))

write.csv(NP.fisher.fdr, file.path("Results/NP.fisher.fdr.csv"))
```

NA

```
metadata<- metadata[match(rownames(NAs), as.character(metadata$Reference)),]

#ILI=0; SARI=1
#Male=0; Female=1
#No=0; Yes=1
#Alive=0; Deceased=1
#Beginning=0; Middle=1; End=2
#<15=0; 15-59=1; >=60=2
#Mild=0; Moderate=1; Severe=2

varlist2 <- c("metadata$Surveillance", "metadata$Sex", "metadata$ca_vacci", "metadata$ca_status", "metadata$ca_antibio", "metadata$asthma_respi", "metadata$cardio", "metadata$obesity", "metadata$diabete", "metadata$renal", "metadata$immuno", "metadata$neuro", "metadata$hepato", "metadata$ca_icuhosp")

varlist3 <- c("metadata$Period", "metadata$Age.cat", "metadata$MildModerateSevere")

varlist <- c("Surveillance", "Sex", "ca_vacci", "ca_status", "ca_antibio", "asthma_respi", "cardio", "obesity", "diabete", "renal", "immuno", "neuro", "hepato", "ca_icuhosp","Period", "Age.cat", "MildModerateSevere")


NAs.fun2 <- (paste("apply(NAs,2,function(e) fisher.test(table(factor(e,levels=c(0, 1)), factor(", varlist2,", levels=c(0, 1))))$p.value)", sep=""))
NAs.fun3 <- (paste("apply(NAs,2,function(e) fisher.test(table(factor(e,levels=c(0, 1)), factor(", varlist3,", levels=c(0, 1, 2))))$p.value)", sep=""))
NAs.fun <- c(NAs.fun2,NAs.fun3)
NAs.fisher <- lapply(NAs.fun, function(x) {eval(parse(text = x))})
names(NAs.fisher) <- varlist
#NAs.fisher

NAs.fun.cont2 <- (paste("apply(NAs, 2, function(e)table(factor(e, levels=c(0,1)), factor(", varlist2,", levels=c(0, 1))))[,c(names(NAs))]", sep=""))
NAs.fun.cont3 <- (paste("apply(NAs, 2, function(e)table(factor(e, levels=c(0,1)), factor(", varlist3,", levels=c(0, 1, 2))))[,c(names(NAs))]", sep=""))
NAs.fun.cont<-c(NAs.fun.cont2,NAs.fun.cont3)
NAs.fisher.cont <- lapply(NAs.fun.cont, function(x) {eval(parse(text = x))})
names(NAs.fisher.cont) <- varlist
NAs.fisher.cont2 <- (melt(NAs.fisher.cont))
NAs.fisher.cont2$Name <- paste(NAs.fisher.cont2$Var2,"_",NAs.fisher.cont2$L1,"_",NAs.fisher.cont2$Var1, sep="")
rownames(NAs.fisher.cont2)<-NAs.fisher.cont2$Name
NAs.fisher.cont2$Var1<-NULL
NAs.fisher.cont2$Var2<-NULL
NAs.fisher.cont2$L1<-NULL
NAs.fisher.cont2$Name<-NULL
#NAs.fisher.cont2


NAs.fun.fdr2 <- noquote(paste("p.adjust(apply(NAs,2,function(e) fisher.test(table(factor(e, levels=c(0, 1)), factor(", varlist2,", levels=c(0, 1))))$p.value),method=\"fdr\")", sep=""))
NAs.fun.fdr3 <- noquote(paste("p.adjust(apply(NAs,2,function(e) fisher.test(table(factor(e, levels=c(0, 1)), factor(", varlist3,", levels=c(0, 1, 2))))$p.value),method=\"fdr\")", sep=""))
NAs.fun.fdr <- c(NAs.fun.fdr2,NAs.fun.fdr3)
NAs.fisher.fdr <- lapply(NAs.fun.fdr, function(x) {eval(parse(text = x))})
names(NAs.fisher.fdr) <- varlist
#NAs.fisher.fdr

write.csv(NAs.fisher, file.path("Results/NAs.fisher.csv"))
```

```
write.csv(NAs.fisher.cont2,                              file.path("Results/NAs.fisher.cont.csv"))

write.csv(NAs.fisher.fdr, file.path("Results/NAs.fisher.fdr.csv"))
```

MP

```
metadata<-         metadata[match(rownames(MP),         as.character(metadata$Reference)),]

#ILI=0;                                                                                  SARI=1
#Male=0;                                                                               Female=1
#No=0;                                                                                     Yes=1
#Alive=0;                                                                            Deceased=1
#Beginning=0;                              Middle=1;                                     End=2
#<15=0;                                   15-59=1;                                     >=60=2
#Mild=0;                                Moderate=1;                                  Severe=2

varlist2 <- c("metadata$Surveillance", "metadata$Sex", "metadata$ca_vacci", "metadata$ca_st
atus", "metadata$ca_antibio", "metadata$asthma_respi", "metadata$cardio", "metadata$obesity
", "metadata$diabete", "metadata$renal", "metadata$immuno", "metadata$neuro", "metadata$hep
ato",                                                               "metadata$ca_icuhosp")

varlist3   <-   c("metadata$Period",    "metadata$Age.cat",    "metadata$MildModerateSevere")

varlist <- c("Surveillance", "Sex", "ca_vacci", "ca_status", "ca_antibio", "asthma_respi",
"cardio", "obesity", "diabete", "renal", "immuno", "neuro", "hepato", "ca_icuhosp","Period"
,                             "Age.cat",                                "MildModerateSevere")


MP.fun2 <- (paste("apply(MP,2,function(e) fisher.test(table(factor(e,levels=c(0, 1)), facto
r(",          varlist2,",          levels=c(0,          1))))$p.value)",          sep=""))
MP.fun3 <- (paste("apply(MP,2,function(e) fisher.test(table(factor(e,levels=c(0, 1)), facto
r(",        varlist3,",        levels=c(0,        1,        2))))$p.value)",      sep=""))
MP.fun                                      <-                                c(MP.fun2,MP.fun3)
MP.fisher      <-      lapply(MP.fun,      function(x)      {eval(parse(text      =      x))})
names(MP.fisher)                                      <-                                varlist
#MP.fisher

MP.fun.cont2 <- (paste("apply(MP, 2, function(e)table(factor(e, levels=c(0,1)), factor(", v
arlist2,",             levels=c(0,              1))))[,c(names(MP))]",             sep=""))
MP.fun.cont3 <- (paste("apply(MP, 2, function(e)table(factor(e, levels=c(0,1)), factor(", v
arlist3,",          levels=c(0,          1,          2))))[,c(names(MP))]",          sep=""))
MP.fun.cont<-c(MP.fun.cont2,MP.fun.cont3)
MP.fisher.cont    <-    lapply(MP.fun.cont,    function(x)    {eval(parse(text    =    x))})
names(MP.fisher.cont)                                   <-                              varlist
MP.fisher.cont2                             <-                           (melt(MP.fisher.cont))
MP.fisher.cont2$Name <- paste(MP.fisher.cont2$Var2,"_",MP.fisher.cont2$L1,"_",MP.fisher.con
t2$Var1,                                                                                sep="")
rownames(MP.fisher.cont2)<-MP.fisher.cont2$Name
MP.fisher.cont2$Var1<-NULL
MP.fisher.cont2$Var2<-NULL
MP.fisher.cont2$L1<-NULL
MP.fisher.cont2$Name<-NULL
#MP.fisher.cont2


MP.fun.fdr2 <- noquote(paste("p.adjust(apply(MP,2,function(e) fisher.test(table(factor(e,le
vels=c(0, 1)), factor(", varlist2,", levels=c(0, 1))))$p.value),method=\"fdr\")", sep=""))
MP.fun.fdr3 <- noquote(paste("p.adjust(apply(MP,2,function(e) fisher.test(table(factor(e,le
vels=c(0, 1)), factor(", varlist3,", levels=c(0, 1, 2))))$p.value),method=\"fdr\")", sep=""
))
MP.fun.fdr                              <-                           c(MP.fun.fdr2,MP.fun.fdr3)
MP.fisher.fdr     <-     lapply(MP.fun.fdr,     function(x)     {eval(parse(text     =     x))})
```

```
names(MP.fisher.fdr) <- varlist
#MP.fisher.fdr

write.csv(MP.fisher, file.path("Results/MP.fisher.csv"))

write.csv(MP.fisher.cont2, file.path("Results/MP.fisher.cont.csv"))

write.csv(MP.fisher.fdr, file.path("Results/MP.fisher.fdr.csv"))
```

NS

```
metadata<- metadata[match(rownames(NS), as.character(metadata$Reference)),]

#ILI=0; SARI=1
#Male=0; Female=1
#No=0; Yes=1
#Alive=0; Deceased=1
#Beginning=0; Middle=1; End=2
#<15=0; 15-59=1; >=60=2
#Mild=0; Moderate=1; Severe=2

varlist2 <- c("metadata$Surveillance", "metadata$Sex", "metadata$ca_vacci", "metadata$ca_status", "metadata$ca_antibio", "metadata$asthma_respi", "metadata$cardio", "metadata$obesity", "metadata$diabete", "metadata$renal", "metadata$immuno", "metadata$neuro", "metadata$hepato", "metadata$ca_icuhosp")

varlist3 <- c("metadata$Period", "metadata$Age.cat", "metadata$MildModerateSevere")

varlist <- c("Surveillance", "Sex", "ca_vacci", "ca_status", "ca_antibio", "asthma_respi", "cardio", "obesity", "diabete", "renal", "immuno", "neuro", "hepato", "ca_icuhosp","Period", "Age.cat", "MildModerateSevere")


NS.fun2 <- (paste("apply(NS,2,function(e) fisher.test(table(factor(e,levels=c(0, 1)), factor(", varlist2,", levels=c(0, 1))))$p.value)", sep=""))
NS.fun3 <- (paste("apply(NS,2,function(e) fisher.test(table(factor(e,levels=c(0, 1)), factor(", varlist3,", levels=c(0, 1, 2))))$p.value)", sep=""))
NS.fun <- c(NS.fun2,NS.fun3)
NS.fisher <- lapply(NS.fun, function(x) {eval(parse(text = x))})
names(NS.fisher) <- varlist
#NS.fisher

NS.fun.cont2 <- (paste("apply(NS, 2, function(e)table(factor(e, levels=c(0,1)), factor(", varlist2,", levels=c(0, 1))))[,c(names(NS))]", sep=""))
NS.fun.cont3 <- (paste("apply(NS, 2, function(e)table(factor(e, levels=c(0,1)), factor(", varlist3,", levels=c(0, 1, 2))))[,c(names(NS))]", sep=""))
NS.fun.cont<-c(NS.fun.cont2,NS.fun.cont3)
NS.fisher.cont <- lapply(NS.fun.cont, function(x) {eval(parse(text = x))})
names(NS.fisher.cont) <- varlist
NS.fisher.cont2 <- (melt(NS.fisher.cont))
NS.fisher.cont2$Name <- paste(NS.fisher.cont2$Var2,"_",NS.fisher.cont2$L1,"_",NS.fisher.cont2$Var1, sep="")
rownames(NS.fisher.cont2)<-NS.fisher.cont2$Name
NS.fisher.cont2$Var1<-NULL
NS.fisher.cont2$Var2<-NULL
NS.fisher.cont2$L1<-NULL
NS.fisher.cont2$Name<-NULL
#NS.fisher.cont2


NS.fun.fdr2 <- noquote(paste("p.adjust(apply(NS,2,function(e) fisher.test(table(factor(e,levels=c(0, 1)), factor(", varlist2,", levels=c(0, 1))))$p.value),method=\"fdr\")", sep=""))
NS.fun.fdr3 <- noquote(paste("p.adjust(apply(NS,2,function(e) fisher.test(table(factor(e,le
```

```
vels=c(0, 1)), factor(", varlist3,", levels=c(0, 1, 2))))$p.value),method=\"fdr\")", sep=""
))
NS.fun.fdr <- c(NS.fun.fdr2,NS.fun.fdr3)
NS.fisher.fdr <- lapply(NS.fun.fdr, function(x) {eval(parse(text = x))})
names(NS.fisher.fdr) <- varlist
#NS.fisher.fdr

write.csv(NS.fisher, file.path("Results/NS.fisher.csv"))

write.csv(NS.fisher.cont2, file.path("Results/NS.fisher.cont.csv"))

write.csv(NS.fisher.fdr, file.path("Results/NS.fisher.fdr.csv"))
```

Reassortments

```
metadataReassort<- metadataReassort[match(rownames(Reassort), as.character(metadataReassort
$Reference)),]

#ILI=0; SARI=1
#Male=0; Female=1
#No=0; Yes=1
#Alive=0; Deceased=1
#Beginning=0; Middle=1; End=2
#<15=0; 15-59=1; >=60=2
#Mild=0; Moderate=1; Severe=2

varlistReassort2 <- c("metadataReassort$Surveillance", "metadataReassort$Sex", "metadataRea
ssort$ca_vacci", "metadataReassort$ca_status", "metadataReassort$ca_antibio", "metadataReas
sort$asthma_respi", "metadataReassort$cardio", "metadataReassort$obesity", "metadataReassor
t$diabete", "metadataReassort$renal", "metadataReassort$immuno", "metadataReassort$neuro", "
metadataReassort$hepato", "metadataReassort$ca_icuhosp")

varlistReassort3 <- c("metadataReassort$Period", "metadataReassort$Age.cat", "metadataReass
ort$MildModerateSevere")

varlistReassort <- c("Surveillance", "Sex", "ca_vacci", "ca_status", "ca_antibio", "asthma_
respi", "cardio", "obesity", "diabete", "renal", "immuno", "neuro", "hepato", "ca_icuhosp",
"Period", "Age.cat", "MildModerateSevere")


Reassort.fun2 <- (paste("apply(Reassort,2,function(e) fisher.test(table(factor(e,levels=c(0
, 1)), factor(", varlistReassort2,", levels=c(0, 1))))$p.value)", sep=""))
Reassort.fun3 <- (paste("apply(Reassort,2,function(e) fisher.test(table(factor(e,levels=c(0
, 1)), factor(", varlistReassort3,", levels=c(0, 1, 2))))$p.value)", sep=""))
Reassort.fun <- c(Reassort.fun2,Reassort.fun3)
Reassort.fisher <- lapply(Reassort.fun, function(x) {eval(parse(text = x))})
names(Reassort.fisher) <- varlistReassort
#Reassort.fisher

Reassort.fun.cont2 <- (paste("apply(Reassort, 2, function(e)table(factor(e, levels=c(0,1)),
factor(", varlistReassort2,", levels=c(0, 1))))[,c(names(Reassort))]", sep=""))
Reassort.fun.cont3 <- (paste("apply(Reassort, 2, function(e)table(factor(e, levels=c(0,1)),
factor(", varlistReassort3,", levels=c(0, 1, 2))))[,c(names(Reassort))]", sep=""))
Reassort.fun.cont<-c(Reassort.fun.cont2,Reassort.fun.cont3)
Reassort.fisher.cont <- lapply(Reassort.fun.cont, function(x) {eval(parse(text = x))})
names(Reassort.fisher.cont) <- varlistReassort
Reassort.fisher.cont2 <- (melt(Reassort.fisher.cont))
Reassort.fisher.cont2$Name <- paste(Reassort.fisher.cont2$Var2,"_",Reassort.fisher.cont2$L1
,"_",Reassort.fisher.cont2$Var1, sep="")
rownames(Reassort.fisher.cont2)<-Reassort.fisher.cont2$Name
Reassort.fisher.cont2$Var1<-NULL
Reassort.fisher.cont2$Var2<-NULL
Reassort.fisher.cont2$L1<-NULL
Reassort.fisher.cont2$Name<-NULL
```

```
#Reassort.fisher.cont2


Reassort.fun.fdr2 <- noquote(paste("p.adjust(apply(Reassort,2,function(e) fisher.test(table
(factor(e,levels=c(0, 1)), factor(", varlistReassort2,", levels=c(0, 1))))$p.value),method=
\"fdr\")",                                                                          sep=""))
Reassort.fun.fdr3 <- noquote(paste("p.adjust(apply(Reassort,2,function(e) fisher.test(table
(factor(e,levels=c(0, 1)), factor(", varlistReassort3,", levels=c(0, 1, 2))))$p.value),meth
od=\"fdr\")",                                                                       sep=""))
Reassort.fun.fdr                 <-                c(Reassort.fun.fdr2,Reassort.fun.fdr3)
Reassort.fisher.fdr  <-  lapply(Reassort.fun.fdr,  function(x)  {eval(parse(text  =  x))}})
names(Reassort.fisher.fdr)                      <-                           varlistReassort
#Reassort.fisher.fdr

write.csv(Reassort.fisher,                        file.path("Results/Reassort.fisher.csv"))

write.csv(Reassort.fisher.cont2,             file.path("Results/Reassort.fisher.cont.csv"))

write.csv(Reassort.fisher.fdr, file.path("Results/Reassort.fisher.fdr.csv"))
```

Permutation tests

Reassortments

ILI vs SARI

Boxplot

```
ReassortCombined= read_excel("8. Supplementary File - Input files statistical analysis.xlsx
", sheet = "Reassortments - Combined", header = T, row.names = 1, sep = ",", quote = "/", s
tringsAsFactors=F)

metadataCombinedres <- metadataReassort[match(rownames(metadataReassort), as.character(meta
dataReassort$Reference)),]
ReassortCombined      <-      as.data.frame(cbind(ReassortCombined,      metadataReassort))

sumReassComboILIsubset <- (subset(ReassortCombined, Surveillance == "0", select = Reassortm
ent)                    %>%                            unlist)                          #93
sumReassComboSARIsubset <- (subset(ReassortCombined, Surveillance == "1", select = Reassort
ment)                   %>%                            unlist)                         #160
sumReassComboILI                          <-                       sum(sumReassComboILIsubset)

totalsumComboILI                          <-                       sum(sumReassComboILIsubset)


totalsumComboSARI                              <-                           replicate(10000,{
  SARIReass                   <-                      sample(sumReassComboSARIsubset,93)
  sum(SARIReass)
})

surveillanceReassarrayCombo               <-              data.frame(array("NA",dim=c(10000,2)))
colnames(surveillanceReassarrayCombo)                   <-                        c("ILI","SARI")
surveillanceReassarrayCombo$ILI                     <-                        (totalsumComboILI)
surveillanceReassarrayCombo$SARI                    <-                       (totalsumComboSARI)
surveillanceReassarrayCombo <- melt(surveillanceReassarrayCombo)

## No id variables; using all as measure variables

colnames(surveillanceReassarrayCombo)              <-               c("Surveillance","sum")
```

```
Reassort.fun2 <- (paste("apply(Reassort,2,function(e) fisher.test(table(factor(e,levels=c(0, 1)), factor(", varlistReassort2,", levels=c(0, 1))))$p.value)", sep=""))


#Boxplot
boxtotalReassComboSurveillance <- ggplot(surveillanceReassarrayCombo, aes(factor(Surveillance), sum), fill=as.factor(Surveillance)) + geom_boxplot(position = position_dodge(width=100))
boxtotalReassComboSurveillance <- boxtotalReassComboSurveillance + xlab("Surveillance system") + ylab("Sum of Reassortment (Combined method)") + guides(fill=guide_legend(title="Surveillance system"))
boxtotalReassComboSurveillance <- boxtotalReassComboSurveillance + geom_point(aes(y=sum, group=Surveillance), position = "jitter")
boxtotalReassComboSurveillance
```

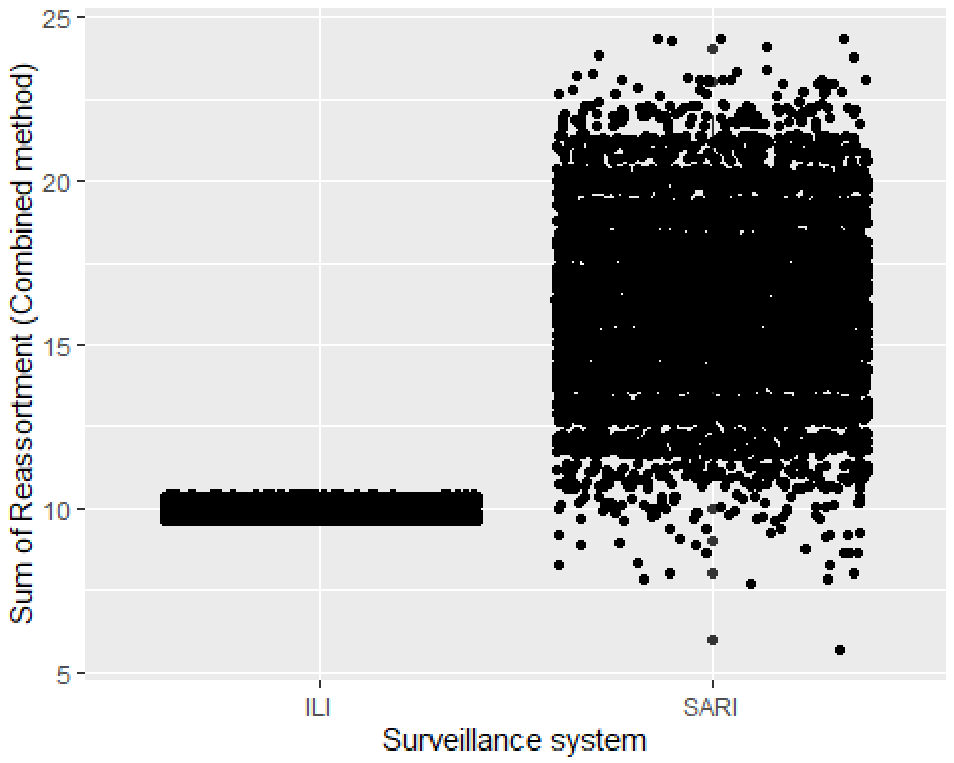


```
psurvCombo <- 0.975*(median(totalsumComboSARI) - median(sumReassComboILI))
psurvCombo1 <- median(totalsumComboSARI)+psurvCombo
psurvCombo2 <- median(totalsumComboSARI)-psurvCombo
spsurvCombo <- sum(length(which(totalsumComboSARI>psurvCombo1)))+sum(length(which(totalsumComboSARI<psurvCombo2)))
cat("P-value (Surveillance) =",spsurvCombo/10000,"\n")

## P-value (Surveillance) = 0.0208

ggsave("Boxplot_Reassortment_Combined_prob_0.95_Surveillence.png", plot = boxtotalReassComboSurveillance, device = NULL, path = “X”, width = 10, height =8, dpi = 150)
```

Permutation 1 -> max

```
sumasympComboILI <- list()
for(x in 1:length(sumReassComboILIsubset)) {
  sumasympComboILI[[x]] <- replicate(10000,{
  ILIasympReass <- sample(sumReassComboILIsubset,x)
  sum(ILIasympReass)
  })
```

```
}
sumasympComboILI2<-                                                    as.data.frame(sumasympComboILI)
colnames(sumasympComboILI2)             <-             c(1:length(sumReassComboILIsubset))
sumasympComboILI2 <- melt(sumasympComboILI2)

## No id variables; using all as measure variables

colnames(sumasympComboILI2)                     <-                          c("Number","sum")
sumasympComboILI2$Surveillance            <-             rep("ILI",nrow(sumasympComboILI2))

sumasympComboSARI                                <-                                    list()
for(x                in               1:length(sumReassComboILIsubset))                   {
  sumasympComboSARI[[x]]                        <-                        replicate(10000,{
  SARIasympReass                  <-                  sample(sumReassComboSARIsubset,x)
  sum(SARIasympReass)
  })
}
sumasympComboSARI2<-                                                  as.data.frame(sumasympComboSARI)
colnames(sumasympComboSARI2)             <-            c(1:length(sumReassComboILIsubset))
sumasympComboSARI2 <- melt(sumasympComboSARI2)

## No id variables; using all as measure variables

colnames(sumasympComboSARI2)                     <-                          c("Number","sum")
sumasympComboSARI2$Surveillance           <-            rep("SARI",nrow(sumasympComboSARI2))

surveillancesumasympComboarray          <-         rbind(sumasympComboILI2,sumasympComboSARI2)
surveillancesumasympComboarray$Number  <-  as.integer(surveillancesumasympComboarray$Number)

NumtotalReassSurveillance <- ggerrorplot(surveillancesumasympComboarray, x = "Number", y =
"sum", desc_stat = "mean", color = "Surveillance", size = 0.1,xlab = "Number of samples", y
lab  =  "Sum  of  Reassortments  (Combined  method)",  font.xtickslab  =  4,  x.text.angle  =  90)
NumtotalReassSurveillance
```

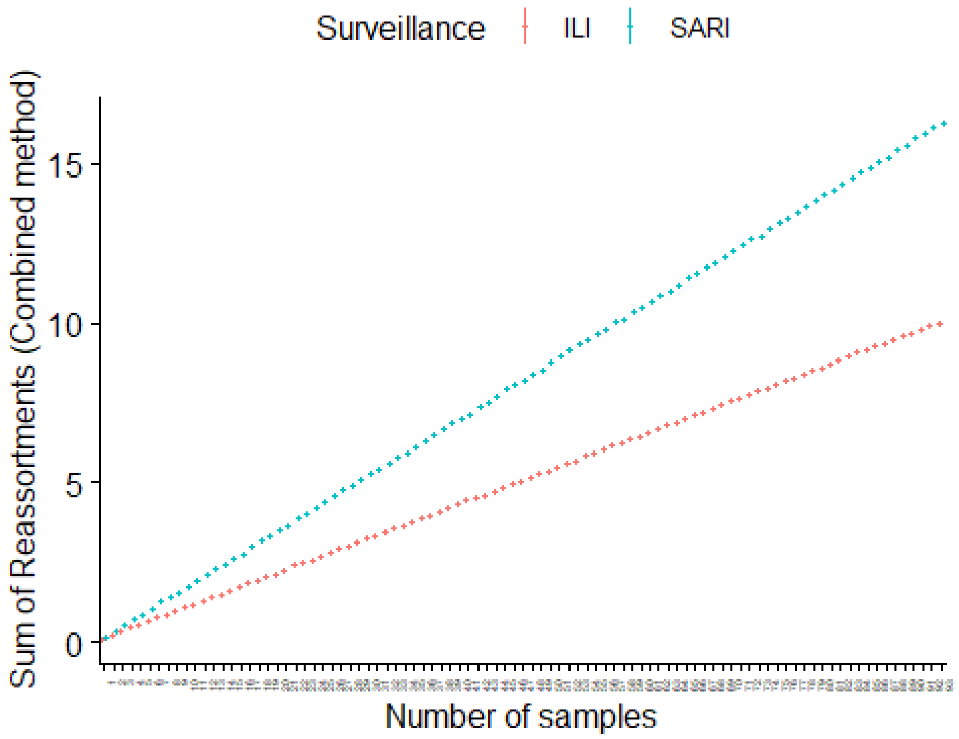


```
ggsave("NumReassortment_Combined_prob_0.95_Surveillence.png", plot = NumtotalReassSurveilla
nce, device = NULL, path = “X”, width = 10, height =8, dpi = 150)
```

```
```

Supplementary Tables and Figures

Table S1: Additional reference sequences used for phylogenomic analysis

HA Clade	ID	GISAID Number
**Group 3C 2a**	A/Hong Kong/4801/2014	EPI_ISL_198222
**Group 3C 2a**	A/Uganda/0215/2017	EPI_ISL_255341
**Group 3C 2a 1**	A/Singapore/Infimh-16-0019/2016	EPI_ISL_239803
**Group 3C 2a 1**	A/Tokyo/16492/2016	EPI_ISL_257919
**Group 3C 2a 1**	A/Michigan/22/2017	EPI_ISL_252973
**Group 3C 2a 1**	A/Hawaii/58/2016	EPI_ISL_232539
**Group 3C 2a 1**	A/Yokohama/120/2016	EPI_ISL_238612
**Group 3C 2a 1**	A/Saint-Etienne/845/2017	EPI_ISL_331771
**Group 3C 2a 1**	A/Fukushima/113/2016	EPI_ISL_224696
**Group 3C 2a 1a**	A/Greece/4/2017	EPI_ISL_257379
**Group 3C 2a 1a**	A/Norway/3806/2016	EPI_ISL_247959
**Group 3C 2a 1a**	A/Alaska/37/2016	EPI_ISL_244670
**Group 3C 2a 1a**	A/Puerto Rico/12/2017	EPI_ISL_274444
**Group 3C 2a 1b**	A/England/74560298/2017	EPI_ISL_286871
**Group 3C 2a 1b**	A/Alsace/1746/2018	EPI_ISL_310233
**Group 3C 2a 1b**	A/Belgium/S0281/2019	EPI_ISL_357163
**Group 3C 2a 1b**	A/Stockholm/44/2017	EPI_ISL_285973
**Group 3C 2a 1b**	A/Sweden/37/2018	EPI_ISL_310245
**Group 3C 2a 1b**	A/Hawaii/44/2018	EPI_ISL_316425
**Group 3C 2a 2**	A/Norway/4465/2016	EPI_ISL_239788
**Group 3C 2a 2**	A/Nantes/1441/2017	EPI_ISL_275863
**Group 3C 2a 3**	A/Norway/4849/2016@20161202	EPI_ISL_275859
**Group 3C 2a 3**	A/Toulon/2291/2016	EPI_ISL_331772
**Group 3C 2a 4**	A/Yokohama/138/2017	EPI_ISL_292541
**Group 3C 2a 4**	A/South Australia/1060/2017	EPI_ISL_289876

Table S2: Sequences extracted from GISAID to construct the custom-built Nextstrain instance

Isolate Name	Isolate ID	Isolate Name	Isolate ID	Isolate Name	Isolate ID
**A/Stockholm/40/2013**	EPI_ISL_155657	A/Pennsylvania/162/2017	EPI_ISL_289393	A/Guadeloupe/632/2019	EPI_ISL_368183
**A/Stockholm/4/2014**	EPI_ISL_156993	A/Pennsylvania/163/2017	EPI_ISL_289394	A/Guadeloupe/629/2019	EPI_ISL_368184
**A/Gavle/1/2014**	EPI_ISL_157000	A/Pennsylvania/172/2017	EPI_ISL_289396	A/Florida/78/2019	EPI_ISL_368197
**A/Sweden/7/2014**	EPI_ISL_159141	A/Pennsylvania/176/2017	EPI_ISL_289398	A/Wisconsin/555/2019	EPI_ISL_368201
**A/Rio Grande do Sul/23/2014**	EPI_ISL_166793	A/Pennsylvania/178/2017	EPI_ISL_289400	A/Cote D'Ivoire/643/2019	EPI_ISL_368204
**A/Stockholm/21/2014**	EPI_ISL_167059	A/Pennsylvania/190/2017	EPI_ISL_289401	A/Colorado/9466/2019	EPI_ISL_368889
**A/Mississippi/04/2014**	EPI_ISL_167406	A/Pennsylvania/198/2017	EPI_ISL_289403	A/Florida/9473/2019	EPI_ISL_368896
**A/Maine/04/2014**	EPI_ISL_167411	A/Pennsylvania/223/2017	EPI_ISL_289407	A/Georgia/9480/2019	EPI_ISL_368902
**A/Florida/38/2014**	EPI_ISL_167417	A/Pennsylvania/230/2017	EPI_ISL_289410	A/Germany/9482/2019	EPI_ISL_368904
**A/Stockholm/24/2014**	EPI_ISL_167961	A/Pennsylvania/250/2017	EPI_ISL_289412	A/Italy/9498/2019	EPI_ISL_368912
**A/Montana/11/2014**	EPI_ISL_168099	A/Pennsylvania/144/2016	EPI_ISL_289414	A/Italy/9499/2019	EPI_ISL_368913
**A/Colorado/19/2014**	EPI_ISL_168102	A/Pennsylvania/145/2016	EPI_ISL_289430	A/Japan/9505/2019	EPI_ISL_368916
**A/Connecticut/19/2014**	EPI_ISL_168702	A/Pennsylvania/31/2017	EPI_ISL_289435	A/Massachusetts/9508/2019	EPI_ISL_368919
**A/Connecticut/21/2014**	EPI_ISL_168703	A/Pennsylvania/40/2017	EPI_ISL_289438	A/New York/9512/2019	EPI_ISL_368923
**A/Florida/45/2014**	EPI_ISL_168705	A/Pennsylvania/73/2017	EPI_ISL_289441	A/New York/9517/2019	EPI_ISL_368928
**A/Louisiana/14/2014**	EPI_ISL_168712	A/Pennsylvania/121/2017	EPI_ISL_289448	A/New York/9518/2019	EPI_ISL_368929
**A/New Hampshire/21/2014**	EPI_ISL_168716	A/Pennsylvania/130/2017	EPI_ISL_289450	A/New York/9520/2019	EPI_ISL_368931
**A/Oregon/09/2014**	EPI_ISL_168721	A/Kentucky/28/2017	EPI_ISL_289530	A/New York/9537/2019	EPI_ISL_368947
**A/Montana/14/2014**	EPI_ISL_169097	A/Virginia/35/2017	EPI_ISL_289534	A/North Carolina/9546/2019	EPI_ISL_368956
**A/Iowa/16/2014**	EPI_ISL_169103	A/North Carolina/42/2017	EPI_ISL_289536	A/Ohio/9556/2019	EPI_ISL_368965
**A/Nevada/18/2014**	EPI_ISL_169123	A/Indonesia/Nihrd-Bjm576/2017	EPI_ISL_289548	A/Ohio/9560/2019	EPI_ISL_368967
**A/Stockholm/26/2014**	EPI_ISL_169134	A/Indonesia/Nihrd-Pal350/2017	EPI_ISL_289550	A/South Carolina/9576/2019	EPI_ISL_368981
**A/Gothenburg/4/2014**	EPI_ISL_169255	A/Indonesia/Nihrd-Mks410/2017	EPI_ISL_289551	A/Texas/9581/2019	EPI_ISL_368983
**A/Sweden/76/2014**	EPI_ISL_169256	A/Indonesia/Nihrd-Mlg455/2017	EPI_ISL_289552	A/Washington/9597/2019	EPI_ISL_368997
**A/Stockholm/28/2014**	EPI_ISL_169258	A/Indonesia/Nihrd-Mks408/2017	EPI_ISL_289553	A/England/9616/2019	EPI_ISL_369014
**A/Malmoe/5/2014**	EPI_ISL_169259	A/Tennessee/59/2017	EPI_ISL_289756	A/Florida/9621/2019	EPI_ISL_369018
**A/Louisiana/17/2014**	EPI_ISL_169292	A/La Serena/76599/2017	EPI_ISL_290657	A/Idaho/9626/2019	EPI_ISL_369022
**A/North Carolina/35/2014**	EPI_ISL_169304	A/Madagascar/3095/2017	EPI_ISL_290659	A/Missouri/9629/2019	EPI_ISL_369025
**A/New Jersey/27/2014**	EPI_ISL_169305	A/Madagascar/3319/2017	EPI_ISL_290661	A/New York/9632/2019	EPI_ISL_369026
**A/Nevada/20/2014**	EPI_ISL_169314	A/North Carolina/46/2017	EPI_ISL_290667	A/South Carolina/9641/2019	EPI_ISL_369033
**A/North Carolina/28/2014**	EPI_ISL_169317	A/Texas/313/2017	EPI_ISL_290672	A/South Carolina/9644/2019	EPI_ISL_369036
**A/Iowa/14/2014**	EPI_ISL_169318	A/Arkansas/44/2017	EPI_ISL_290689	A/Utah/9652/2019	EPI_ISL_369042
**A/Alaska/46/2014**	EPI_ISL_169326	A/Utah/44/2017	EPI_ISL_290719	A/England/9662/2019	EPI_ISL_369070
**A/Texas/52/2014**	EPI_ISL_169329	A/Montana/41/2017	EPI_ISL_290722	A/Germany/9669/2019	EPI_ISL_369077
**A/Massachusetts/14/2014**	EPI_ISL_169334	A/Florida/86/2017	EPI_ISL_290864	A/Germany/9677/2019	EPI_ISL_369084
**A/Illinois/05/2014**	EPI_ISL_169344	A/North Carolina/40/2017	EPI_ISL_290865	A/Italy/9688/2019	EPI_ISL_369093
**A/Kansas/10/2014**	EPI_ISL_169346	A/Florida/93/2017	EPI_ISL_290869	A/Italy/9690/2019	EPI_ISL_369095
**A/Hawaii/36/2014**	EPI_ISL_169509	A/Mexico/2496/2017	EPI_ISL_291626	A/Missouri/9695/2019	EPI_ISL_369100
**A/Louisiana/28/2014**	EPI_ISL_169907	A/Mexico/2514/2017	EPI_ISL_291627	A/New York/9698/2019	EPI_ISL_369103
**A/Sweden/77/2014**	EPI_ISL_170836	A/Mexico/2557/2017	EPI_ISL_291630	A/New York/9699/2019	EPI_ISL_369104
**A/Stockholm/34/2014**	EPI_ISL_170839	A/Mexico/2567/2017	EPI_ISL_291631	A/Washington/9721/2019	EPI_ISL_369124
**A/Stockholm/35/2014**	EPI_ISL_170840	A/Mexico/2570/2017	EPI_ISL_291632	A/Alaska/7957/2019	EPI_ISL_369733
**A/Skovde/1/2014**	EPI_ISL_170841	A/Mexico/2577/2017	EPI_ISL_291633	A/Colorado/7961/2019	EPI_ISL_369736
**A/Skovde/1/2015**	EPI_ISL_170842	A/Mexico/2610/2017	EPI_ISL_291634	A/England/7965/2019	EPI_ISL_369739
**A/Hawaii/63/2014**	EPI_ISL_172518	A/Mexico/2611/2017	EPI_ISL_291635	A/England/7968/2019	EPI_ISL_369742
**A/Sweden/4/2015**	EPI_ISL_172914	A/Mexico/2638/2017	EPI_ISL_291639	A/Florida/7972/2019	EPI_ISL_369745
**A/Lund/1/2015**	EPI_ISL_172918	A/Parana/929/2017	EPI_ISL_291642	A/Japan/8000/2019	EPI_ISL_369762
**A/Stockholm/8/2015**	EPI_ISL_172930	A/Rio Grande Do Sul/663/2017	EPI_ISL_291644	A/Japan/8001/2019	EPI_ISL_369763
**A/Stockholm/9/2015**	EPI_ISL_172931	A/Connecticut/38/2017	EPI_ISL_291676	A/Japan/8002/2019	EPI_ISL_369764
**A/Sweden/8/2015**	EPI_ISL_174008	A/California/154/2017	EPI_ISL_291686	A/Nebraska/8010/2019	EPI_ISL_369769
**A/Stockholm/12/2015**	EPI_ISL_177058	A/Pakistan/199/2017	EPI_ISL_291706	A/New Jersey/8025/2019	EPI_ISL_369778
**A/Stockholm/13/2015**	EPI_ISL_177059	A/Pakistan/225/2017	EPI_ISL_291710	A/New York/8043/2019	EPI_ISL_369792
**A/Stockholm/14/2015**	EPI_ISL_177060	A/Pakistan/232/2017	EPI_ISL_291713	A/Oklahoma/8059/2019	EPI_ISL_369803
**A/Stockholm/16/2015**	EPI_ISL_177062	A/Pakistan/198/2017	EPI_ISL_291714	A/South Carolina/8063/2019	EPI_ISL_369806
**A/Stockholm/18/2015**	EPI_ISL_177064	A/Montana/46/2017	EPI_ISL_291719	A/Texas/8070/2019	EPI_ISL_369813
**A/Stockholm/20/2015**	EPI_ISL_177071	A/Philippines/0782/2017	EPI_ISL_291736	A/Texas/8072/2019	EPI_ISL_369814
**A/Sweden/17/2015**	EPI_ISL_177078	A/Philippines/0797/2017	EPI_ISL_291737	A/Texas/8078/2019	EPI_ISL_369816
**A/Malmoe/2/2015**	EPI_ISL_177093	A/Philippines/0944/2017	EPI_ISL_291739	A/Virginia/8086/2019	EPI_ISL_369822
**A/Karlstad/3/2015**	EPI_ISL_177688	A/Galicia/2467/2017	EPI_ISL_291781	A/Colorado/8111/2019	EPI_ISL_369981
**A/Colombia/4468/2015**	EPI_ISL_179011	A/Sweden/81/2017	EPI_ISL_292040	A/England/8122/2019	EPI_ISL_369984
**A/Colombia/2473/2015**	EPI_ISL_179012	A/Sweden/85/2017	EPI_ISL_292057	A/Montana/8150/2019	EPI_ISL_369999
**A/Colombia/4352/2015**	EPI_ISL_179013	A/Singapore/GP2271/2017	EPI_ISL_292445	A/Montana/8151/2019	EPI_ISL_370000
**A/NAGANO/2202/2015**	EPI_ISL_179016	A/Singapore/GP2439/2017	EPI_ISL_292448	A/Nevada/8155/2019	EPI_ISL_370002
**A/North Carolina/25/2015**	EPI_ISL_181075	A/Singapore/KK1381/2017	EPI_ISL_292456	A/New York/8177/2019	EPI_ISL_370008
**A/Washington/20/2014**	EPI_ISL_191431	A/Santa Catarina/737/2017	EPI_ISL_292523	A/South Korea/8206/2019	EPI_ISL_370021
**A/Ecuador/2384/2015**	EPI_ISL_194960	A/Kentucky/34/2017	EPI_ISL_292585	A/South Korea/8208/2019	EPI_ISL_370023
**A/Hawaii/47/2015**	EPI_ISL_194966	A/Nebraska/20/2017	EPI_ISL_292590	A/Texas/8218/2019	EPI_ISL_370029
**A/Florida/57/2015**	EPI_ISL_194970	A/Texas/321/2017	EPI_ISL_292615	A/Texas/8220/2019	EPI_ISL_370030
**A/Florida/58/2015**	EPI_ISL_194976	A/Netherlands/10029/2018	EPI_ISL_293132	A/Japan/8266/2019	EPI_ISL_370113
**A/Alaska/150/2015**	EPI_ISL_195832	A/Colombia/6646/2017	EPI_ISL_295849	A/Mississippi/8288/2019	EPI_ISL_370128
**A/Texas/65/2015**	EPI_ISL_195843	A/Kentucky/01/2018	EPI_ISL_295859	A/New York/8306/2019	EPI_ISL_370140
**A/Ecuador/2369/2015**	EPI_ISL_195876	A/Colombia/6062/2017	EPI_ISL_296077	A/North Dakota/8312/2019	EPI_ISL_370145
**A/Colombia/5614/2015**	EPI_ISL_195896	A/Colombia/6187/2017	EPI_ISL_296080	A/Ohio/8331/2019	EPI_ISL_370159
**A/Guatemala/27/2015**	EPI_ISL_195897	A/Colombia/6380/2017	EPI_ISL_296081	A/Oklahoma/8335/2019	EPI_ISL_370161
**A/OKAYAMA/2/2015**	EPI_ISL_196619	A/Colombia/6434/2017	EPI_ISL_296082	A/Oklahoma/8336/2019	EPI_ISL_370162
**A/Hawaii/35/2015**	EPI_ISL_197251	A/Colombia/6446/2017	EPI_ISL_296083	A/South Korea/8352/2019	EPI_ISL_370172
**A/Hawaii/36/2015**	EPI_ISL_197252	A/Colombia/6525/2017	EPI_ISL_296084	A/Texas/8368/2019	EPI_ISL_370182
**A/Oregon/16/2015**	EPI_ISL_197271	A/Costa Rica/7285/2017	EPI_ISL_296091	A/England/7821/2019	EPI_ISL_370581
**A/Paraguay/6991/2015**	EPI_ISL_197276	A/California/02/2018	EPI_ISL_296116	A/Germany/7830/2018	EPI_ISL_370583
**A/Guatemala/19/2015**	EPI_ISL_197488	A/California/07/2018	EPI_ISL_296121	A/Guam/7834/2019	EPI_ISL_370586
**A/Guatemala/5915/2015**	EPI_ISL_197494	A/Missouri/02/2018	EPI_ISL_296155	A/Guam/7835/2019	EPI_ISL_370587
**A/Massachusetts/23/2015**	EPI_ISL_197495	A/Laos/3783/2017	EPI_ISL_296450	A/Italy/7837/2018	EPI_ISL_370588
**A/Guatemala/13/2015**	EPI_ISL_197560	A/Costa Rica/7181/2017	EPI_ISL_296472	A/Nebraska/7853/2019	EPI_ISL_370596
**A/Connecticut/01/2015**	EPI_ISL_197762	A/Costa Rica/6973/2017	EPI_ISL_296525	A/North Carolina/7864/2019	EPI_ISL_370604
**A/Alaska/78/2015**	EPI_ISL_197778	A/Maryland/99/2017	EPI_ISL_296562	A/Texas/7915/2019	EPI_ISL_370622
**A/Connecticut/02/2015**	EPI_ISL_197792	A/Georgia/34/2017	EPI_ISL_296568	A/Texas/7919/2018	EPI_ISL_370626

**A/Colombia/5402/2015**	EPI_ISL_199508	A/West Virginia/02/2018	EPI_ISL_296601	A/Texas/7927/2019	EPI_ISL_370630
**A/Colombia/235/2015**	EPI_ISL_199509	A/Saint-Petersburg/RII-144S/2017	EPI_ISL_297723	A/Texas/7942/2019	EPI_ISL_370637
**A/Colombia/5364/2015**	EPI_ISL_199511	A/Choibalsan/2919/2017	EPI_ISL_298313	A/Texas/7945/2019	EPI_ISL_370638
**A/Colombia/241/2015**	EPI_ISL_199513	A/Puerto Rico/01/2018	EPI_ISL_298356	A/Texas/7946/2019	EPI_ISL_370639
**A/Colombia/5215/2015**	EPI_ISL_199516	A/Peru/5217/2017	EPI_ISL_298367	A/Colorado/7683/2018	EPI_ISL_372018
**A/Uruguay/122/2015**	EPI_ISL_199517	A/Peru/806817/2017	EPI_ISL_298375	A/Florida/7695/2018	EPI_ISL_372023
**A/Uruguay/55/2015**	EPI_ISL_199522	A/Peru/751017/2017	EPI_ISL_298376	A/Florida/7696/2018	EPI_ISL_372024
**A/Brazil/8925/2015**	EPI_ISL_200676	A/Peru/7717/2017	EPI_ISL_298377	A/Georgia/7698/2018	EPI_ISL_372025
**A/Brazil/4996/2015**	EPI_ISL_200677	A/Maldives/1455/2017	EPI_ISL_298384	A/Guam/7699/2018	EPI_ISL_372026
**A/Brazil/6261/2015**	EPI_ISL_200678	A/Maldives/1438/2017	EPI_ISL_298385	A/New Jersey/7726/2018	EPI_ISL_372044
**A/Ecuador/572/2015**	EPI_ISL_200681	A/Alaska/99/2017	EPI_ISL_298573	A/New Jersey/7728/2019	EPI_ISL_372046
**A/Ecuador/1695/2015**	EPI_ISL_200682	A/Illinois/61/2017	EPI_ISL_298584	A/New York/7737/2018	EPI_ISL_372055
**A/Ohio/18/2015**	EPI_ISL_200687	A/Illinois/55/2017	EPI_ISL_298598	A/Ohio/7755/2018	EPI_ISL_372065
**A/Acre/133946-IEC/2015**	EPI_ISL_200698	A/Abu Dhabi/076/2017	EPI_ISL_299805	A/Ohio/7759/2018	EPI_ISL_372069
**A/Para/134244-IEC/2015**	EPI_ISL_200700	A/Abu Dhabi/078/2017	EPI_ISL_299806	A/Ohio/7760/2018	EPI_ISL_372070
**A/Para/134324-IEC/2015**	EPI_ISL_200701	A/Abu Dhabi/097/2017	EPI_ISL_299810	A/Utah/7791/2018	EPI_ISL_372085
**A/Puerto Rico/04/2015**	EPI_ISL_200708	A/Abu Dhabi/148/2017	EPI_ISL_299814	A/Arizona/9736/2019	EPI_ISL_372529
**A/Puerto Rico/05/2015**	EPI_ISL_200709	A/Niger/5254/2017	EPI_ISL_299832	A/England/9738/2019	EPI_ISL_372530
**A/Puerto Rico/06/2015**	EPI_ISL_200710	A/Niger/5267/2017	EPI_ISL_299834	A/Idaho/9741/2019	EPI_ISL_372531
**A/St. Vincent And Grenadines/422/2015**	EPI_ISL_200715	A/Ecuador/3868/2017	EPI_ISL_299890	A/Idaho/9742/2019	EPI_ISL_372532
**A/St. Vincent And Grenadines/424/2015**	EPI_ISL_200716	A/Brazil/6531/2017	EPI_ISL_299903	A/New York/9745/2019	EPI_ISL_372533
**A/Wisconsin/83/2015**	EPI_ISL_200759	A/Wyoming/34/2017	EPI_ISL_299905	A/Arizona/9016/2019	EPI_ISL_372600
**A/Malmoe/5/2015**	EPI_ISL_201102	A/Hong Kong/4991/2017	EPI_ISL_299913	A/California/9023/2019	EPI_ISL_372604
**A/Rio Grande Do Norte/135497-IEC/2015**	EPI_ISL_201226	A/Hong Kong/4992/2017	EPI_ISL_299914	A/Colorado/9026/2019	EPI_ISL_372607
**A/French Guiana/6101/2015**	EPI_ISL_201229	A/Hong Kong/4993/2017	EPI_ISL_299915	A/Florida/9037/2019	EPI_ISL_372621
**A/French Guiana/8217/2015**	EPI_ISL_201235	A/Delaware/03/2018	EPI_ISL_299918	A/Germany/9055/2019	EPI_ISL_372661
**A/Guatemala/6415/2015**	EPI_ISL_201239	A/Hong Kong/4976/2017	EPI_ISL_299941	A/Germany/9056/2019	EPI_ISL_372664
**A/Guatemala/5848/2015**	EPI_ISL_201241	A/Hong Kong/4983/2017	EPI_ISL_299942	A/Italy/9061/2019	EPI_ISL_372671
**A/Guatemala/8802/2015**	EPI_ISL_201242	A/Phetchaburi/2607/2017	EPI_ISL_299953	A/Italy/9062/2019	EPI_ISL_372674
**A/Arizona/22/2015**	EPI_ISL_201286	A/New Hampshire/06/2018	EPI_ISL_299967	A/Italy/9065/2019	EPI_ISL_372683
**A/Alaska/256/2015**	EPI_ISL_201296	A/Kansas/04/2018	EPI_ISL_300196	A/Nevada/9084/2019	EPI_ISL_372720
**A/Washington/44/2015**	EPI_ISL_201301	A/Texas/66/2018	EPI_ISL_300199	A/South Dakota/9114/2019	EPI_ISL_372742
**A/Vermont/08/2015**	EPI_ISL_201314	A/Texas/71/2018	EPI_ISL_300200	A/Texas/9131/2019	EPI_ISL_372757
**A/KOBE/262/2015**	EPI_ISL_201926	A/Lopburi/2461/2017	EPI_ISL_300214	A/Texas/9133/2019	EPI_ISL_372759
**A/California/91/2015**	EPI_ISL_202077	A/Massachusetts/09/2018	EPI_ISL_300216	A/Virginia/9140/2019	EPI_ISL_372766
**A/California/86/2015**	EPI_ISL_202082	A/Minas Gerais/594/2017	EPI_ISL_300387	A/Arizona/7327/2018	EPI_ISL_373406
**A/Guatemala/363/2015**	EPI_ISL_202086	A/Brazil/4558/2017	EPI_ISL_300755	A/California/7330/2018	EPI_ISL_373408
**A/Maine/43/2015**	EPI_ISL_202095	A/Brazil/5769/2017	EPI_ISL_300758	A/England/7334/2018	EPI_ISL_373410
**A/Maine/42/2015**	EPI_ISL_202096	A/Arkansas/04/2018	EPI_ISL_300771	A/England/7335/2018	EPI_ISL_373411
**A/Massachusetts/28/2015**	EPI_ISL_202097	A/Cote D'Ivoire/1580/2017	EPI_ISL_300773	A/England/7399/2018	EPI_ISL_373414
**A/West Virginia/23/2015**	EPI_ISL_202104	A/Iowa/04/2018	EPI_ISL_300780	A/Georgia/7340/2018	EPI_ISL_373418
**A/Guatemala/9877/2015**	EPI_ISL_202109	A/Cambodia/894/2017	EPI_ISL_300791	A/Germany/7345/2018	EPI_ISL_373422
**A/Peru/6915/2015**	EPI_ISL_202391	A/Cambodia/914/2017	EPI_ISL_300801	A/Germany/7352/2018	EPI_ISL_373429
**A/Wisconsin/86/2015**	EPI_ISL_202431	A/Cambodia/949/2017	EPI_ISL_300807	A/Germany/7357/2018	EPI_ISL_373432
**A/Puerto Rico/02/2015**	EPI_ISL_202828	A/Abu Dhabi/182/2017	EPI_ISL_300810	A/Guam/7362/2018	EPI_ISL_373444
**A/Washington/45/2015**	EPI_ISL_202841	A/Abu Dhabi/181/2017	EPI_ISL_300811	A/Hawaii/7411/2018	EPI_ISL_373445
**A/Florida/77/2015**	EPI_ISL_202857	A/Abu Dhabi/125/2017	EPI_ISL_300813	A/Italy/7366/2018	EPI_ISL_373447
**A/Colorado/15/2015**	EPI_ISL_202909	A/Cambodia/908/2017	EPI_ISL_300819	A/Italy/7368/2018	EPI_ISL_373449
**A/Hawaii/74/2015**	EPI_ISL_202921	A/Cote D'Ivoire/1549/2017	EPI_ISL_300840	A/Japan/7415/2018	EPI_ISL_373452
**A/Louisiana/06/2015**	EPI_ISL_202926	A/Netherlands/10214/2018	EPI_ISL_301300	A/Kentucky/7369/2018	EPI_ISL_373453
**A/Wyoming/19/2015**	EPI_ISL_202934	A/Linkoping/6/2017	EPI_ISL_302171	A/Maryland/7419/2018	EPI_ISL_373457
**A/New York/69/2015**	EPI_ISL_203422	A/Stockholm/55/2017	EPI_ISL_302172	A/Mississippi/7373/2018	EPI_ISL_373460
**A/Pernambuco/135596-IEC/2015**	EPI_ISL_203424	A/Stockholm/57/2017	EPI_ISL_302175	A/Mississippi/7420/2018	EPI_ISL_373462
**A/Puerto Rico/16/2015**	EPI_ISL_203441	A/Cardiff/7156/2017	EPI_ISL_302187	A/New Mexico/7375/2018	EPI_ISL_373463
**A/Arizona/23/2015**	EPI_ISL_203556	A/Sweden/4/2018	EPI_ISL_302267	A/North Dakota/7377/2018	EPI_ISL_373467
**A/Hawaii/77/2015**	EPI_ISL_203565	A/Stockholm/5/2018	EPI_ISL_302286	A/Ohio/7379/2018	EPI_ISL_373469
**A/Alaska/193/2015**	EPI_ISL_203578	A/Cardiff/7495/2017	EPI_ISL_302484	A/Oklahoma/7383/2018	EPI_ISL_373474
**A/Alaska/154/2015**	EPI_ISL_205168	A/Afghanistan/0838/2017	EPI_ISL_303061	A/Oklahoma/7384/2018	EPI_ISL_373475
**A/Alaska/158/2015**	EPI_ISL_205184	A/Missouri/11/2018	EPI_ISL_303065	A/Oklahoma/7385/2018	EPI_ISL_373476
**A/Argentina/11659/2015**	EPI_ISL_205218	A/South Dakota/09/2018	EPI_ISL_303069	A/South Dakota/7387/2018	EPI_ISL_373477
**A/New Hampshire/40/2015**	EPI_ISL_205239	A/Afghanistan/0689/2017	EPI_ISL_303077	A/South Korea/7428/2018	EPI_ISL_373480
**A/Iowa/40/2015**	EPI_ISL_205267	A/Brazil/4729/2017	EPI_ISL_303087	A/Texas/7431/2018	EPI_ISL_373485
**A/Bolivia/426/2015**	EPI_ISL_205276	A/New Mexico/12/2018	EPI_ISL_303105	A/Texas/7434/2017	EPI_ISL_373488
**A/Bolivia/502/2015**	EPI_ISL_205278	A/New Jersey/09/2018	EPI_ISL_303117	A/Virginia/7393/2018	EPI_ISL_373496
**A/Alaska/183/2015**	EPI_ISL_205635	A/New York/07/2018	EPI_ISL_303118	A/Virginia/7441/2018	EPI_ISL_373497
**A/Puerto Rico/17/2015**	EPI_ISL_205643	A/Peru/3517/2017	EPI_ISL_303134	A/Washington/7395/2018	EPI_ISL_373500
**A/California/112/2015**	EPI_ISL_205672	A/Massachusetts/12/2018	EPI_ISL_303136	A/Wyoming/7396/2018	EPI_ISL_373501
**A/California/113/2015**	EPI_ISL_205673	A/Brazil/3870/2017	EPI_ISL_303214	A/Alaska/7516/2018	EPI_ISL_376399
**A/Gothenburg/1/2015**	EPI_ISL_205781	A/Pakistan/775/2018	EPI_ISL_303220	A/New Jersey/7529/2018	EPI_ISL_376412
**A/Uppsala/7/2015**	EPI_ISL_205790	A/Wyoming/09/2018	EPI_ISL_303232	A/Florida/7550/2018	EPI_ISL_376492
**A/Florida/94/2015**	EPI_ISL_206112	A/Pakistan/08/2017	EPI_ISL_303234	A/Guam/7556/2018	EPI_ISL_376495
**A/Temuco/75349/2015**	EPI_ISL_206115	A/Pakistan/718/2018	EPI_ISL_303236	A/South Korea/7646/2018	EPI_ISL_376540
**A/Concepcion/75466/2015**	EPI_ISL_206116	A/Cambodia/852/2017	EPI_ISL_303237	A/South Korea/7650/2018	EPI_ISL_376542
**A/Brazil/0523/2015**	EPI_ISL_206123	A/Honduras/494/2017	EPI_ISL_303241	A/Alaska/6939/2018	EPI_ISL_376553
**A/Brazil/0515/2015**	EPI_ISL_206125	A/Honduras/540/2017	EPI_ISL_303242	A/Germany/6943/2018	EPI_ISL_376557
**A/Brazil/0636/2015**	EPI_ISL_206126	A/Honduras/563/2017	EPI_ISL_303243	A/Italy/6950/2018	EPI_ISL_376564
**A/Kazakhstan/50/2015**	EPI_ISL_206131	A/Honduras/560/2017	EPI_ISL_303246	A/Japan/6954/2018	EPI_ISL_376568
**A/Alaska/127/2015**	EPI_ISL_206139	A/Honduras/538/2017	EPI_ISL_303257	A/Maryland/6961/2018	EPI_ISL_376575
**A/Alaska/194/2015**	EPI_ISL_206153	A/Brazil/8285/2017	EPI_ISL_303258	A/Maryland/6962/2018	EPI_ISL_376576
**A/Mali/56 CS/2015**	EPI_ISL_206171	A/Afghanistan/0837/2017	EPI_ISL_303260	A/Maryland/6963/2018	EPI_ISL_376577
**A/Mali/107 CS/2015**	EPI_ISL_206191	A/New York/13/2018	EPI_ISL_304307	A/Ohio/6965/2018	EPI_ISL_376579
**A/Mali/198 CH/2015**	EPI_ISL_206197	A/Maine/12/2018	EPI_ISL_304315	A/South Korea/6968/2018	EPI_ISL_376582
**A/Mali/13 HOP/2015**	EPI_ISL_206198	A/North Carolina/16/2018	EPI_ISL_304336	A/Texas/6973/2018	EPI_ISL_376587
**A/Puerto Rico/29/2015**	EPI_ISL_206217	A/Afghanistan/0691/2017	EPI_ISL_304979	A/Texas/6975/2018	EPI_ISL_376589
**A/Puerto Rico/36/2015**	EPI_ISL_206223	A/Congo/450/2017	EPI_ISL_304993	A/Texas/6977/2018	EPI_ISL_376591
**A/Puerto Rico/39/2015**	EPI_ISL_206226	A/Congo/510/2017	EPI_ISL_304994	A/Texas/6987/2018	EPI_ISL_376601
**A/Puerto Rico/40/2015**	EPI_ISL_206227	A/Congo/428/2017	EPI_ISL_304995	A/Texas/6997/2017	EPI_ISL_376611
**A/Puerto Rico/47/2015**	EPI_ISL_206230	A/Congo/467/2017	EPI_ISL_304996	A/Texas/6998/2017	EPI_ISL_376612

**A/Puerto Rico/50/2015**	EPI_ISL_206231	A/St. Petersburg/RII-308S/2017	EPI_ISL_305010	A/Texas/6999/2017	EPI_ISL_376613
**A/Antsirabe/3857/2015**	EPI_ISL_206244	A/Hawaii/16/2018	EPI_ISL_305074	A/Texas/7001/2017	EPI_ISL_376615
**A/Michigan/78/2015**	EPI_ISL_207010	A/Delaware/16/2018	EPI_ISL_305091	A/Virginia/7005/2018	EPI_ISL_376619
**A/California/118/2015**	EPI_ISL_207016	A/Utah/15/2018	EPI_ISL_305102	A/Colorado/7010/2018	EPI_ISL_376624
**A/Nigeria/235/2015**	EPI_ISL_207025	A/Iowa/12/2018	EPI_ISL_305281	A/Florida/7018/2017	EPI_ISL_376632
**A/Pennsylvania/35/2015**	EPI_ISL_207415	A/Kansas/11/2018	EPI_ISL_305306	A/Florida/7019/2018	EPI_ISL_376633
**A/Bangladesh/908005/2015**	EPI_ISL_208575	A/Colorado/17/2018	EPI_ISL_305803	A/Florida/7020/2018	EPI_ISL_376634
**A/California/126/2015**	EPI_ISL_208591	A/Cambodia/1054/2017	EPI_ISL_305838	A/Georgia/7021/2018	EPI_ISL_376635
**A/California/02/2016**	EPI_ISL_208597	A/Cambodia/1004/2017	EPI_ISL_305840	A/Georgia/7022/2018	EPI_ISL_376636
**A/Florida/01/2016**	EPI_ISL_208609	A/New Hampshire/18/2018	EPI_ISL_306207	A/New York/7035/2018	EPI_ISL_376649
**A/Bangladesh/13004/2015**	EPI_ISL_208611	A/Kentucky/18/2018	EPI_ISL_306210	A/North Carolina/7037/2018	EPI_ISL_376651
**A/New York/908/2004**	EPI_ISL_21006	A/Kentucky/17/2018	EPI_ISL_306211	A/South Carolina/7043/2018	EPI_ISL_376657
**A/Trinidad/2603/2015**	EPI_ISL_211680	A/Connecticut/19/2018	EPI_ISL_306212	A/Utah/7052/2018	EPI_ISL_376666
**A/South Africa/3915/2015**	EPI_ISL_211682	A/Michigan/52/2018	EPI_ISL_306223	A/England/7126/2018	EPI_ISL_376708
**A/South Africa/4033/2015**	EPI_ISL_211683	A/Hawaii/27/2018	EPI_ISL_306226	A/England/7127/2018	EPI_ISL_376709
**A/Mali/141 CI/2015**	EPI_ISL_211684	A/Virginia/19/2018	EPI_ISL_306233	A/Germany/7131/2018	EPI_ISL_376713
**A/Mali/110 CH/2015**	EPI_ISL_211685	A/Washington/68/2018	EPI_ISL_306239	A/Germany/7132/2018	EPI_ISL_376714
**A/Mali/136 CI/2015**	EPI_ISL_211694	A/Kazakhstan/049/2018	EPI_ISL_306256	A/Germany/7134/2018	EPI_ISL_376716
**A/South Africa/4438/2015**	EPI_ISL_211705	A/New Mexico/17/2018	EPI_ISL_306858	A/Germany/7135/2018	EPI_ISL_376717
**A/Alaska/269/2015**	EPI_ISL_211896	A/Louisiana/16/2018	EPI_ISL_306868	A/Germany/7137/2018	EPI_ISL_376719
**A/Florida/89/2015**	EPI_ISL_211903	A/Louisiana/14/2018	EPI_ISL_306870	A/Germany/7141/2018	EPI_ISL_376723
**A/New York/07/2016**	EPI_ISL_211917	A/Arizona/23/2018	EPI_ISL_306873	A/Guam/7145/2018	EPI_ISL_376727
**A/Tanzania/670/2015**	EPI_ISL_211931	A/Arizona/20/2018	EPI_ISL_306876	A/Idaho/7148/2018	EPI_ISL_376730
**A/Washington/76/2015**	EPI_ISL_211936	A/Washington/74/2018	EPI_ISL_306879	A/Italy/7152/2018	EPI_ISL_376734
**A/Taiwan/1090/2015**	EPI_ISL_211940	A/Minnesota/24/2018	EPI_ISL_306880	A/Italy/7156/2018	EPI_ISL_376738
**A/Cambodia/1137/2015**	EPI_ISL_212050	A/Montana/23/2018	EPI_ISL_306884	A/Japan/7159/2018	EPI_ISL_376741
**A/Colombia/6276/2015**	EPI_ISL_212051	A/Netherlands/10523/2018	EPI_ISL_306979	A/Japan/7160/2018	EPI_ISL_376742
**A/Colombia/6308/2015**	EPI_ISL_212052	A/Sweden/26/2018	EPI_ISL_307080	A/Japan/7163/2018	EPI_ISL_376745
**A/Colombia/7207/2015**	EPI_ISL_212053	A/Sweden/27/2018	EPI_ISL_307083	A/Kentucky/7164/2018	EPI_ISL_376746
**A/Colorado/28/2015**	EPI_ISL_212055	A/Dominican Republic/655/2017	EPI_ISL_307860	A/Mississippi/7166/2018	EPI_ISL_376748
**A/Pennsylvania/33/2015**	EPI_ISL_212059	A/Dominican Republic/693/2017	EPI_ISL_307863	A/Mississippi/7167/2017	EPI_ISL_376749
**A/Pennsylvania/52/2015**	EPI_ISL_212060	A/Abu Dhabi/221/2017	EPI_ISL_307867	A/South Dakota/7178/2018	EPI_ISL_376760
**A/Brazil/23262/2015**	EPI_ISL_212137	A/Abu Dhabi/243/2018	EPI_ISL_307870	A/South Korea/7182/2018	EPI_ISL_376764
**A/California/05/2016**	EPI_ISL_212138	A/Abu Dhabi/241/2018	EPI_ISL_307871	A/South Korea/7184/2018	EPI_ISL_376766
**A/Cambodia/0895/2015**	EPI_ISL_212144	A/Abu Dhabi/259/2018	EPI_ISL_307874	A/South Korea/7186/2018	EPI_ISL_376768
**A/Cambodia/0917/2015**	EPI_ISL_212145	A/Kuwait/1072/2018	EPI_ISL_307878	A/South Korea/7187/2018	EPI_ISL_376769
**A/Colorado/03/2016**	EPI_ISL_212148	A/Kuwait/1235/2018	EPI_ISL_307879	A/South Korea/7188/2018	EPI_ISL_376770
**A/Costa Rica/3677/2015**	EPI_ISL_212155	A/Connecticut/22/2018	EPI_ISL_307883	A/South Korea/7189/2018	EPI_ISL_376771
**A/Costa Rica/4817/2015**	EPI_ISL_212157	A/Georgia/22/2018	EPI_ISL_307884	A/Spain/7190/2018	EPI_ISL_376772
**A/Hawaii/03/2016**	EPI_ISL_212160	A/Ohio/16/2018	EPI_ISL_308940	A/Spain/7191/2018	EPI_ISL_376773
**A/Nevada/03/2016**	EPI_ISL_212368	A/New Jersey/17/2018	EPI_ISL_308942	A/Washington/7195/2018	EPI_ISL_376777
**A/Bangladesh/2743/2015**	EPI_ISL_212961	A/Massachusetts/19/2018	EPI_ISL_308956	A/Washington/7196/2018	EPI_ISL_376778
**A/California/11/2016**	EPI_ISL_212964	A/Virginia/23/2018	EPI_ISL_308959	A/Wyoming/7198/2018	EPI_ISL_376780
**A/Colombia/6438/2015**	EPI_ISL_212974	A/Kentucky/20/2018	EPI_ISL_308981	A/Arizona/7201/2018	EPI_ISL_376783
**A/Colombia/7113/2015**	EPI_ISL_212978	A/New Hampshire/22/2018	EPI_ISL_308985	A/Arkansas/7202/2018	EPI_ISL_376784
**A/Colorado/07/2016**	EPI_ISL_212980	A/Virginia/25/2018	EPI_ISL_308994	A/Colorado/7203/2018	EPI_ISL_376785
**A/Guatemala/4340/2015**	EPI_ISL_212986	A/Maryland/34/2018	EPI_ISL_308998	A/Colorado/7204/2018	EPI_ISL_376786
**A/Guatemala/5039/2015**	EPI_ISL_212987	A/New York/31/2018	EPI_ISL_309009	A/Colorado/7205/2018	EPI_ISL_376787
**A/Michigan/77/2015**	EPI_ISL_213007	A/Oregon/22/2018	EPI_ISL_309011	A/Delaware/7206/2018	EPI_ISL_376788
**A/Minnesota/61/2015**	EPI_ISL_213008	A/Washington/83/2018	EPI_ISL_309015	A/England/7208/2018	EPI_ISL_376790
**A/New York/13/2016**	EPI_ISL_213010	A/Utah/27/2018	EPI_ISL_309016	A/England/7210/2018	EPI_ISL_376792
**A/Nonthaburi/588/2015**	EPI_ISL_213011	A/Abu Dhabi/225/2017	EPI_ISL_309021	A/England/7211/2018	EPI_ISL_376793
**A/Vietnam/GS150287/2015**	EPI_ISL_213026	A/Texas/90/2018	EPI_ISL_309025	A/Florida/7214/2018	EPI_ISL_376796
**A/Vietnam/GS150974/2015**	EPI_ISL_213027	A/Connecticut/15/2018	EPI_ISL_309030	A/Germany/7218/2018	EPI_ISL_376800
**A/Vietnam/GS151050/2015**	EPI_ISL_213028	A/Aberystwyth/5868/2018	EPI_ISL_309450	A/Germany/7220/2018	EPI_ISL_376802
**A/Washington/77/2015**	EPI_ISL_213032	A/Neath/9065/2018	EPI_ISL_309457	A/Guam/7222/2018	EPI_ISL_376804
**A/Florida/09/2016**	EPI_ISL_213982	A/Swansea/9391/2018	EPI_ISL_309459	A/Italy/7225/2018	EPI_ISL_376807
**A/Hong Kong/98/2016**	EPI_ISL_213988	A/Cardiff/8328/2018	EPI_ISL_309468	A/New Jersey/7235/2018	EPI_ISL_376817
**A/Idaho/05/2016**	EPI_ISL_213989	A/Puerto Rico/16/2018	EPI_ISL_309541	A/New Jersey/7237/2018	EPI_ISL_376819
**A/New Mexico/32/2015**	EPI_ISL_213995	A/Arizona/28/2018	EPI_ISL_309542	A/New Mexico/7238/2018	EPI_ISL_376820
**A/St. Vincent And Grenadines/50/2015**	EPI_ISL_214008	A/South Dakota/35/2018	EPI_ISL_309543	A/New York/7240/2018	EPI_ISL_376822
**A/Tennessee/02/2016**	EPI_ISL_214551	A/Indiana/24/2018	EPI_ISL_309549	A/New York/7242/2018	EPI_ISL_376824
**A/Cambodia/1290/2015**	EPI_ISL_215251	A/Connecticut/26/2018	EPI_ISL_309556	A/New York/7243/2018	EPI_ISL_376825
**A/Minnesota/13/2016**	EPI_ISL_215263	A/Florida/51/2018	EPI_ISL_309565	A/New York/7244/2018	EPI_ISL_376826
**A/New Jersey/06/2016**	EPI_ISL_215265	A/Netherlands/10588/2018	EPI_ISL_309608	A/North Carolina/7245/2018	EPI_ISL_376827
**A/North Dakota/18/2016**	EPI_ISL_215266	A/Sundsvall/27/2017	EPI_ISL_310242	A/Ohio/7247/2018	EPI_ISL_376829
**A/Burkina Faso/1632/2015**	EPI_ISL_215615	A/Linkoping/5/2018	EPI_ISL_310250	A/Ohio/7248/2018	EPI_ISL_376830
**A/California/40/2016**	EPI_ISL_215623	A/Stockholm/8/2018	EPI_ISL_310255	A/Ohio/7250/2018	EPI_ISL_376832
**A/Cote D'Ivoire/2064/2015**	EPI_ISL_215634	A/Boras/1/2018	EPI_ISL_310259	A/Ohio/7251/2018	EPI_ISL_376833
**A/Cote D'Ivoire/2109/2015**	EPI_ISL_215635	A/Laos/F3523/2017	EPI_ISL_312769	A/South Carolina/7253/2018	EPI_ISL_376835
**A/Cote D'Ivoire/2110/2015**	EPI_ISL_215636	A/Delaware/30/2018	EPI_ISL_312803	A/South Carolina/7254/2018	EPI_ISL_376836
**A/Alingsas/1/2015**	EPI_ISL_215720	A/Virginia/45/2018	EPI_ISL_312813	A/South Dakota/7255/2018	EPI_ISL_376837
**A/Linkoping/2/2016**	EPI_ISL_215741	A/Afghanistan/0687/2017	EPI_ISL_312816	A/South Dakota/7257/2018	EPI_ISL_376839
**A/Sweden/46/2015**	EPI_ISL_215753	A/Florida/59/2018	EPI_ISL_312818	A/South Korea/7258/2018	EPI_ISL_376840
**A/California/47/2016**	EPI_ISL_215773	A/Florida/56/2018	EPI_ISL_312823	A/Utah/7260/2018	EPI_ISL_376842
**A/Ethiopia/1797/2016**	EPI_ISL_215779	A/Washington/90/2018	EPI_ISL_312826	A/Utah/7262/2018	EPI_ISL_376844
**A/Laos/1110/2015**	EPI_ISL_215784	A/Washington/92/2018	EPI_ISL_312827	A/Virginia/7263/2018	EPI_ISL_376845
**A/Maine/04/2016**	EPI_ISL_215793	A/Idaho/13/2018	EPI_ISL_312829	A/Virginia/7264/2018	EPI_ISL_376846
**A/Mississippi/02/2016**	EPI_ISL_215794	A/Utah/30/2018	EPI_ISL_312830	A/Washington/7266/2018	EPI_ISL_376848
**A/Washington/35/2016**	EPI_ISL_215803	A/Utah/31/2018	EPI_ISL_312831	A/Wyoming/7267/2018	EPI_ISL_376849
**A/Bangladesh/910006/2015**	EPI_ISL_219091	A/Minnesota/33/2018	EPI_ISL_312852	A/Bahrain/47/2019	EPI_ISL_377672
**A/Bangladesh/913012/2015**	EPI_ISL_219101	A/Pennsylvania/81/2018	EPI_ISL_312858	A/Burkina Faso/31/2019	EPI_ISL_377673
**A/Bangladesh/914007/2015**	EPI_ISL_219102	A/Maryland/40/2018	EPI_ISL_312861	A/Jamaica/8883/2019	EPI_ISL_377828
**A/Bangladesh/1010001/2015**	EPI_ISL_219109	A/Idaho/17/2018	EPI_ISL_312884	A/Jamaica/2063/2019	EPI_ISL_377832
**A/Bangladesh/1010002/2015**	EPI_ISL_219110	A/Kamchatka/414/2017	EPI_ISL_313430	A/Jamaica/0633/2019	EPI_ISL_377838
**A/Idaho/44/2015**	EPI_ISL_219134	A/Congo/007/2018	EPI_ISL_313517	A/Jamaica/4713/2019	EPI_ISL_377839
**A/Trinidad/187/2016**	EPI_ISL_219136	A/Laos/4957/2017	EPI_ISL_313522	A/Haiti/10028/2019	EPI_ISL_377842
**A/Arizona/32/2016**	EPI_ISL_219141	A/Afghanistan/1311/2018	EPI_ISL_313548	A/Madagascar/657/2019	EPI_ISL_377903

**A/California/41/2016**	EPI_ISL_219185	A/Iowa/52/2018	EPI_ISL_313551	A/Madagascar/326/2019	EPI_ISL_377904
**A/California/38/2016**	EPI_ISL_219188	A/Indiana/26/2018	EPI_ISL_313552	A/Jamaica/0352/2019	EPI_ISL_378022
**A/Rhode Island/11/2016**	EPI_ISL_219201	A/Rio Grande Do Sul/103/2018	EPI_ISL_313555	A/Nebraska/11/2019	EPI_ISL_378034
**A/California/137/2015**	EPI_ISL_219202	A/North Carolina/50/2017	EPI_ISL_313663	A/Haiti/40031/2019	EPI_ISL_378054
**A/Hawaii/14/2016**	EPI_ISL_219210	A/Illinois/31/2018	EPI_ISL_313684	A/Paraguay/3969/2019	EPI_ISL_378080
**A/Kentucky/07/2016**	EPI_ISL_219213	A/Illinois/25/2018	EPI_ISL_313687	A/Hawaii/51/2019	EPI_ISL_378089
**A/New Jersey/07/2016**	EPI_ISL_219214	A/Illinois/28/2018	EPI_ISL_313690	A/Lebanon/363/2019	EPI_ISL_378091
**A/Montana/25/2016**	EPI_ISL_219220	A/Pennsylvania/63/2018	EPI_ISL_313693	A/Lebanon/349/2019	EPI_ISL_378092
**A/North Dakota/20/2016**	EPI_ISL_219243	A/Virginia/37/2018	EPI_ISL_313705	A/Lebanon/303/2019	EPI_ISL_378094
**A/Kansas/17/2016**	EPI_ISL_219245	A/Illinois/40/2018	EPI_ISL_313720	A/Lebanon/299/2019	EPI_ISL_378095
**A/Wisconsin/37/2016**	EPI_ISL_219250	A/Virginia/41/2018	EPI_ISL_313725	A/Lebanon/407/2019	EPI_ISL_378098
**A/California/70/2016**	EPI_ISL_219253	A/Pennsylvania/69/2018	EPI_ISL_313729	A/Alagoas/280/2019	EPI_ISL_378108
**A/Nevada/21/2016**	EPI_ISL_219273	A/South Dakota/30/2018	EPI_ISL_313734	A/Honduras/4607/2019	EPI_ISL_378116
**A/Bangladesh/910004/2015**	EPI_ISL_220271	A/Florida/111/2017	EPI_ISL_313776	A/Honduras/4619/2019	EPI_ISL_378117
**A/Zambia/0002/2015**	EPI_ISL_220274	A/Florida/110/2017	EPI_ISL_313777	A/Honduras/4636/2019	EPI_ISL_378118
**A/Zambia/0102/2015**	EPI_ISL_220275	A/Illinois/54/2017	EPI_ISL_313778	A/Florida/7501/2018	EPI_ISL_378921
**A/Texas/66/2016**	EPI_ISL_220286	A/Missouri/38/2018	EPI_ISL_313785	A/Guam/7502/2018	EPI_ISL_378922
**A/Texas/77/2016**	EPI_ISL_220291	A/Iowa/39/2018	EPI_ISL_313792	A/Japan/7503/2018	EPI_ISL_378923
**A/Florida/43/2016**	EPI_ISL_220331	A/Neath/7855/2017	EPI_ISL_313954	A/Albania/9918/2019	EPI_ISL_380071
**A/Colorado/22/2016**	EPI_ISL_220336	A/Caldicot/0050/2018	EPI_ISL_313983	A/Albania/0080/2019	EPI_ISL_380072
**A/Louisiana/09/2016**	EPI_ISL_220343	A/Wisconsin/93/2018	EPI_ISL_314022	A/Kuwait/6365/2018	EPI_ISL_380077
**A/Oregon/10/2016**	EPI_ISL_220348	A/Louisiana/17/2018	EPI_ISL_314023	A/Guatemala/8828/2019	EPI_ISL_380084
**A/Utah/28/2016**	EPI_ISL_220357	A/Stockholm/18/2018	EPI_ISL_314058	A/Guatemala/93/2019	EPI_ISL_380087
**A/North Dakota/23/2016**	EPI_ISL_220362	A/Brazil/4145/2017	EPI_ISL_314340	A/Guatemala/123/2019	EPI_ISL_380091
**A/Illinois/11/2016**	EPI_ISL_220369	A/District Of Columbia/06/2017	EPI_ISL_314346	A/Guatemala/162/2019	EPI_ISL_380092
**A/New York/62/2016**	EPI_ISL_220374	A/New Jersey/35/2018	EPI_ISL_314349	A/Guatemala/174/2019	EPI_ISL_380093
**A/Lund/2/2016**	EPI_ISL_220448	A/District Of Columbia/04/2017	EPI_ISL_314371	A/Kuwait/6360/2018	EPI_ISL_380095
**A/Lund/3/2016**	EPI_ISL_220450	A/Lebanon/185/2018	EPI_ISL_314384	A/Uruguay/256/2019	EPI_ISL_380098
**A/Stockholm/75/2015**	EPI_ISL_220454	A/Virginia/53/2018	EPI_ISL_314385	A/Pennsylvania/899/2019	EPI_ISL_380399
**A/Stockholm/28/2016**	EPI_ISL_220472	A/Pakistan/918/2018	EPI_ISL_314397	A/Pennsylvania/904/2019	EPI_ISL_380400
**A/Stockholm/29/2016**	EPI_ISL_220477	A/Afghanistan/2251/2018	EPI_ISL_314407	A/Pennsylvania/923/2019	EPI_ISL_380414
**A/California/82/2016**	EPI_ISL_222009	A/Arizona/33/2018	EPI_ISL_314408	A/Pennsylvania/924/2019	EPI_ISL_380415
**A/Colorado/26/2016**	EPI_ISL_222012	A/Alaska/44/2018	EPI_ISL_314412	A/Michigan/317/2019	EPI_ISL_380451
**A/Connecticut/13/2016**	EPI_ISL_222015	A/Abu Dhabi/055/2018	EPI_ISL_315001	A/Michigan/333/2019	EPI_ISL_380466
**A/Florida/50/2016**	EPI_ISL_222020	A/Abu Dhabi/053/2018	EPI_ISL_315003	A/Michigan/341/2019	EPI_ISL_380467
**A/Hawaii/32/2016**	EPI_ISL_222027	A/Abu Dhabi/054/2018	EPI_ISL_315004	A/Wisconsin/416/2019	EPI_ISL_380496
**A/Hawaii/33/2016**	EPI_ISL_222028	A/Florida/65/2018	EPI_ISL_315031	A/Wisconsin/420/2019	EPI_ISL_380503
**A/Maryland/11/2016**	EPI_ISL_222037	A/Abu Dhabi/014/2018	EPI_ISL_315035	A/Wisconsin/477/2019	EPI_ISL_380531
**A/Massachusetts/28/2016**	EPI_ISL_222038	A/Jamaica/9936/2018	EPI_ISL_315037	A/Wisconsin/482/2019	EPI_ISL_380535
**A/New York/58/2016**	EPI_ISL_222056	A/Abu Dhabi/036/2018	EPI_ISL_315039	A/Wisconsin/489/2019	EPI_ISL_380547
**A/Georgia/45/2016**	EPI_ISL_223161	A/Abu Dhabi/015/2018	EPI_ISL_315048	A/Washington/504/2019	EPI_ISL_380603
**A/South Dakota/19/2016**	EPI_ISL_223181	A/Iquique/1308/2018	EPI_ISL_315089	A/Washington/491/2019	EPI_ISL_380607
**A/South Dakota/26/2016**	EPI_ISL_223184	A/Santiago/2775/2018	EPI_ISL_315090	A/Washington/540/2019	EPI_ISL_380628
**A/Texas/133/2016**	EPI_ISL_223187	A/Santiago/7340/2018	EPI_ISL_315095	A/Washington/551/2019	EPI_ISL_380632
**A/Wisconsin/62/2016**	EPI_ISL_223193	A/SHIGA/37/2017	EPI_ISL_315178	A/Washington/552/2019	EPI_ISL_380634
**A/Bangladesh/3010/2015**	EPI_ISL_223356	A/Bahrain/274/2018	EPI_ISL_315784	A/Washington/559/2019	EPI_ISL_380653
**A/Bangladesh/5012/2015**	EPI_ISL_223357	A/Bahrain/326/2018	EPI_ISL_315786	A/Washington/586/2019	EPI_ISL_380658
**A/Iowa/02/2016**	EPI_ISL_223358	A/Bahrain/329/2018	EPI_ISL_315787	A/Washington/587/2019	EPI_ISL_380659
**A/Minnesota/43/2016**	EPI_ISL_223360	A/Abu Dhabi/020/2018	EPI_ISL_316431	A/Washington/572/2019	EPI_ISL_380662
**A/California/12/2016**	EPI_ISL_223996	A/Hawaii/43/2018	EPI_ISL_316438	A/Washington/574/2019	EPI_ISL_380669
**A/Connecticut/06/2016**	EPI_ISL_224003	A/Abu Dhabi/024/2018	EPI_ISL_316440	A/Washington/575/2019	EPI_ISL_380670
**A/Delaware/10/2016**	EPI_ISL_224006	A/Brazil/128/2018	EPI_ISL_316767	A/Washington/616/2019	EPI_ISL_380729
**A/Iowa/20/2016**	EPI_ISL_224022	A/Philippines/0464/2018	EPI_ISL_319708	A/Washington/630/2019	EPI_ISL_380732
**A/Kansas/03/2016**	EPI_ISL_224023	A/Tanzania/2941/2018	EPI_ISL_319709	A/Washington/631/2019	EPI_ISL_380733
**A/Montana/43/2016**	EPI_ISL_224035	A/Philippines/0160/2018	EPI_ISL_319712	A/Washington/626/2019	EPI_ISL_380737
**A/New York/60/2016**	EPI_ISL_224044	A/Peru/1318/2018	EPI_ISL_319724	A/Washington/639/2019	EPI_ISL_380743
**A/Virginia/52/2016**	EPI_ISL_224062	A/Peru/0918/2018	EPI_ISL_319726	A/Wisconsin/506/2019	EPI_ISL_380755
**A/Virginia/55/2016**	EPI_ISL_224063	A/Peru/5918/2018	EPI_ISL_319728	A/Wisconsin/522/2019	EPI_ISL_380763
**A/Wyoming/22/2016**	EPI_ISL_224072	A/Peru/3018/2018	EPI_ISL_319729	A/Pennsylvania/874/2019	EPI_ISL_380771
**A/Mali/101 CH/2015**	EPI_ISL_224270	A/Ohio/23/2018	EPI_ISL_319730	A/Pennsylvania/876/2019	EPI_ISL_380773
**A/Uganda/274/2016**	EPI_ISL_225058	A/Argentina/70/2018	EPI_ISL_319732	A/Pennsylvania/884/2019	EPI_ISL_380780
**A/California/101/2016**	EPI_ISL_225079	A/Hong Kong/1106/2018	EPI_ISL_319737	A/Pennsylvania/886/2019	EPI_ISL_380783
**A/Talca/14380/2016**	EPI_ISL_225093	A/Hong Kong/1078/2018	EPI_ISL_319739	A/Wisconsin/536/2019	EPI_ISL_380786
**A/Connecticut/20/2016**	EPI_ISL_225098	A/Philippines/0060/2018	EPI_ISL_320280	A/Wisconsin/538/2019	EPI_ISL_380790
**A/New York/80/2016**	EPI_ISL_225106	A/Mexico/1707/2018	EPI_ISL_320282	A/Pennsylvania/937/2019	EPI_ISL_380793
**A/Florida/64/2016**	EPI_ISL_225110	A/Mexico/1641/2018	EPI_ISL_320284	A/Pennsylvania/925/2019	EPI_ISL_380795
**A/Utah/34/2016**	EPI_ISL_225120	A/Amazonas/153154-IEC/2018	EPI_ISL_320306	A/Wisconsin/547/2019	EPI_ISL_380796
**A/South Carolina/17/2016**	EPI_ISL_225128	A/Para/153301-IEC/2018	EPI_ISL_320309	A/Pennsylvania/939/2019	EPI_ISL_380797
**A/Washington/71/2016**	EPI_ISL_225131	A/Khakassia/249/2018	EPI_ISL_320324	A/Pennsylvania/943/2019	EPI_ISL_380801
**A/Alaska/19/2016**	EPI_ISL_225132	A/Ecuador/1938/2018	EPI_ISL_320331	A/Texas/134/2019	EPI_ISL_380825
**A/Wyoming/26/2016**	EPI_ISL_225141	A/Ecuador/4206/2018	EPI_ISL_320332	A/Texas/315/2019	EPI_ISL_380837
**A/Singapore/INFGP-16-0022/2016**	EPI_ISL_225902	A/Guatemala/0107/2018	EPI_ISL_320335	A/Texas/305/2019	EPI_ISL_380848
**A/Singapore/INFGP-16-0159/2016**	EPI_ISL_225906	A/Guatemala/55/2018	EPI_ISL_320339	A/Texas/196/2019	EPI_ISL_380885
**A/Singapore/INFGP-16-0192/2016**	EPI_ISL_225907	A/Philippines/0189/2018	EPI_ISL_320342	A/Texas/318/2019	EPI_ISL_380901
**A/Singapore/INFKK-16-0066/2016**	EPI_ISL_225911	A/Congo/422/2018	EPI_ISL_320345	A/Texas/326/2019	EPI_ISL_380906
**A/Singapore/INFGP-16-0422/2016**	EPI_ISL_225917	A/Congo/297/2018	EPI_ISL_320351	A/Texas/255/2019	EPI_ISL_380925
**A/Singapore/INFKK-16-0156/2016**	EPI_ISL_225920	A/New Jersey/38/2018	EPI_ISL_320740	A/Michigan/398/2019	EPI_ISL_380971
**A/Singapore/INFGP-16-0465/2016**	EPI_ISL_225923	A/New Hampshire/24/2018	EPI_ISL_320748	A/Michigan/399/2019	EPI_ISL_380972
**A/Singapore/INFSGH-16-0119/2016**	EPI_ISL_225925	A/Hawaii/45/2018	EPI_ISL_320749	A/Michigan/402/2019	EPI_ISL_380975
**A/Singapore/INFGP-16-0702/2016**	EPI_ISL_225928	A/Nebraska/08/2018	EPI_ISL_320753	A/Michigan/403/2019	EPI_ISL_380976
**A/Singapore/INFGP-16-0739/2016**	EPI_ISL_225929	A/Mozambique/2118/2018	EPI_ISL_320808	A/Michigan/404/2019	EPI_ISL_380977
**A/Singapore/INFEN-16-0172/2016**	EPI_ISL_225932	A/Wisconsin/95/2018	EPI_ISL_321202	A/Michigan/405/2019	EPI_ISL_380978
**A/Singapore/INFKK-16-0226/2016**	EPI_ISL_225934	A/Guatemala/144/2018	EPI_ISL_321215	A/Michigan/409/2019	EPI_ISL_380982
**A/Singapore/INFGP-16-0957/2016**	EPI_ISL_225935	A/Santiago/60852/2018	EPI_ISL_321218	A/Michigan/410/2019	EPI_ISL_380983
**A/Singapore/INFKK-16-0498/2016**	EPI_ISL_225937	A/Guyane/499/2018	EPI_ISL_321228	A/Michigan/411/2019	EPI_ISL_380984
**A/Singapore/INFIMH-16-0020/2016**	EPI_ISL_225941	A/Alagoas/615/2018	EPI_ISL_321237	A/Michigan/412/2019	EPI_ISL_380985
**A/Singapore/INFKK-16-0595/2016**	EPI_ISL_225944	A/Bahia/563/2018	EPI_ISL_321239	A/Arizona/9815/2019	EPI_ISL_381567
**A/Singapore/INFGP-16-1223/2016**	EPI_ISL_225952	A/Parana/817/2018	EPI_ISL_321248	A/North Dakota/9824/2019	EPI_ISL_381576

**A/Arizona/65/2016**	EPI_ISL_226791	A/Guatemala/138/2018	EPI_ISL_321251	A/Santiago/62358/2019	EPI_ISL_382025
**A/Washington/75/2016**	EPI_ISL_226796	A/Guyane/529/2018	EPI_ISL_321260	A/Indiana/34/2019	EPI_ISL_386844
**A/Moramanga/1942/2016**	EPI_ISL_230306	A/Anglesey/5245/2018	EPI_ISL_321565	A/Nakhonphanom/498/2019	EPI_ISL_386865
**A/Antananarivo/1951/2016**	EPI_ISL_230307	A/Caernarfon/8500/2018	EPI_ISL_321568	A/Concepcion/48458/2019	EPI_ISL_386960
**A/Antananarivo/1987/2016**	EPI_ISL_230308	A/Anglesey/8900/2018	EPI_ISL_321569	A/Santiago/60419/2019	EPI_ISL_386974
**A/Hong Kong/1704/2016**	EPI_ISL_230309	A/Wrexham/9057/2018	EPI_ISL_321570	A/Santiago/61209/2019	EPI_ISL_386979
**A/Hong Kong/1706/2016**	EPI_ISL_230311	A/Wrexham/9243/2018	EPI_ISL_321571	A/Vina_Del_Mar/64754/2019	EPI_ISL_386983
**A/Argentina/116/2016**	EPI_ISL_230315	A/Aberystwyth/2999/2018	EPI_ISL_321573	A/Germany/8437/2019	EPI_ISL_387217
**A/Texas/175/2016**	EPI_ISL_230316	A/Aberystwyth/6333/2018	EPI_ISL_321580	A/Illinois/8438/2019	EPI_ISL_387218
**A/Texas/174/2016**	EPI_ISL_230318	A/Paraguay/3019/2018	EPI_ISL_321864	A/Italy/8440/2019	EPI_ISL_387220
**A/Texas/170/2016**	EPI_ISL_230319	A/Paraguay/1722/2018	EPI_ISL_321876	A/Italy/8442/2019	EPI_ISL_387222
**A/Idaho/34/2016**	EPI_ISL_230323	A/Paraguay/3983/2018	EPI_ISL_321879	A/Italy/8444/2019	EPI_ISL_387224
**A/Texas/177/2016**	EPI_ISL_230331	A/Bolivia/01/2018	EPI_ISL_321896	A/Italy/8445/2019	EPI_ISL_387225
**A/Hawaii/46/2016**	EPI_ISL_230340	A/Guatemala/74/2018	EPI_ISL_321918	A/Italy/8446/2019	EPI_ISL_387226
**A/Hawaii/48/2016**	EPI_ISL_230341	A/Brazil/3223/2018	EPI_ISL_321929	A/Mississippi/8467/2019	EPI_ISL_387242
**A/California/118/2016**	EPI_ISL_230343	A/Brazil/0593/2018	EPI_ISL_321930	A/Montana/8469/2019	EPI_ISL_387244
**A/Minnesota/71/2016**	EPI_ISL_230347	A/Singapore/GP0077/2018	EPI_ISL_322071	A/New York/8475/2019	EPI_ISL_387250
**A/Utah/41/2016**	EPI_ISL_230349	A/Singapore/KK0078/2018	EPI_ISL_322076	A/New York/8478/2019	EPI_ISL_387252
**A/Oman/4726/2016**	EPI_ISL_230352	A/Singapore/KK0130/2018	EPI_ISL_322077	A/New York/8492/2019	EPI_ISL_387264
**A/Oman/4554/2016**	EPI_ISL_230353	A/Singapore/TT0043/2018	EPI_ISL_322080	A/New York/8494/2019	EPI_ISL_387266
**A/Oman/4909/2016**	EPI_ISL_230354	A/Singapore/GP0295/2018	EPI_ISL_322081	A/Ohio/8497/2019	EPI_ISL_387268
**A/Oman/4800/2016**	EPI_ISL_230355	A/Singapore/KK0269/2018	EPI_ISL_322088	A/Ohio/8498/2019	EPI_ISL_387269
**A/Peru/6116/2016**	EPI_ISL_230356	A/Singapore/KK0287/2018	EPI_ISL_322089	A/Ohio/8499/2019	EPI_ISL_387270
**A/Peru/5116/2016**	EPI_ISL_230357	A/Singapore/GP0270/2018	EPI_ISL_322094	A/Ohio/8501/2019	EPI_ISL_387272
**A/Peru/9016/2016**	EPI_ISL_230358	A/Singapore/GP0339/2018	EPI_ISL_322095	A/Ohio/8503/2019	EPI_ISL_387273
**A/Tanzania/2239/2016**	EPI_ISL_230372	A/Singapore/GP0508/2018	EPI_ISL_322098	A/South Dakota/8508/2019	EPI_ISL_387276
**A/Tanzania/2240/2016**	EPI_ISL_230373	A/Singapore/GP0623/2018	EPI_ISL_322100	A/Texas/8528/2019	EPI_ISL_387286
**A/Ethiopia/1978/2016**	EPI_ISL_230375	A/Singapore/GP1069/2018	EPI_ISL_322105	A/Texas/8542/2019	EPI_ISL_387296
**A/Texas/166/2016**	EPI_ISL_232103	A/Singapore/KK0422/2018	EPI_ISL_322110	A/Turkey/8543/2019	EPI_ISL_387297
**A/Honduras/7828/2016**	EPI_ISL_232105	A/Singapore/TT1765/2017	EPI_ISL_322111	A/Alaska/9321/2019	EPI_ISL_387424
**A/Georgia/NCDC1045/2016**	EPI_ISL_232106	A/Iquique/32413/2018	EPI_ISL_322137	A/Alaska/9322/2019	EPI_ISL_387425
**A/Bangkok/175/2016**	EPI_ISL_232109	A/Alto_Hospicio/32457/2018	EPI_ISL_322138	A/Arizona/9325/2019	EPI_ISL_387427
**A/Temuco/50233/2016**	EPI_ISL_232534	A/Iquique/48545/2018	EPI_ISL_322163	A/Arizona/9326/2019	EPI_ISL_387428
**A/Ecuador/1374/2016**	EPI_ISL_232542	A/Irkutsk/RII-1890S/2018	EPI_ISL_322422	A/California/9327/2019	EPI_ISL_387429
**A/Wisconsin/72/2016**	EPI_ISL_232551	A/Omsk/RII-1939S/2018	EPI_ISL_322425	A/Colorado/9328/2019	EPI_ISL_387430
**A/Ecuador/1372/2016**	EPI_ISL_232553	A/Sosnoviy_Bor/RII-2551S/2018	EPI_ISL_322427	A/Delaware/9334/2019	EPI_ISL_387433
**A/Utah/42/2016**	EPI_ISL_232567	A/Saint-Petersburg/RII-4213S/2018	EPI_ISL_322436	A/Delaware/9335/2019	EPI_ISL_387434
**A/Ecuador/2495/2016**	EPI_ISL_232986	A/Vologda/RII-4339S/2018	EPI_ISL_322438	A/England/9336/2019	EPI_ISL_387435
**A/Ecuador/2673/2016**	EPI_ISL_232988	A/Nizhni_Novgorod/RII-4452S/2018	EPI_ISL_322439	A/Florida/9340/2019	EPI_ISL_387438
**A/Ecuador/220/2016**	EPI_ISL_232989	A/Nizhni_Novgorod/RII-4454S/2018	EPI_ISL_322440	A/Georgia/9341/2019	EPI_ISL_387439
**A/Vermont/01/2016**	EPI_ISL_232990	A/Syktyvkar/RII-4625S/2018	EPI_ISL_322441	A/Germany/9344/2019	EPI_ISL_387441
**A/Guatemala/5261/2016**	EPI_ISL_232997	A/Noyabrsk/RII-4667S/2018	EPI_ISL_322442	A/Germany/9348/2019	EPI_ISL_387442
**A/Brazil/0293/2016**	EPI_ISL_232999	A/Ekaterinburg/RII-4774S/2018	EPI_ISL_322446	A/Germany/9353/2019	EPI_ISL_387443
**A/Stockholm/30/2016**	EPI_ISL_233407	A/Jamaica/1208/2018	EPI_ISL_322932	A/Germany/9356/2019	EPI_ISL_387446
**A/Guadeloupe/7129/2016**	EPI_ISL_233431	A/Jamaica/7492/2018	EPI_ISL_322941	A/Germany/9358/2019	EPI_ISL_387448
**A/Hawaii/60/2016**	EPI_ISL_233434	A/Colombia/0291/2018	EPI_ISL_322951	A/Germany/9359/2019	EPI_ISL_387449
**A/Guatemala/4392/2016**	EPI_ISL_233438	A/Colombia/0113/2018	EPI_ISL_322953	A/Germany/9360/2019	EPI_ISL_387450
**A/Arizona/67/2016**	EPI_ISL_233441	A/Brazil/5557/2018	EPI_ISL_322967	A/Illinois/9361/2019	EPI_ISL_387451
**A/Martinique/2225/2016**	EPI_ISL_233442	A/Brazil/1767/2018	EPI_ISL_322968	A/Italy/9363/2019	EPI_ISL_387452
**A/Hawaii/62/2016**	EPI_ISL_233445	A/Cardiff/8857/2018	EPI_ISL_327399	A/Italy/9364/2019	EPI_ISL_387453
**A/California/120/2016**	EPI_ISL_233447	A/Cardiff/9945/2018	EPI_ISL_327694	A/Italy/9367/2019	EPI_ISL_387454
**A/Bangladesh/1008/2016**	EPI_ISL_233449	A/New Jersey/41/2018	EPI_ISL_329794	A/Mississippi/9371/2019	EPI_ISL_387457
**A/Bangladesh/5009/2016**	EPI_ISL_233451	A/Lopburi/1262/2018	EPI_ISL_329802	A/Missouri/9374/2019	EPI_ISL_387460
**A/Guatemala/6102/2016**	EPI_ISL_233455	A/New York/41/2018	EPI_ISL_329817	A/Montana/9376/2019	EPI_ISL_387461
**A/Texas/179/2016**	EPI_ISL_233456	A/Minas Gerais/444/2018	EPI_ISL_329821	A/New Jersey/9382/2019	EPI_ISL_387466
**A/Texas/178/2016**	EPI_ISL_233457	A/Michigan/385/2018	EPI_ISL_329830	A/New Jersey/9383/2019	EPI_ISL_387467
**A/Bangladesh/3022/2016**	EPI_ISL_233466	A/Bolivia/0829/2018	EPI_ISL_329839	A/New Jersey/9385/2019	EPI_ISL_387469
**A/Bangladesh/402/2016**	EPI_ISL_233468	A/Bolivia/0703/2018	EPI_ISL_329844	A/New Jersey/9386/2019	EPI_ISL_387470
**A/Puerto Rico/09/2016**	EPI_ISL_233471	A/Brazil/9555/2018	EPI_ISL_329846	A/New York/9390/2019	EPI_ISL_387474
**A/Bangladesh/3075/2016**	EPI_ISL_233472	A/Jamaica/0686/2018	EPI_ISL_329851	A/New York/9391/2019	EPI_ISL_387475
**A/Stockholm/32/2016**	EPI_ISL_234034	A/Brazil/6494/2018	EPI_ISL_329855	A/New York/9393/2019	EPI_ISL_387476
**A/Colombia/9773/2016**	EPI_ISL_234038	A/Brazil/4623/2018	EPI_ISL_329859	A/New York/9394/2019	EPI_ISL_387477
**A/Hong Kong/2298/2016**	EPI_ISL_234041	A/Brazil/8415/2018	EPI_ISL_329860	A/New York/9395/2019	EPI_ISL_387478
**A/Iowa/23/2016**	EPI_ISL_234047	A/Brazil/8306/2018	EPI_ISL_329861	A/North Dakota/9397/2019	EPI_ISL_387480
**A/California/122/2016**	EPI_ISL_234984	A/Brazil/8380/2018	EPI_ISL_329862	A/North Dakota/9398/2019	EPI_ISL_387481
**A/Ohio/31/2016**	EPI_ISL_234991	A/Bahrain/408/2018	EPI_ISL_330011	A/Ohio/9403/2019	EPI_ISL_387485
**A/North Carolina/51/2016**	EPI_ISL_235071	A/Minnesota/42/2018	EPI_ISL_330016	A/Ohio/9404/2019	EPI_ISL_387486
**A/Lebanon/998/2016**	EPI_ISL_235078	A/Hawaii/49/2018	EPI_ISL_330018	A/Ohio/9406/2019	EPI_ISL_387487
**A/Congo/0941/2016**	EPI_ISL_235091	A/Bolivia/0898/2018	EPI_ISL_330035	A/Ohio/9409/2019	EPI_ISL_387490
**A/Congo/0947/2016**	EPI_ISL_235092	A/Parana/1046/2018	EPI_ISL_330246	A/Ohio/9410/2019	EPI_ISL_387491
**A/Uganda/5535/2016**	EPI_ISL_235101	A/Guatemala/116/2018	EPI_ISL_330247	A/Ohio/9412/2019	EPI_ISL_387493
**A/Uganda/0182/2016**	EPI_ISL_235104	A/Guatemala/183/2018	EPI_ISL_330252	A/Ohio/9415/2019	EPI_ISL_387496
**A/Rwanda/572/2016**	EPI_ISL_235537	A/Guatemala/143/2018	EPI_ISL_330254	A/South Carolina/9422/2019	EPI_ISL_387500
**A/New York/WC-LVD-14-069/2014**	EPI_ISL_235616	A/Brazil/8178/2018	EPI_ISL_330269	A/Texas/9423/2019	EPI_ISL_387501
**A/New York/WC-LVD-14-081/2014**	EPI_ISL_235627	A/Brazil/8425/2018	EPI_ISL_330275	A/Texas/9434/2019	EPI_ISL_387510
**A/New York/WC-LVD-14-083/2014**	EPI_ISL_235633	A/Brazil/7180/2018	EPI_ISL_330276	A/Utah/9439/2019	EPI_ISL_387512
**A/New York/WC-LVD-14-084/2014**	EPI_ISL_235634	A/Brazil/4139/2018	EPI_ISL_330282	A/Utah/9440/2019	EPI_ISL_387513
**A/New York/WC-LVD-15-005/2015**	EPI_ISL_235637	A/Uruguay/610/2018	EPI_ISL_330285	A/Washington/9442/2019	EPI_ISL_387515
**A/New York/WC-LVD-15-012/2015**	EPI_ISL_235644	A/Florida/83/2018	EPI_ISL_330289	A/Washington/9448/2019	EPI_ISL_387516
**A/New York/WC-LVD-15-032/2015**	EPI_ISL_235668	A/Burkina Faso/827/2018	EPI_ISL_330475	A/Washington/9449/2019	EPI_ISL_387517
**A/New York/WC-LVD-15-033/2015**	EPI_ISL_235669	A/Minnesota/48/2018	EPI_ISL_330501	A/Washington/9450/2019	EPI_ISL_387518
**A/New York/WC-LVD-15-037/2015**	EPI_ISL_235672	A/Michigan/394/2018	EPI_ISL_330503	A/Argentina/911/2019	EPI_ISL_390002
**A/New York/WC-LVD-15-040/2015**	EPI_ISL_235675	A/Maldives/781/2018	EPI_ISL_330952	A/Argentina/841/2019	EPI_ISL_390004
**A/New York/WC-LVD-15-042/2015**	EPI_ISL_235677	A/Maldives/941/2018	EPI_ISL_330959	A/Argentina/820/2019	EPI_ISL_390005
**A/New York/WC-LVD-15-044/2015**	EPI_ISL_235679	A/Bolivia/1914/2018	EPI_ISL_331050	A/Abu Dhabi/053/2019	EPI_ISL_390009
**A/New York/WC-LVD-15-048/2015**	EPI_ISL_235687	A/Lebanon/427/2018	EPI_ISL_331053	A/Hong Kong/3053/2019	EPI_ISL_390013
**A/New York/WC-LVD-14-099/2014**	EPI_ISL_235689	A/South Africa/6922/2018	EPI_ISL_331067	A/Hong Kong/3047/2019	EPI_ISL_390015
**A/New York/WC-LVD-14-100/2014**	EPI_ISL_235690	A/Pakistan/1193/2018	EPI_ISL_331134	A/Hong Kong/3052/2019	EPI_ISL_390016

**A/New York/WC-LVD-15-051/2015**	EPI_ISL_235691	A/Pakistan/1152/2018	EPI_ISL_331135	A/Hong Kong/3043/2019	EPI_ISL_390017
**A/New York/WC-LVD-14-102/0214**	EPI_ISL_235692	A/Texas/132/2018	EPI_ISL_331136	A/Costa Rica/8855/2019	EPI_ISL_390034
**A/New York/WC-LVD-15-052/2015**	EPI_ISL_235693	A/New York/46/2018	EPI_ISL_331400	A/Florida/85/2019	EPI_ISL_390036
**A/New York/WC-LVD-15-053/2015**	EPI_ISL_235694	A/Pakistan/1601/2018	EPI_ISL_331409	A/Florida/86/2019	EPI_ISL_390037
**A/New York/98/2016**	EPI_ISL_237295	A/Pakistan/1424/2018	EPI_ISL_331411	A/Minnesota/50/2019	EPI_ISL_390088
**A/Hawaii/77/2016**	EPI_ISL_237311	A/Pakistan/1331/2018	EPI_ISL_331412	A/Argentina/1532/2019	EPI_ISL_390090
**A/Florida/79/2016**	EPI_ISL_237329	A/Alaska/54/2018	EPI_ISL_331864	A/Argentina/1468/2019	EPI_ISL_390092
**A/North Carolina/54/2016**	EPI_ISL_237341	A/Guatemala/148/2018	EPI_ISL_331872	A/Argentina/1449/2019	EPI_ISL_390093
**A/Illinois/45/2016**	EPI_ISL_237344	A/New Hampshire/27/2018	EPI_ISL_331878	A/Argentina/1434/2019	EPI_ISL_390096
**A/Nebraska/23/2016**	EPI_ISL_237352	A/Bhutan/740/2018	EPI_ISL_331903	A/Argentina/1422/2019	EPI_ISL_390097
**A/Guam/3224/2015**	EPI_ISL_237357	A/Massachusetts/26/2018	EPI_ISL_331915	A/Argentina/1356/2019	EPI_ISL_390098
**A/Mozambique/207/2016**	EPI_ISL_237366	A/California/71/2018	EPI_ISL_331917	A/Costa Rica/7314/2019	EPI_ISL_390107
**A/California/135/2016**	EPI_ISL_237437	A/Arizona/44/2018	EPI_ISL_331918	A/Costa Rica/8776/2019	EPI_ISL_390112
**A/South Africa/5233/2016**	EPI_ISL_237483	A/Bolivia/3899/2018	EPI_ISL_332174	A/Hong Kong/3059/2019	EPI_ISL_390113
**A/Kungalv/1/2016**	EPI_ISL_237508	A/Espirito Santo/737/2018	EPI_ISL_332311	A/Kenya/6516/2019	EPI_ISL_390124
**A/Costa Rica/0014/2016**	EPI_ISL_238646	A/Utah/42/2018	EPI_ISL_332549	A/Kenya/2256/2019	EPI_ISL_390126
**A/Costa Rica/5828/2016**	EPI_ISL_238647	A/Delaware/40/2018	EPI_ISL_332552	A/Argentina/14353/2019	EPI_ISL_390135
**A/Costa Rica/2536/2016**	EPI_ISL_238648	A/Abu Dhabi/102/2018	EPI_ISL_333435	A/Argentina/14372/2019	EPI_ISL_390136
**A/Bangladesh/3001/2016**	EPI_ISL_238649	A/Abu Dhabi/101/2018	EPI_ISL_333463	A/Bangladesh/1190603045/2019	EPI_ISL_390157
**A/Peru/61016/2016**	EPI_ISL_238654	A/Washington/190/2018	EPI_ISL_333738	A/Umea/1/2019	EPI_ISL_391067
**A/California/143/2016**	EPI_ISL_238712	A/Minnesota/58/2018	EPI_ISL_333748	A/Norrkoping/1/2019	EPI_ISL_391084
**A/Wisconsin/89/2016**	EPI_ISL_238714	A/Maine/36/2018	EPI_ISL_333760	A/Pakistan/22/2019	EPI_ISL_391101
**A/Alabama/14/2016**	EPI_ISL_238719	A/Kentucky/32/2018	EPI_ISL_334110	A/Pakistan/78/2019	EPI_ISL_391102
**A/Virginia/61/2016**	EPI_ISL_238721	A/Puerto Rico/30/2018	EPI_ISL_334116	A/Pakistan/83/2019	EPI_ISL_391103
**A/Tennessee/28/2016**	EPI_ISL_238733	A/Virginia/58/2018	EPI_ISL_334126	A/Laos/588/2019	EPI_ISL_391106
**A/California/149/2016**	EPI_ISL_238740	A/Bangladesh/15205/2018	EPI_ISL_334154	A/Pakistan/111/2019	EPI_ISL_391107
**A/Singapore/TT0846/2016**	EPI_ISL_239134	A/Kenya/162/2018	EPI_ISL_335646	A/Laos/631/2019	EPI_ISL_391110
**A/Michigan/110/2016**	EPI_ISL_239191	A/Kenya/163/2018	EPI_ISL_335647	A/Pakistan/1971/2019	EPI_ISL_391113
**A/Minnesota/79/2016**	EPI_ISL_239194	A/Kenya/164/2018	EPI_ISL_335656	A/Laos/1789/2019	EPI_ISL_391185
**A/Montana/58/2016**	EPI_ISL_239196	A/Michigan/414/2018	EPI_ISL_335659	A/Florida/90/2019	EPI_ISL_391188
**A/South Dakota/28/2016**	EPI_ISL_239212	A/Maine/37/2018	EPI_ISL_335667	A/Hawaii/59/2019	EPI_ISL_391367
**A/Utah/46/2016**	EPI_ISL_239216	A/Connecticut/39/2018	EPI_ISL_335678	A/Maryland/31/2019	EPI_ISL_391370
**A/Singapore/IMH0029/2016**	EPI_ISL_239250	A/Harrow/5668/2019	EPI_ISL_335732	A/Singapore/GP2220/2018	EPI_ISL_391576
**A/Singapore/TT1150/2016**	EPI_ISL_239279	A/Sweden/8/2019	EPI_ISL_336152	A/Singapore/GP2224/2018	EPI_ISL_391577
**A/Singapore/TT1156/2016**	EPI_ISL_239280	A/Orebro/1/2019	EPI_ISL_336154	A/Singapore/KK0027/2019	EPI_ISL_391593
**A/Singapore/MOH0107/2016**	EPI_ISL_239325	A/Maine/46/2018	EPI_ISL_336522	A/Singapore/KK0061/2019	EPI_ISL_391594
**A/Linkoping/4/2016**	EPI_ISL_239532	A/Lebanon/596/2018	EPI_ISL_336537	A/Singapore/GP0566/2019	EPI_ISL_391670
**A/Karlstad/5/2016**	EPI_ISL_239534	A/Louisiana/27/2018	EPI_ISL_336565	A/Singapore/GP0696/2019	EPI_ISL_391672
**A/Sweden/63/2016**	EPI_ISL_239545	A/Iowa/76/2018	EPI_ISL_336572	A/Mali/038/2019	EPI_ISL_392519
**A/Connecticut/36/2016**	EPI_ISL_239683	A/Abu Dhabi/112/2018	EPI_ISL_336696	A/Mali/010/2019	EPI_ISL_392525
**A/Pennsylvania/97/2016**	EPI_ISL_239721	A/Saint-Petersburg/RII-577S/2019	EPI_ISL_337110	A/Sao Paulo/1198850-IAL/2019	EPI_ISL_392527
**A/Vermont/33/2016**	EPI_ISL_239728	A/Michigan/418/2018	EPI_ISL_338602	A/Sao Paulo/1201946-IAL/2019	EPI_ISL_392532
**A/Minnesota/80/2016**	EPI_ISL_240177	A/Kuwait/6415/2018	EPI_ISL_338611	A/Barueri/1216473-IAL/2019	EPI_ISL_392535
**A/Hawaii/96/2016**	EPI_ISL_240197	A/Kuwait/6419/2018	EPI_ISL_338612	A/Sao Paulo/1265906-IAL/2019	EPI_ISL_392541
**A/North Carolina/62/2016**	EPI_ISL_240205	A/Kuwait/6430/2018	EPI_ISL_338615	A/Brasilandia/588973-IAL/2019	EPI_ISL_392545
**A/Illinois/50/2016**	EPI_ISL_240210	A/Kuwait/6435/2018	EPI_ISL_338618	A/Brasilia/2068302-IAL/2019	EPI_ISL_392548
**A/Singapore/GP2545/2016**	EPI_ISL_240771	A/Guatemala/974/2018	EPI_ISL_338626	A/Florida/94/2019	EPI_ISL_392556
**A/Singapore/GP2497/2016**	EPI_ISL_240773	A/Guatemala/284/2018	EPI_ISL_338629	A/Peru/586619/2019	EPI_ISL_392560
**A/Singapore/GP2539/2016**	EPI_ISL_240774	A/Guatemala/01/2018	EPI_ISL_338630	A/Peru/5019/2019	EPI_ISL_392562
**A/Singapore/EN0934/2016**	EPI_ISL_240775	A/Bulgaria/1376/2018	EPI_ISL_338632	A/Peru/3819/2019	EPI_ISL_392565
**A/Singapore/GP2594/2016**	EPI_ISL_240786	A/Costa Rica/1625/2018	EPI_ISL_338723	A/Lebanon/120/2019	EPI_ISL_392566
**A/Singapore/GP2642/2016**	EPI_ISL_240796	A/Costa Rica/2948/2018	EPI_ISL_338727	A/Anapolis/487395-IAL/2019	EPI_ISL_392567
**A/Singapore/GP2605/2016**	EPI_ISL_240799	A/California/08/2019	EPI_ISL_339679	A/Louisiana/39/2019	EPI_ISL_392568
**A/California/182/2016**	EPI_ISL_241597	A/New York/61/2018	EPI_ISL_339709	A/New York/45/2019	EPI_ISL_392577
**A/Mississippi/19/2016**	EPI_ISL_241632	A/Punta Arenas/108822/2018	EPI_ISL_339719	A/Hawaii/61/2019	EPI_ISL_392578
**A/Mississippi/20/2016**	EPI_ISL_241633	A/West Virginia/01/2019	EPI_ISL_339723	A/Wisconsin/562/2019	EPI_ISL_392584
**A/Sweden/86/2016**	EPI_ISL_241933	A/Pennsylvania/02/2019	EPI_ISL_339729	A/Mali/054/2019	EPI_ISL_393575
**A/Netherlands/498/2017**	EPI_ISL_242386	A/Delaware/07/2019	EPI_ISL_339751	A/Mali/9054/2019	EPI_ISL_393580
**A/Netherlands/499/2017**	EPI_ISL_242387	A/California/11/2019	EPI_ISL_339834	A/Mali/9043/2019	EPI_ISL_393583
**A/Saint-Petersburg/RII855S/2016**	EPI_ISL_242600	A/Hawaii/78/2018	EPI_ISL_339851	A/Peru/9519/2019	EPI_ISL_393586
**A/Nizhny Tagil/RII322S/2016**	EPI_ISL_242610	A/Eskilstuna/7/2018	EPI_ISL_340278	A/Mali/064/2019	EPI_ISL_393588
**A/Vologda/RII506S/2016**	EPI_ISL_242629	A/Netherlands/10050/2019	EPI_ISL_340298	A/Peru/5419/2019	EPI_ISL_393591
**A/Pskov/RII739S/2016**	EPI_ISL_242644	A/Singapore/KK0699/2018	EPI_ISL_340479	A/Peru/2419/2019	EPI_ISL_393592
**A/Volgograd/RII759S/2016**	EPI_ISL_242648	A/Singapore/GP1479/2018	EPI_ISL_340483	A/Peru/3419/2019	EPI_ISL_393593
**A/Simpheropol/RII837S/2016**	EPI_ISL_242658	A/Singapore/GP1616/2018	EPI_ISL_340486	A/Peru/7519/2019	EPI_ISL_393594
**A/Colombia/1368/2016**	EPI_ISL_242695	A/Singapore/KK1045/2018	EPI_ISL_340489	A/Mali/037/2019	EPI_ISL_393595
**A/Colombia/1408/2016**	EPI_ISL_242697	A/Singapore/GP2022/2018	EPI_ISL_340494	A/Peru/2891/2019	EPI_ISL_393597
**A/Illinois/63/2016**	EPI_ISL_242744	A/Massachusetts/37/2018	EPI_ISL_341026	A/Mali/9049/2019	EPI_ISL_393598
**A/California/197/2016**	EPI_ISL_242749	A/Idaho/38/2018	EPI_ISL_341115	A/Bolivia/977/2019	EPI_ISL_393789
**A/CastillaLaMancha/3399/2016**	EPI_ISL_242976	A/Kuwait/6363/2018	EPI_ISL_341172	A/Arizona/37/2019	EPI_ISL_394877
**A/Florida/108/2016**	EPI_ISL_244652	A/Kuwait/6359/2018	EPI_ISL_341183	A/Arizona/35/2019	EPI_ISL_394878
**A/Oklahoma/02/2017**	EPI_ISL_244724	A/Kuwait/6355/2018	EPI_ISL_341185	A/Michigan/444/2019	EPI_ISL_394889
**A/Rhode Island/30/2016**	EPI_ISL_244751	A/Kuwait/6356/2018	EPI_ISL_341186	A/Maryland/32/2019	EPI_ISL_394892
**A/Rhode Island/31/2016**	EPI_ISL_244752	A/Valdiva/100553/2018	EPI_ISL_341189	A/Hawaii/62/2019	EPI_ISL_394894
**A/Massachusetts/45/2016**	EPI_ISL_244762	A/Netherlands/10086/2019	EPI_ISL_342122	A/Michigan/439/2019	EPI_ISL_394899
**A/Maryland/30/2016**	EPI_ISL_244765	A/California/127/2018	EPI_ISL_342232	A/Togo/856/2019	EPI_ISL_394904
**A/Maryland/31/2016**	EPI_ISL_244767	A/Eskilstuna/2/2019	EPI_ISL_343370	A/Bolivia/996/2019	EPI_ISL_394920
**A/Brazil/0809/2016**	EPI_ISL_244823	A/Umea/1/2018	EPI_ISL_343371	A/Bolivia/1013/2019	EPI_ISL_394921
**A/Bulgaria/1213/2016**	EPI_ISL_244860	A/Skovde/1/2019	EPI_ISL_343376	A/Togo/827/2019	EPI_ISL_394923
**A/Bulgaria/1189/2016**	EPI_ISL_244863	A/Sweden/24/2019	EPI_ISL_343377	A/Togo/848/2019	EPI_ISL_394924
**A/Bulgaria/1181/2016**	EPI_ISL_244865	A/Singapore/GP2173/2018	EPI_ISL_344188	A/Kenya/136/2019	EPI_ISL_394950
**A/Bulgaria/1162/2016**	EPI_ISL_244868	A/Singapore/GP2191/2018	EPI_ISL_344191	A/Suriname/0835/2019	EPI_ISL_394953
**A/Philippines/0631/2016**	EPI_ISL_244879	A/Singapore/MOH0161/2018	EPI_ISL_344195	A/Bolivia/1107/2019	EPI_ISL_395100
**A/Guatemala/506/2016**	EPI_ISL_244882	A/Delaware/12/2019	EPI_ISL_344675	A/Bolivia/1103/2019	EPI_ISL_395102
**A/Guatemala/6455/2016**	EPI_ISL_244884	A/Bolivia/02325/2018	EPI_ISL_346039	A/Bolivia/1232/2019	EPI_ISL_395114
**A/Netherlands/756/2017**	EPI_ISL_247417	A/Bolivia/2203/2018	EPI_ISL_346040	A/Bolivia/1116/2019	EPI_ISL_395127
**A/Netherlands/760/2017**	EPI_ISL_247419	A/Ecuador/4125/2019	EPI_ISL_346047	A/Bangladesh/3190704014/2019	EPI_ISL_395138
**A/Roraima/143199-IEC/2016**	EPI_ISL_247961	A/Congo/734/2018	EPI_ISL_346055	A/Bolivia/1410/2019	EPI_ISL_395257

**A/Paraiba/143404-IEC/2016**	EPI_ISL_247962	A/Peru/1418/2018	EPI_ISL_346100	A/Bolivia/1433/2019	EPI_ISL_395258
**A/Roraima/144714-IEC/2016**	EPI_ISL_247965	A/Colombia/2604/2018	EPI_ISL_346107	A/Bangladesh/1001/2019	EPI_ISL_395396
**A/Guatemala/4794/2016**	EPI_ISL_247966	A/Kuwait/821/2019	EPI_ISL_346113	A/Bangladesh/8029/2019	EPI_ISL_395416
**A/Guatemala/4800/2016**	EPI_ISL_247967	A/Peru/0418/2018	EPI_ISL_346121	A/Oregon/26/2019	EPI_ISL_395427
**A/Illinois/08/2017**	EPI_ISL_248003	A/Kuwait/713/2019	EPI_ISL_346235	A/Iowa/47/2019	EPI_ISL_395430
**A/Connecticut/44/2016**	EPI_ISL_248009	A/Singapore/EN0025/2019	EPI_ISL_346398	A/Louisiana/43/2019	EPI_ISL_395432
**A/Illinois/05/2017**	EPI_ISL_248088	A/Singapore/EN0149/2019	EPI_ISL_346399	A/Alaska/48/2019	EPI_ISL_395434
**A/Pennsylvania/118/2016**	EPI_ISL_248099	A/Singapore/KK1680/2018	EPI_ISL_346446	A/Michigan/446/2019	EPI_ISL_395440
**A/New Hampshire/04/2017**	EPI_ISL_248108	A/Singapore/NTF0071/2018	EPI_ISL_346449	A/Bangladesh/1190711015/2019	EPI_ISL_395449
**A/South Dakota/02/2017**	EPI_ISL_248118	A/Vina_Del_Mar/52623/2018	EPI_ISL_346977	A/Montana/49/2019	EPI_ISL_396991
**A/South Dakota/33/2016**	EPI_ISL_248122	A/Concepcion/73626/2018	EPI_ISL_347047	A/Hawaii/65/2019	EPI_ISL_397003
**A/Oregon/04/2017**	EPI_ISL_248137	A/Santiago/79234/2018	EPI_ISL_347068	A/Peru/4619/2019	EPI_ISL_397067
**A/Wyoming/03/2017**	EPI_ISL_248138	A/Santiago/79400/2018	EPI_ISL_347069	A/North Carolina/29/2019	EPI_ISL_397174
**A/Nebraska/01/2017**	EPI_ISL_248140	A/Iquique/81059/2018	EPI_ISL_347075	A/New Jersey/34/2019	EPI_ISL_397180
**A/Connecticut/03/2017**	EPI_ISL_248149	A/Puerto_Montt/81522/2018	EPI_ISL_347079	A/Delaware/49/2019	EPI_ISL_397186
**A/Connecticut/04/2017**	EPI_ISL_248150	A/Puerto_Montt/84724/2018	EPI_ISL_347083	A/New York/47/2019	EPI_ISL_397188
**A/Rhode Island/01/2017**	EPI_ISL_248151	A/Concepcion/87275/2018	EPI_ISL_347086	A/Pennsylvania/1022/2019	EPI_ISL_397191
**A/Arkansas/03/2017**	EPI_ISL_248154	A/Temuco/79791/2018	EPI_ISL_347103	A/Florida/107/2019	EPI_ISL_397193
**A/Alabama/04/2017**	EPI_ISL_248284	A/Temuco/85881/2018	EPI_ISL_347104	A/Honduras/5229/2019	EPI_ISL_397194
**A/Minnesota/07/2017**	EPI_ISL_248286	A/Linkoping/6/2019	EPI_ISL_347795	A/Nigeria/3273/2019	EPI_ISL_397199
**A/Maryland/04/2017**	EPI_ISL_248296	A/Sweden/29/2019	EPI_ISL_347799	A/Bahia/736/2019	EPI_ISL_397211
**A/Michigan/05/2017**	EPI_ISL_248378	A/Bangladesh/876/2018	EPI_ISL_347909	A/Bahia/744/2019	EPI_ISL_397212
**A/Belgorod/1/2016**	EPI_ISL_249014	A/Ecuador/1529/2019	EPI_ISL_347935	A/Massachusetts/38/2019	EPI_ISL_397214
**A/California/12/2017**	EPI_ISL_249016	A/Hong Kong/45/2019	EPI_ISL_347938	A/North Dakota/33/2019	EPI_ISL_397219
**A/Costa Rica/8964/2016**	EPI_ISL_249030	A/Congo/717/2018	EPI_ISL_347944	A/Nigeria/3769/2019	EPI_ISL_398311
**A/Delaware/03/2017**	EPI_ISL_249031	A/Congo/009/2019	EPI_ISL_347952	A/Nigeria/3775/2019	EPI_ISL_398315
**A/Hawaii/106/2016**	EPI_ISL_249034	A/California/57/2019	EPI_ISL_347964	A/Nicaragua/1337/2019	EPI_ISL_398327
**A/Hong Kong/122/2017**	EPI_ISL_249037	A/California/79/2019	EPI_ISL_347992	A/Bangladesh/1190714031/2019	EPI_ISL_398330
**A/Hong Kong/123/2017**	EPI_ISL_249038	A/Mississippi/14/2019	EPI_ISL_348159	A/Honduras/5367/2019	EPI_ISL_398334
**A/Hong Kong/237/2017**	EPI_ISL_249040	A/Congo/002/2019	EPI_ISL_348160	A/Guatemala/227/2019	EPI_ISL_398335
**A/Voronezh/1/2016**	EPI_ISL_249069	A/Venezuela/22/2018	EPI_ISL_348199	A/Parana/908/2019	EPI_ISL_398337
**A/Netherlands/1994/2017**	EPI_ISL_249661	A/Arkansas/21/2019	EPI_ISL_348230	A/Bangladesh/1190810027/2019	EPI_ISL_398347
**A/Netherlands/1996/2017**	EPI_ISL_249662	A/Texas/111/2019	EPI_ISL_348240	A/Bangladesh/1190810023/2019	EPI_ISL_398349
**A/Netherlands/2009/2017**	EPI_ISL_249667	A/Ecuador/506/2019	EPI_ISL_349690	A/Bangladesh/1190814014/2019	EPI_ISL_398354
**A/Guadeloupe/567/2016**	EPI_ISL_249967	A/Kuwait/064/2019	EPI_ISL_349764	A/Bangladesh/1190804009/2019	EPI_ISL_398358
**A/Guyane/001/2017**	EPI_ISL_249975	A/California/87/2019	EPI_ISL_349955	A/Bangladesh/3190814007/2019	EPI_ISL_398359
**A/Connecticut/06/2017**	EPI_ISL_250005	A/California/94/2019	EPI_ISL_349959	A/Bangladesh/3190804004/2019	EPI_ISL_398368
**A/Oregon/06/2017**	EPI_ISL_250018	A/Pakistan/542/2019	EPI_ISL_350068	A/Bangladesh/1190713044/2019	EPI_ISL_398370
**A/New Hampshire/06/2017**	EPI_ISL_250030	A/Pakistan/214/2019	EPI_ISL_350069	A/Bangladesh/4052/2019	EPI_ISL_398376
**A/Maine/09/2017**	EPI_ISL_250034	A/Lebanon/231/2019	EPI_ISL_350084	A/Bangladesh/1190714021/2019	EPI_ISL_398399
**A/Arizona/05/2017**	EPI_ISL_250044	A/Lebanon/207/2019	EPI_ISL_350085	A/Bangladesh/031707/2019	EPI_ISL_398529
**A/Wyoming/06/2017**	EPI_ISL_250046	A/Singapore/KK0658/2018	EPI_ISL_350557	A/Nigeria/3280/2019	EPI_ISL_398532
**A/North Carolina/05/2017**	EPI_ISL_250055	A/Singapore/GP1425/2018	EPI_ISL_350566	A/Honduras/5282/2019	EPI_ISL_398533
**A/California/26/2017**	EPI_ISL_252729	A/Singapore/IMH0022/2018	EPI_ISL_350569	A/Nigeria/3778/2019	EPI_ISL_398534
**A/KANAGAWA/AC1612/2016**	EPI_ISL_252766	A/Singapore/EN0707/2018	EPI_ISL_350571	A/Nigeria/3777/2019	EPI_ISL_398535
**A/KANAGAWA/AC1615/2016**	EPI_ISL_252770	A/Singapore/GP1522/2018	EPI_ISL_350572	A/Hawaii/74/2019	EPI_ISL_398536
**A/KANAGAWA/IC1605/2016**	EPI_ISL_252772	A/Singapore/GP1594/2018	EPI_ISL_350574	A/Honduras/5344/2019	EPI_ISL_398538
**A/Indiana/13/2017**	EPI_ISL_253185	A/Singapore/KK0924/2018	EPI_ISL_350576	A/Washington/659/2019	EPI_ISL_398542
**A/Indiana/14/2017**	EPI_ISL_253186	A/Singapore/KK0964/2018	EPI_ISL_350577	A/Wrexham/5048/2019	EPI_ISL_398667
**A/Kursk/1373/2016**	EPI_ISL_253189	A/Singapore/KK1025/2018	EPI_ISL_350579	A/Aberdare/6417/2019	EPI_ISL_398668
**A/Nebraska/04/2017**	EPI_ISL_253193	A/Singapore/GP1974/2018	EPI_ISL_350583	A/Kalmar/1/2019	EPI_ISL_398732
**A/Pakistan/1370/2016**	EPI_ISL_253196	A/Singapore/MOH0137/2018	EPI_ISL_350586	A/Stockholm/37/2019	EPI_ISL_398735
**A/Pakistan/1442/2016**	EPI_ISL_253197	A/Singapore/GP1766/2018	EPI_ISL_350594	A/Singapore/KK1937/2019	EPI_ISL_398746
**A/Texas/50/2017**	EPI_ISL_253205	A/Singapore/KK1107/2018	EPI_ISL_350597	A/Singapore/GP1482/2019	EPI_ISL_398747
**A/Michigan/10/2017**	EPI_ISL_253586	A/Shuberskoye/RII-2201S/2019	EPI_ISL_350608	A/Singapore/GP1657/2019	EPI_ISL_398749
**A/Michigan/16/2017**	EPI_ISL_253594	A/Netherlands/10240/2019	EPI_ISL_350668	A/Nigeria/4557/2019	EPI_ISL_398772
**A/Texas/215/2016**	EPI_ISL_253603	A/Saint-Petersburg/RII-3007S/2019	EPI_ISL_350673	A/Nigeria/2235/2019	EPI_ISL_398795
**A/Texas/205/2016**	EPI_ISL_253609	A/Samara/RII-3184S/2019	EPI_ISL_350677	A/South Carolina/15/2019	EPI_ISL_398798
**A/Falun/2/2017**	EPI_ISL_253865	A/Saint-Petersburg/RII-2126S/2019	EPI_ISL_350702	A/Pennsylvania/1024/2019	EPI_ISL_398809
**A/Sweden/16/2017**	EPI_ISL_253884	A/Wrexham/6341/2019	EPI_ISL_350730	A/Nicaragua/1406/2019	EPI_ISL_398810
**A/Sweden/19/2017**	EPI_ISL_253889	A/Nigeria/3572/2018	EPI_ISL_351879	A/Nicaragua/1398/2019	EPI_ISL_398812
**A/Sweden/22/2017**	EPI_ISL_253892	A/California/106/2019	EPI_ISL_351902	A/Nicaragua/1397/2019	EPI_ISL_398813
**A/Stockholm/23/2017**	EPI_ISL_253894	A/Lebanon/252/2019	EPI_ISL_351922	A/Nicaragua/1392/2019	EPI_ISL_398815
**A/New Mexico/07/2017**	EPI_ISL_255339	A/Honduras/3801/2018	EPI_ISL_352005	A/Nicaragua/1383/2019	EPI_ISL_398817
**A/Christchurch/505/2016**	EPI_ISL_255527	A/Louisiana/23/2019	EPI_ISL_352074	A/Nicaragua/1376/2019	EPI_ISL_398818
**A/Singapore/GP0133/2017**	EPI_ISL_255568	A/Nigeria/3631/2018	EPI_ISL_353449	A/New York/49/2019	EPI_ISL_398820
**A/Singapore/GP0138/2017**	EPI_ISL_255569	A/Connecticut/20/2019	EPI_ISL_353450	A/Bangladesh/7190704004/2019	EPI_ISL_398851
**A/Singapore/GP0209/2017**	EPI_ISL_255571	A/New York/24/2019	EPI_ISL_353468	A/Abergele/8675/2019	EPI_ISL_399970
**A/Singapore/KK0071/2017**	EPI_ISL_255580	A/Ohio/11/2019	EPI_ISL_353477	A/Washington/662/2019	EPI_ISL_400005
**A/Singapore/NUH0004/2017**	EPI_ISL_255591	A/Alabama/22/2019	EPI_ISL_353480	A/Abudhabi/067/2019	EPI_ISL_400016
**A/Netherlands/2329/2017**	EPI_ISL_255649	A/West Virginia/24/2019	EPI_ISL_353481	A/Abudhabi/063/2019	EPI_ISL_400019
**A/Singapore/EN0019/2017**	EPI_ISL_255893	A/Pennsylvania/639/2019	EPI_ISL_353485	A/New Jersey/38/2019	EPI_ISL_400025
**A/Kansas/07/2017**	EPI_ISL_255963	A/Florida/27/2019	EPI_ISL_353486	A/Bahrain/682/2019	EPI_ISL_400028
**A/South Carolina/07/2017**	EPI_ISL_256142	A/Florida/26/2019	EPI_ISL_353487	A/Bhutan/1078/2019	EPI_ISL_400031
**A/Illinois/19/2017**	EPI_ISL_256186	A/Kazakhstan/105/2019	EPI_ISL_353490	A/Bhutan/1095/2019	EPI_ISL_400036
**A/Utah/13/2017**	EPI_ISL_256199	A/Missouri/19/2019	EPI_ISL_353493	A/Singapore/GP1559/2019	EPI_ISL_400291
**A/Oklahoma/09/2017**	EPI_ISL_256201	A/Maine/13/2019	EPI_ISL_353512	A/Singapore/GP1619/2019	EPI_ISL_400292
**A/Louisiana/13/2017**	EPI_ISL_256207	A/Montana/27/2019	EPI_ISL_353649	A/Singapore/GP1793/2019	EPI_ISL_400294
**A/Delaware/10/2017**	EPI_ISL_257833	A/Georgia/12/2019	EPI_ISL_354124	A/Brest/2522/2019	EPI_ISL_400340
**A/Florida/11/2017**	EPI_ISL_257841	A/Georgia/09/2019	EPI_ISL_354125	A/Rennes/2647/2019	EPI_ISL_400354
**A/Illinois/23/2017**	EPI_ISL_257848	A/Vermont/18/2019	EPI_ISL_354129	A/Bretagne/2499/2019	EPI_ISL_400356
**A/Maine/10/2017**	EPI_ISL_257852	A/Nevada/22/2019	EPI_ISL_354140	A/Holywell/6458/2019	EPI_ISL_400537
**A/Maryland/14/2017**	EPI_ISL_257854	A/Ohio/15/2019	EPI_ISL_354144	A/Newport/9420/2019	EPI_ISL_400605
**A/Minnesota/17/2017**	EPI_ISL_257857	A/Connecticut/26/2019	EPI_ISL_354174	A/Peru/3519/2019	EPI_ISL_400775
**A/New Mexico/15/2017**	EPI_ISL_257871	A/Tennessee/61/2019	EPI_ISL_354176	A/Togo/1136/2019	EPI_ISL_400779
**A/Rhode Island/03/2017**	EPI_ISL_257878	A/California/123/2019	EPI_ISL_354184	A/Togo/1198/2019	EPI_ISL_400781
**A/Texas/65/2017**	EPI_ISL_257892	A/Abu Dhabi/022/2019	EPI_ISL_354195	A/Peru/0519/2019	EPI_ISL_400783
**A/Utah/17/2017**	EPI_ISL_257893	A/Sweden/45/2019	EPI_ISL_355225	A/Togo/1257/2019	EPI_ISL_400791

**A/Washington/29/2017**	EPI_ISL_257901	A/Sweden/46/2019	EPI_ISL_355226	A/Bangladesh/1123/2019	EPI_ISL_400802
**A/Netherlands/2947/2017**	EPI_ISL_258248	A/Sweden/52/2019	EPI_ISL_355231	A/Jamaica/60946/2019	EPI_ISL_400811
**A/West Virginia/03/2017**	EPI_ISL_258302	A/Oman/2593/2019	EPI_ISL_355533	A/El Salvador/723/2019	EPI_ISL_400825
**A/Massachusetts/08/2017**	EPI_ISL_258304	A/Oman/2482/2019	EPI_ISL_355534	A/Jamaica/60918/2019	EPI_ISL_400831
**A/New York/13/2017**	EPI_ISL_258319	A/Nigeria/3623/2018	EPI_ISL_355536	A/Jamaica/60904/2019	EPI_ISL_400841
**A/New Jersey/16/2017**	EPI_ISL_258324	A/Nigeria/3652/2018	EPI_ISL_355539	A/Jamaica/5258/2019	EPI_ISL_400846
**A/Puerto Rico/04/2017**	EPI_ISL_258339	A/Nigeria/3670/2018	EPI_ISL_355540	A/Jamaica/10655/2019	EPI_ISL_400852
**A/Indiana/18/2017**	EPI_ISL_258371	A/Abu Dhabi/019/2019	EPI_ISL_355578	A/Jamaica/10164/2019	EPI_ISL_400853
**A/Texas/70/2017**	EPI_ISL_258393	A/Indiana/26/2019	EPI_ISL_355582	A/North Carolina/31/2019	EPI_ISL_400875
**A/Sweden/27/2017**	EPI_ISL_258806	A/Abu Dhabi/029/2019	EPI_ISL_355587	A/Florida/130/2019	EPI_ISL_400876
**A/Sweden/36/2017**	EPI_ISL_258819	A/Burkina Faso/2454/2019	EPI_ISL_355602	A/Pennsylvania/1028/2019	EPI_ISL_400879
**A/Sweden/40/2017**	EPI_ISL_258826	A/Louisiana/32/2019	EPI_ISL_355611	A/Maryland/41/2019	EPI_ISL_400882
**A/Sweden/50/2017**	EPI_ISL_258911	A/Illinois/27/2019	EPI_ISL_355623	A/Colorado/46/2019	EPI_ISL_400883
**A/Sweden/67/2017**	EPI_ISL_258927	A/Oman/2260/2019	EPI_ISL_355642	A/Illinois/37/2019	EPI_ISL_400981
**A/Michigan/31/2017**	EPI_ISL_259085	A/Oman/2163/2019	EPI_ISL_355647	A/Minnesota/59/2019	EPI_ISL_400994
**A/Michigan/28/2017**	EPI_ISL_259101	A/Oman/1405/2019	EPI_ISL_355653	A/Peru/6019/2019	EPI_ISL_401010
**A/Macedonia/25/2016**	EPI_ISL_259175	A/Pakistan/1213/2019	EPI_ISL_355699	A/California/207/2019	EPI_ISL_401011
**A/Macedonia/34/2017**	EPI_ISL_259177	A/Pakistan/1065/2019	EPI_ISL_355700	A/Peru/6619/2019	EPI_ISL_401013
**A/New Mexico/17/2017**	EPI_ISL_259262	A/Pakistan/984/2019	EPI_ISL_355701	A/Bangladesh/1729/2019	EPI_ISL_401016
**A/Ulaanbaatar/574/2017**	EPI_ISL_259696	A/Pakistan/959/2019	EPI_ISL_355702	A/Bangladesh/2050/2019	EPI_ISL_401317
**A/Khovd/240/2017**	EPI_ISL_259702	A/Vina_del_Mar/52625/2018	EPI_ISL_355796	A/Bangladesh/1025/2019	EPI_ISL_401321
**A/New York/17/2017**	EPI_ISL_259704	A/Iquique/64315/2018	EPI_ISL_355800	A/Singapore/GP2299/2019	EPI_ISL_401464
**A/Maine/17/2017**	EPI_ISL_259707	A/Iquique/64319/2018	EPI_ISL_355802	A/Singapore/KK2585/2019	EPI_ISL_401466
**A/Maryland/25/2017**	EPI_ISL_259708	A/Iquique/67248/2018	EPI_ISL_355812	A/Baden-Wuerttemberg/310/2019	EPI_ISL_401784
**A/Maryland/28/2017**	EPI_ISL_259710	A/Iquique/67395/2018	EPI_ISL_355818	A/Mecklenburg-Vorpommern/11/2019	EPI_ISL_401787
**A/Vermont/09/2017**	EPI_ISL_259711	A/Temuco/79796/2018	EPI_ISL_355827	A/Argentina/2852/2019	EPI_ISL_402399
**A/Connecticut/15/2017**	EPI_ISL_259719	A/Santiago/56993/2018	EPI_ISL_355831	A/Argentina/2840/2019	EPI_ISL_402400
**A/Netherlands/096/2017**	EPI_ISL_259986	A/Santiago/57803/2018	EPI_ISL_355832	A/Iowa/63/2019	EPI_ISL_402403
**A/Alabama/10/2017**	EPI_ISL_260385	A/Santiago/59280/2018	EPI_ISL_355834	A/Illinois/38/2019	EPI_ISL_402408
**A/Alabama/16/2017**	EPI_ISL_260392	A/Santiago/82875/2018	EPI_ISL_355839	A/Philippines/1500/2019	EPI_ISL_402428
**A/Arizona/13/2017**	EPI_ISL_260397	A/Temuco/84454/2018	EPI_ISL_355840	A/Ituverava/19180886-IAL/2019	EPI_ISL_402434
**A/Indiana/22/2017**	EPI_ISL_260409	A/Concepcion/47503/2018	EPI_ISL_355842	A/Peru/555919/2019	EPI_ISL_402460
**A/Louisiana/25/2017**	EPI_ISL_260413	A/Iquique/16325/2019	EPI_ISL_355846	A/Utah/62/2019	EPI_ISL_402468
**A/Nevada/09/2017**	EPI_ISL_260416	A/Iquique/19398/2019	EPI_ISL_355847	A/Hawaii/80/2019	EPI_ISL_402479
**A/Utah/24/2017**	EPI_ISL_260424	A/Iquique/21652/2019	EPI_ISL_355849	A/Ecuador/4321/2019	EPI_ISL_402491
**A/Linkoping/1/2017**	EPI_ISL_261644	A/Nigeria/3620/2018	EPI_ISL_356223	A/Michigan/458/2019	EPI_ISL_402516
**A/Michigan/44/2017**	EPI_ISL_261813	A/Nigeria/3622/2018	EPI_ISL_356224	A/Wisconsin/585/2019	EPI_ISL_402520
**A/Ulaanbaatar/34/2017**	EPI_ISL_261865	A/Nigeria/3608/2018	EPI_ISL_356225	A/Chile/2482/2019	EPI_ISL_402525
**A/Connecticut/18/2017**	EPI_ISL_262724	A/New York/35/2019	EPI_ISL_356241	A/Iowa/66/2019	EPI_ISL_402723
**A/Massachusetts/11/2017**	EPI_ISL_262740	A/New York/31/2019	EPI_ISL_356246	A/Iowa/68/2019	EPI_ISL_402724
**A/Puerto Rico/06/2017**	EPI_ISL_262751	A/Maryland/21/2019	EPI_ISL_356248	A/Maryland/47/2019	EPI_ISL_402733
**A/Tennessee/38/2017**	EPI_ISL_262756	A/Kentucky/27/2019	EPI_ISL_356257	A/Virginia/44/2019	EPI_ISL_402735
**A/Vermont/11/2017**	EPI_ISL_262757	A/Vermont/21/2019	EPI_ISL_356258	A/Pennsylvania/1031/2019	EPI_ISL_402740
**A/Vermont/12/2017**	EPI_ISL_262758	A/Vermont/22/2019	EPI_ISL_356259	A/Pennsylvania/1034/2019	EPI_ISL_402741
**A/KANAGAWA/AC1625/2017**	EPI_ISL_262985	A/Montana/29/2019	EPI_ISL_356266	A/Florida/136/2019	EPI_ISL_402748
**A/KANAGAWA/AC1626/2017**	EPI_ISL_262986	A/New Mexico/30/2019	EPI_ISL_356272	A/Massachusetts/43/2019	EPI_ISL_402756
**A/KANAGAWA/IC1623/2017**	EPI_ISL_262988	A/New Mexico/31/2019	EPI_ISL_356273	A/Niger/7204/2019	EPI_ISL_402776
**A/KANAGAWA/IC1624/2017**	EPI_ISL_262989	A/Utah/32/2019	EPI_ISL_356274	A/Palmas/19019981-IAL/2019	EPI_ISL_402778
**A/KANAGAWA/IC1625/2017**	EPI_ISL_262990	A/Connecticut/27/2019	EPI_ISL_356284	A/Sao Paulo/1334848-IAL/2019	EPI_ISL_402782
**A/Stockholm/26/2017**	EPI_ISL_263021	A/Mississippi/26/2019	EPI_ISL_356290	A/Sao Paulo/1318447-IAL/2019	EPI_ISL_402784
**A/Linkoping/4/2017**	EPI_ISL_263022	A/Burkina Faso/1077/2018	EPI_ISL_356300	A/Japan/9879/2019	EPI_ISL_403088
**A/Lund/2/2017**	EPI_ISL_263028	A/Burkina Faso/1057/2018	EPI_ISL_356301	A/Maryland/9883/2019	EPI_ISL_403092
**A/Kalmar/1/2017**	EPI_ISL_263032	A/Burkina Faso/1055/2018	EPI_ISL_356302	A/Nevada/9884/2019	EPI_ISL_403093
**A/Florida/30/2017**	EPI_ISL_264423	A/Cwmbran/1121/2019	EPI_ISL_356568	A/New York/9889/2019	EPI_ISL_403097
**A/Maryland/35/2017**	EPI_ISL_264449	A/Cardiff/0508/2019	EPI_ISL_356569	A/Texas/9897/2019	EPI_ISL_403104
**A/Mississippi/16/2017**	EPI_ISL_264459	A/Llantwit Major/0582/2019	EPI_ISL_356571	A/Virginia/9898/2019	EPI_ISL_403105
**A/New Hampshire/20/2017**	EPI_ISL_264460	A/Anglesey/0643/2019	EPI_ISL_356574	A/Virginia/9899/2019	EPI_ISL_403106
**A/New York/27/2017**	EPI_ISL_264465	A/KANAGAWA/AC1878/2019	EPI_ISL_356752	A/Virginia/9901/2019	EPI_ISL_403108
**A/Ohio/11/2017**	EPI_ISL_264467	A/Linkoping/11/2019	EPI_ISL_356841	A/Washington/9903/2019	EPI_ISL_403110
**A/Santiago/23577/2017**	EPI_ISL_264477	A/Sundsvall/4/2019	EPI_ISL_356848	A/England/9865/2019	EPI_ISL_403215
**A/South Carolina/13/2017**	EPI_ISL_264481	A/Alingsas/1/2019	EPI_ISL_356849	A/Sweden/139/2019	EPI_ISL_403225
**A/Texas/106/2017**	EPI_ISL_264484	A/Gavle/8/2019	EPI_ISL_356858	A/Vina_Del_Mar/82289/2019	EPI_ISL_403671
**A/Washington/37/2017**	EPI_ISL_264490	A/Stockholm/16/2019	EPI_ISL_356866	A/Iquique/82820/2019	EPI_ISL_403672
**A/Wisconsin/48/2017**	EPI_ISL_264492	A/Belgium/G0317/2019	EPI_ISL_357125	A/Iquique/89229/2019	EPI_ISL_403675
**A/Wisconsin/313/2017**	EPI_ISL_266288	A/Belgium/G0318/2019	EPI_ISL_357126	A/Concepcion/90512/2019	EPI_ISL_403676
**A/West Virginia/18/2017**	EPI_ISL_266297	A/Belgium/G0321/2019	EPI_ISL_357128	A/Texas/10024/2019	EPI_ISL_403818
**A/Maryland/36/2017**	EPI_ISL_266298	A/Belgium/G0335/2019	EPI_ISL_357135	A/Gurzuf/RII-997S/2020	EPI_ISL_404169
**A/Michigan/54/2017**	EPI_ISL_266674	A/Belgium/G0338/2019	EPI_ISL_357136	A/Saint-Petersburg/RII-313S/2019	EPI_ISL_404170
**A/Michigan/56/2017**	EPI_ISL_266676	A/Belgium/G0363/2019	EPI_ISL_357146	A/Sweden/149/2019	EPI_ISL_404213
**A/Texas/93/2017**	EPI_ISL_266703	A/Belgium/S0151/2019	EPI_ISL_357157	A/Alaska/10141/2019	EPI_ISL_404290
**A/Utah/26/2017**	EPI_ISL_268231	A/Belgium/S0164/2019	EPI_ISL_357158	A/England/10144/2019	EPI_ISL_404293
**A/Washington/291/2017**	EPI_ISL_268247	A/Belgium/S0194/2019	EPI_ISL_357160	A/Florida/10145/2019	EPI_ISL_404294
**A/South Dakota/21/2017**	EPI_ISL_268256	A/Belgium/S0293/2019	EPI_ISL_357164	A/Missouri/10163/2019	EPI_ISL_404311
**A/South Dakota/23/2017**	EPI_ISL_268258	A/Belgium/S0460/2019	EPI_ISL_357171	A/New York/10169/2019	EPI_ISL_404317
**A/South Dakota/24/2017**	EPI_ISL_268259	A/Belgium/S1346/2019	EPI_ISL_357178	A/South Carolina/10179/2019	EPI_ISL_404327
**A/Florida/39/2017**	EPI_ISL_268260	A/Belgium/S1367/2019	EPI_ISL_357182	A/Chile/5905/2019	EPI_ISL_404534
**A/Florida/40/2017**	EPI_ISL_268261	A/Belgium/S1372/2019	EPI_ISL_357184	A/Chile/2585/2019	EPI_ISL_404536
**A/North Dakota/18/2017**	EPI_ISL_268268	A/Bolivia/4103/2018	EPI_ISL_357808	A/Connecticut/41/2019	EPI_ISL_404537
**A/Valdivia/29696/2017**	EPI_ISL_268291	A/Bangladesh/03253/2018	EPI_ISL_357821	A/Delaware/57/2019	EPI_ISL_404544
**A/Santiago/30424/2017**	EPI_ISL_268293	A/South Dakota/29/2019	EPI_ISL_357846	A/New Hampshire/40/2019	EPI_ISL_404548
**A/Punta Arenas/32893/2017**	EPI_ISL_268297	A/Abu Dhabi/002/2019	EPI_ISL_357866	A/New Jersey/43/2019	EPI_ISL_404551
**A/Antofagasta/33346/2017**	EPI_ISL_268299	A/Abu Dhabi/003/2019	EPI_ISL_357867	A/New York/57/2019	EPI_ISL_404558
**A/Valparaiso/33377/2017**	EPI_ISL_268300	A/Abu Dhabi/006/2019	EPI_ISL_357869	A/Abu Dhabi/085/2019	EPI_ISL_404574
**A/Laos/0753/2017**	EPI_ISL_268301	A/Abu Dhabi/011/2019	EPI_ISL_357872	A/Abu Dhabi/091/2019	EPI_ISL_404578
**A/Tanzania/4345/2017**	EPI_ISL_268318	A/Indiana/32/2019	EPI_ISL_357878	A/Abudhabi/106/2019	EPI_ISL_404580
**A/Tanzania/4384/2017**	EPI_ISL_268330	A/Michigan/287/2019	EPI_ISL_357882	A/Abu Dhabi/104/2019	EPI_ISL_404581
**A/Kyrgyzstan/632/2017**	EPI_ISL_268340	A/Oman/995/2019	EPI_ISL_357912	A/Abu Dhabi/100/2019	EPI_ISL_404584
**A/Parana/99/2017**	EPI_ISL_268344	A/Oman/896/2019	EPI_ISL_357914	A/Abu Dhabi/099/2019	EPI_ISL_404585

**A/Parana/157/2017**	EPI_ISL_268349	A/Oman/939/2019	EPI_ISL_357915	A/Abu Dhabi/098/2019	EPI_ISL_404586
**A/Dnipro/245/2017**	EPI_ISL_268373	A/Oman/951/2019	EPI_ISL_357917	A/Abu Dhabi/094/2019	EPI_ISL_404589
**A/Bangladesh/3028/2017**	EPI_ISL_268375	A/Michigan/84/2018	EPI_ISL_358231	A/Abu Dhabi/092/2019	EPI_ISL_404592
**A/Colombia/3704/2017**	EPI_ISL_268379	A/Texas/104/2018	EPI_ISL_358245	A/Abu Dhabi/079/2019	EPI_ISL_404593
**A/Colombia/3875/2017**	EPI_ISL_268383	A/Texas/106/2018	EPI_ISL_358246	A/Colorado/51/2019	EPI_ISL_404599
**A/Colombia/960/2017**	EPI_ISL_268385	A/Texas/120/2018	EPI_ISL_358268	A/Alaska/59/2019	EPI_ISL_404600
**A/Colombia/961/2017**	EPI_ISL_268386	A/Texas/122/2018	EPI_ISL_358269	A/Hawaii/83/2019	EPI_ISL_404602
**A/South Carolina/15/2017**	EPI_ISL_268387	A/Texas/128/2018	EPI_ISL_358271	A/Utah/65/2019	EPI_ISL_404618
**A/Washington/40/2017**	EPI_ISL_268410	A/Michigan/312/2017	EPI_ISL_358327	A/California/232/2019	EPI_ISL_404622
**A/Singapore/GP0269/2017**	EPI_ISL_269698	A/Michigan/36/2018	EPI_ISL_358395	A/Chile/1978/2019	EPI_ISL_404630
**A/Singapore/GP0423/2017**	EPI_ISL_269699	A/Michigan/41/2018	EPI_ISL_358404	A/Chile/1699/2019	EPI_ISL_404631
**A/Singapore/KK0262/2017**	EPI_ISL_269702	A/Michigan/70/2018	EPI_ISL_358480	A/Chile/7868/2019	EPI_ISL_404633
**A/Singapore/TT0348/2017**	EPI_ISL_269763	A/Michigan/11/2019	EPI_ISL_358629	A/Canberra/185/2019	EPI_ISL_404954
**A/Arizona/35/2017**	EPI_ISL_270174	A/Pennsylvania/30/2019	EPI_ISL_358710	A/Canberra/187/2019	EPI_ISL_404955
**A/Arizona/38/2017**	EPI_ISL_270176	A/Michigan/04/2019	EPI_ISL_358713	A/Victoria/757/2019	EPI_ISL_404959
**A/Honduras/0112/2017**	EPI_ISL_270187	A/Pennsylvania/533/2018	EPI_ISL_358754	A/Victoria/759/2019	EPI_ISL_404960
**A/Kazakhstan/6053/2016**	EPI_ISL_270191	A/Washington/14/2019	EPI_ISL_358786	A/Victoria/2177/2019	EPI_ISL_404963
**A/Kazakhstan/6289/2016**	EPI_ISL_270196	A/Texas/528/2018	EPI_ISL_358839	A/Guam/10291/2019	EPI_ISL_405417
**A/Arizona/42/2017**	EPI_ISL_272880	A/Wisconsin/42/2019	EPI_ISL_358971	A/Mississippi/10309/2019	EPI_ISL_405435
**A/Arizona/43/2017**	EPI_ISL_272881	A/Texas/97/2019	EPI_ISL_359032	A/South Korea/10327/2019	EPI_ISL_405453
**A/Bolivia/795/2017**	EPI_ISL_272882	A/Tennessee/18/2019	EPI_ISL_359246	A/South Korea/10333/2019	EPI_ISL_405459
**A/Ecuador/1120/2017**	EPI_ISL_272886	A/Tennessee/39/2019	EPI_ISL_359250	A/South Korea/10336/2019	EPI_ISL_405462
**A/Ecuador/3602/2017**	EPI_ISL_272890	A/Tennessee/27/2019	EPI_ISL_359253	A/Virginia/10352/2019	EPI_ISL_405478
**A/Ecuador/623/2017**	EPI_ISL_272891	A/Tennessee/44/2019	EPI_ISL_359283	A/Laos/2453/2019	EPI_ISL_405728
**A/Ecuador/6771/2017**	EPI_ISL_272895	A/Tennessee/49/2019	EPI_ISL_359286	A/Maine/01/2020	EPI_ISL_405731
**A/Ecuador/822/2017**	EPI_ISL_272896	A/Pennsylvania/725/2019	EPI_ISL_359396	A/Laos/2461/2019	EPI_ISL_405743
**A/Florida/44/2017**	EPI_ISL_272900	A/Pennsylvania/700/2019	EPI_ISL_359403	A/Vologda/RII-1090S/2020	EPI_ISL_407100
**A/Florida/45/2017**	EPI_ISL_272901	A/Pennsylvania/747/2019	EPI_ISL_359410	A/Berlin/1/2020	EPI_ISL_407195
**A/Hawaii/32/2017**	EPI_ISL_272905	A/Pennsylvania/758/2019	EPI_ISL_359418	A/Thueringen/2/2020	EPI_ISL_407196
**A/Honduras/0234/2017**	EPI_ISL_272907	A/Pennsylvania/764/2019	EPI_ISL_359419	A/Nordrhein-Westfalen/5/2020	EPI_ISL_407198
**A/Uruguay/14/2017**	EPI_ISL_272933	A/Pennsylvania/781/2019	EPI_ISL_359430	A/Berlin/3/2020	EPI_ISL_407200
**A/West Virginia/21/2017**	EPI_ISL_272943	A/Michigan/132/2019	EPI_ISL_359447	A/Berlin/4/2020	EPI_ISL_407201
**A/Analavory/2831/2017**	EPI_ISL_273671	A/Michigan/153/2019	EPI_ISL_359486	A/Bayern/2/2020	EPI_ISL_407205
**A/Peru/3217/2017**	EPI_ISL_273673	A/Michigan/148/2019	EPI_ISL_359488	A/Bretagne/2831/2019	EPI_ISL_408449
**A/Peru/9517/2017**	EPI_ISL_273675	A/Michigan/177/2019	EPI_ISL_359511	A/Lorraine/064/2020	EPI_ISL_408458
**A/Peru/5817/2017**	EPI_ISL_273677	A/Wisconsin/99/2019	EPI_ISL_359553	A/Dijon/084/2020	EPI_ISL_408461
**A/Peru/6817/2017**	EPI_ISL_273685	A/Wisconsin/101/2019	EPI_ISL_359554	A/Delaware/01/2020	EPI_ISL_409029
**A/Peru/2217/2017**	EPI_ISL_273687	A/Pennsylvania/838/2019	EPI_ISL_359618	A/Delaware/60/2019	EPI_ISL_409030
**A/Peru/6917/2017**	EPI_ISL_273692	A/Michigan/249/2019	EPI_ISL_359633	A/Hong Kong/4672/2019	EPI_ISL_409033
**A/Peru/0817/2017**	EPI_ISL_273694	A/Wisconsin/222/2019	EPI_ISL_359872	A/New Hampshire/44/2019	EPI_ISL_409034
**A/Peru/1617/2017**	EPI_ISL_273695	A/Texas/168/2019	EPI_ISL_359963	A/Illinois/40/2019	EPI_ISL_409041
**A/Peru/9417/2017**	EPI_ISL_273696	A/Washington/262/2019	EPI_ISL_359982	A/Illinois/39/2019	EPI_ISL_409043
**A/Magadan/163/2017**	EPI_ISL_273707	A/Washington/287/2019	EPI_ISL_360018	A/Nakhon Ratchasima/1382/2019	EPI_ISL_409052
**A/Texas/277/2017**	EPI_ISL_273714	A/Washington/354/2019	EPI_ISL_360062	A/Nakhonsithammarat/4434/2019	EPI_ISL_409053
**A/Connecticut/25/2017**	EPI_ISL_273720	A/Washington/443/2019	EPI_ISL_360069	A/Colombia/7231/2019	EPI_ISL_409065
**A/Puerto Rico/11/2017**	EPI_ISL_274443	A/Washington/453/2019	EPI_ISL_360075	A/Colombia/7394/2019	EPI_ISL_409068
**A/Puerto Rico/13/2017**	EPI_ISL_274445	A/Washington/367/2019	EPI_ISL_360092	A/Minnesota/73/2019	EPI_ISL_409074
**A/Puerto Rico/20/2017**	EPI_ISL_274451	A/Washington/468/2019	EPI_ISL_360107	A/Mississippi/40/2019	EPI_ISL_409082
**A/Brazil/1770/2017**	EPI_ISL_275737	A/Washington/471/2019	EPI_ISL_360114	A/Hong Kong/4658/2019	EPI_ISL_409084
**A/Brazil/9849/2017**	EPI_ISL_275739	A/Washington/387/2019	EPI_ISL_360129	A/Wisconsin/03/2020	EPI_ISL_409088
**A/Brazil/9114/2017**	EPI_ISL_275741	A/Washington/422/2019	EPI_ISL_360135	A/Wisconsin/01/2020	EPI_ISL_409089
**A/Argentina/164/2017**	EPI_ISL_275747	A/Washington/414/2019	EPI_ISL_360148	A/Wisconsin/04/2020	EPI_ISL_409090
**A/Argentina/97/2017**	EPI_ISL_275750	A/Wisconsin/08/2018	EPI_ISL_360274	A/Nevada/49/2019	EPI_ISL_409101
**A/Bangladesh/0016/2017**	EPI_ISL_275774	A/Wisconsin/23/2018	EPI_ISL_360289	A/California/244/2019	EPI_ISL_409103
**A/Para/145486-IEC/2017**	EPI_ISL_275807	A/Wisconsin/34/2018	EPI_ISL_360300	A/Vermont/33/2019	EPI_ISL_409118
**A/Hong Kong/3555/2017**	EPI_ISL_275814	A/Pennsylvania/09/2018	EPI_ISL_360309	A/New York/07/2020	EPI_ISL_409127
**A/Ceara/145874-IEC/2017**	EPI_ISL_275817	A/Pennsylvania/10/2018	EPI_ISL_360311	A/El Salvador/859/2019	EPI_ISL_409341
**A/Pernambuco/146140-IEC/2017**	EPI_ISL_275819	A/Pennsylvania/12/2018	EPI_ISL_360315	A/Laos/2549/2019	EPI_ISL_409364
**A/Hong Kong/3549/2017**	EPI_ISL_275824	A/Pennsylvania/301/2017	EPI_ISL_360332	A/Bulgaria/072/2020	EPI_ISL_409368
**A/Roraima/146724-IEC/2017**	EPI_ISL_275827	A/Pennsylvania/30/2018	EPI_ISL_360343	A/Hong Kong/4657/2019	EPI_ISL_409378
**A/Paraiba/146792-IEC/2017**	EPI_ISL_275829	A/Pennsylvania/311/2017	EPI_ISL_360344	A/Paraguay/9880/2019	EPI_ISL_409379
**A/Acre/146845-IEC/2017**	EPI_ISL_275831	A/Pennsylvania/319/2017	EPI_ISL_360358	A/Washington/695/2019	EPI_ISL_409381
**A/Roraima/148122-IEC/2017**	EPI_ISL_275839	A/Texas/32/2018	EPI_ISL_360378	A/Washington/696/2019	EPI_ISL_409383
**A/Hong Kong/3569/2017**	EPI_ISL_275840	A/Texas/354/2017	EPI_ISL_360382	A/Texas/17/2020	EPI_ISL_409384
**A/Panama/322630/2017**	EPI_ISL_275851	A/Texas/390/2017	EPI_ISL_360385	A/Texas/438/2019	EPI_ISL_409385
**A/Brazil/291/2017**	EPI_ISL_275862	A/Texas/11/2018	EPI_ISL_360386	A/Hong Kong/4669/2019	EPI_ISL_409388
**A/Brazil/321/2017**	EPI_ISL_275865	A/Texas/48/2018	EPI_ISL_360392	A/Hong Kong/4641/2019	EPI_ISL_409392
**A/Brazil/339/2017**	EPI_ISL_275866	A/Texas/384/2017	EPI_ISL_360395	A/Hong Kong/4711/2019	EPI_ISL_409402
**A/Brazil/399/2017**	EPI_ISL_275869	A/Texas/380/2017	EPI_ISL_360418	A/Hong Kong/4710/2019	EPI_ISL_409403
**A/Brazil/474/2017**	EPI_ISL_275873	A/Texas/45/2018	EPI_ISL_360423	A/Hong Kong/4709/2019	EPI_ISL_409404
**A/Brazil/475/2017**	EPI_ISL_275875	A/Texas/21/2018	EPI_ISL_360458	A/Burkina Faso/1294/2019	EPI_ISL_409415
**A/Brazil/479/2017**	EPI_ISL_275879	A/Texas/53/2018	EPI_ISL_360462	A/Peru/1919/2019	EPI_ISL_409418
**A/Uruguay/343/2017**	EPI_ISL_275880	A/Texas/387/2017	EPI_ISL_360464	A/Peru/6219/2019	EPI_ISL_409422
**A/Brazil/482/2017**	EPI_ISL_275881	A/Michigan/96/2018	EPI_ISL_360471	A/Peru/6212/2019	EPI_ISL_409428
**A/Brazil/486/2017**	EPI_ISL_275883	A/Michigan/225/2018	EPI_ISL_360495	A/California/03/2020	EPI_ISL_409678
**A/Brazil/491/2017**	EPI_ISL_275885	A/Michigan/363/2018	EPI_ISL_360518	A/Arizona/56/2019	EPI_ISL_409680
**A/Brazil/492/2017**	EPI_ISL_275887	A/Michigan/371/2018	EPI_ISL_360519	A/Utah/01/2020	EPI_ISL_409681
**A/Guatemala/199/2017**	EPI_ISL_275888	A/Michigan/118/2018	EPI_ISL_360527	A/Montana/02/2020	EPI_ISL_409682
**A/Guatemala/184/2017**	EPI_ISL_275894	A/Michigan/108/2018	EPI_ISL_360538	A/Berlin/9/2020	EPI_ISL_410081
**A/Santiago/53918/2017**	EPI_ISL_275895	A/Michigan/378/2018	EPI_ISL_360552	A/Bayern/12/2020	EPI_ISL_410965
**A/Guatemala/124/2017**	EPI_ISL_275898	A/Michigan/88/2018	EPI_ISL_360558	A/Baden-Wuerttemberg/20/2020	EPI_ISL_410970
**A/Guatemala/940/2017**	EPI_ISL_275902	A/Michigan/144/2018	EPI_ISL_360563	A/Hessen/6/2020	EPI_ISL_410972
**A/Talca/55005/2017**	EPI_ISL_275909	A/Michigan/152/2018	EPI_ISL_360568	A/Bayern/29/2020	EPI_ISL_410975
**A/Copiapo/56066/2017**	EPI_ISL_275925	A/Michigan/138/2018	EPI_ISL_360579	A/Berlin/19/2020	EPI_ISL_410976
**A/Calama/56093/2017**	EPI_ISL_275929	A/Michigan/147/2018	EPI_ISL_360581	A/Berlin/18/2020	EPI_ISL_410980
**A/Iquique/56115/2017**	EPI_ISL_275931	A/Michigan/164/2018	EPI_ISL_360618	A/NAURU/5/2011	EPI_ISL_95078
**A/Los Angeles/56834/2017**	EPI_ISL_275939	A/Michigan/188/2018	EPI_ISL_360626	A/Belgium/G0062/2017	EPI_ISL_415218
**A/Suriname/1977/2017**	EPI_ISL_275945	A/Michigan/238/2018	EPI_ISL_360629	A/Belgium/S0773/2017	EPI_ISL_415380

**A/Suriname/2032/2017**	EPI_ISL_275949	A/Michigan/191/2018	EPI_ISL_360642	A/Belgium/S0058/2017	EPI_ISL_415295
**A/Wyoming/18/2017**	EPI_ISL_275971	A/Michigan/192/2018	EPI_ISL_360646	A/Belgium/S0789/2017	EPI_ISL_415384
**A/Michigan/275/2017**	EPI_ISL_275974	A/Michigan/203/2018	EPI_ISL_360650	A/Belgium/G0457/2017	EPI_ISL_415280
**A/Ohio/32/2017**	EPI_ISL_275977	A/Michigan/204/2018	EPI_ISL_360656	A/Belgium/S0138/2017	EPI_ISL_415303
**A/Texas/280/2017**	EPI_ISL_275980	A/Michigan/252/2018	EPI_ISL_360664	A/Belgium/S0803/2017	EPI_ISL_415387
**A/Florida/50/2017**	EPI_ISL_275983	A/Michigan/195/2018	EPI_ISL_360670	A/Belgium/G0271/2017	EPI_ISL_415245
**A/Florida/51/2017**	EPI_ISL_275984	A/Michigan/214/2018	EPI_ISL_360680	A/Belgium/S0746/2017	EPI_ISL_415373
**A/California/65/2017**	EPI_ISL_275987	A/Michigan/271/2018	EPI_ISL_360689	A/Belgium/G0311/2017	EPI_ISL_415283
**A/Wisconsin/318/2017**	EPI_ISL_275989	A/Michigan/262/2018	EPI_ISL_360691	A/Belgium/S0892/2017	EPI_ISL_415398
**A/Uppsala/1/2017**	EPI_ISL_276914	A/Michigan/233/2018	EPI_ISL_360694	A/Belgium/S1252/2017	EPI_ISL_415434
**A/Brazil/400/2017**	EPI_ISL_277140	A/Michigan/220/2018	EPI_ISL_360705	A/Belgium/S0544/2017	EPI_ISL_415355
**A/Bangladesh/2210/2017**	EPI_ISL_277157	A/Michigan/289/2018	EPI_ISL_360706	A/Belgium/S0113/2017	EPI_ISL_415301
**A/New York/32/2017**	EPI_ISL_277161	A/Michigan/285/2018	EPI_ISL_360723	A/Belgium/S0499/2017	EPI_ISL_415347
**A/Alaska/27/2017**	EPI_ISL_277164	A/Michigan/298/2018	EPI_ISL_360726	A/Belgium/S1068/2017	EPI_ISL_415416
**A/Alaska/28/2017**	EPI_ISL_277165	A/Michigan/309/2018	EPI_ISL_360731	A/Belgium/S0679/2017	EPI_ISL_415366
**A/Alaska/36/2017**	EPI_ISL_277173	A/Michigan/328/2018	EPI_ISL_360740	A/Belgium/S1029/2017	EPI_ISL_415413
**A/Honduras/379/2017**	EPI_ISL_277190	A/Michigan/332/2018	EPI_ISL_360742	A/Belgium/S1475/2017	EPI_ISL_415452
**A/Honduras/482/2017**	EPI_ISL_277199	A/Michigan/342/2018	EPI_ISL_360756	A/Belgium/G0462/2017	EPI_ISL_415281
**A/Honduras/505/2017**	EPI_ISL_277202	A/Michigan/313/2018	EPI_ISL_360769	A/Belgium/S0872/2017	EPI_ISL_415393
**A/Bangladesh/8007/2017**	EPI_ISL_277271	A/Michigan/314/2018	EPI_ISL_360774	A/Belgium/G0071/2017	EPI_ISL_415220
**A/Florida/55/2017**	EPI_ISL_277277	A/Michigan/315/2018	EPI_ISL_360776	A/Belgium/G0453/2017	EPI_ISL_415278
**A/Colombia/4923/2017**	EPI_ISL_277494	A/Michigan/352/2018	EPI_ISL_360780	A/Belgium/G0205/2017	EPI_ISL_415231
**A/Colombia/5071/2017**	EPI_ISL_277496	A/Michigan/320/2018	EPI_ISL_360781	A/Belgium/S0897/2017	EPI_ISL_415400
**A/Colombia/5118/2017**	EPI_ISL_277498	A/Michigan/322/2018	EPI_ISL_360798	A/Belgium/S0750/2017	EPI_ISL_415375
**A/Colombia/5131/2017**	EPI_ISL_277500	A/Michigan/361/2018	EPI_ISL_360801	A/Belgium/S0455/2017	EPI_ISL_415336
**A/Thailand/46/2017**	EPI_ISL_277537	A/Michigan/329/2018	EPI_ISL_360812	A/Belgium/S0451/2017	EPI_ISL_415334
**A/Arizona/48/2017**	EPI_ISL_277845	A/Michigan/319/2018	EPI_ISL_360816	A/Belgium/G0070/2017	EPI_ISL_415219
**A/Hawaii/41/2017**	EPI_ISL_277850	A/Michigan/367/2018	EPI_ISL_360819	A/Belgium/S0456/2017	EPI_ISL_415337
**A/Hawaii/42/2017**	EPI_ISL_277851	A/Michigan/370/2018	EPI_ISL_360821	A/Belgium/S0443/2017	EPI_ISL_415332
**A/Louisiana/49/2017**	EPI_ISL_277853	A/Tennessee/94/2017	EPI_ISL_360839	A/Belgium/S0078/2017	EPI_ISL_415297
**A/Massachusetts/14/2017**	EPI_ISL_277856	A/Tennessee/95/2017	EPI_ISL_360840	A/Belgium/S0447/2017	EPI_ISL_415333
**A/Mississippi/19/2017**	EPI_ISL_277861	A/Tennessee/38/2018	EPI_ISL_360848	A/Belgium/S0150/2017	EPI_ISL_415305
**A/Ohio/37/2017**	EPI_ISL_277867	A/Tennessee/47/2018	EPI_ISL_360859	A/Belgium/S0693/2017	EPI_ISL_415367
**A/Pennsylvania/259/2017**	EPI_ISL_277869	A/Tennessee/48/2018	EPI_ISL_360861	A/Belgium/S0281/2017	EPI_ISL_415313
**A/Pennsylvania/262/2017**	EPI_ISL_277872	A/Tennessee/49/2018	EPI_ISL_360863	A/Belgium/G0242/2017	EPI_ISL_415238
**A/Stockholm/28/2017**	EPI_ISL_281231	A/Washington/136/2018	EPI_ISL_360864	A/Belgium/G0240/2017	EPI_ISL_415237
**A/Bolivia/0836/2017**	EPI_ISL_281453	A/Washington/135/2018	EPI_ISL_360866	A/Belgium/S0747/2017	EPI_ISL_415374
**A/Bolivia/0892/2017**	EPI_ISL_281458	A/Tennessee/52/2018	EPI_ISL_360871	A/Belgium/S0354/2017	EPI_ISL_415319
**A/Bolivia/0898/2017**	EPI_ISL_281459	A/Washington/141/2018	EPI_ISL_360877	A/Belgium/S0785/2017	EPI_ISL_415383
**A/Bolivia/1006/2017**	EPI_ISL_281462	A/Tennessee/40/2018	EPI_ISL_360878	A/Belgium/S0146/2017	EPI_ISL_415304
**A/Bolivia/1056/2017**	EPI_ISL_281463	A/Tennessee/53/2018	EPI_ISL_360885	A/Belgium/S0489/2017	EPI_ISL_415344
**A/Idaho/26/2017**	EPI_ISL_281490	A/Washington/105/2018	EPI_ISL_360901	A/Belgium/S0459/2017	EPI_ISL_415339
**A/Wisconsin/323/2017**	EPI_ISL_281494	A/Washington/154/2018	EPI_ISL_360904	A/Belgium/S0188/2017	EPI_ISL_415308
**A/Vermont/14/2017**	EPI_ISL_281513	A/Washington/156/2018	EPI_ISL_360905	A/Belgium/S0268/2017	EPI_ISL_415311
**A/Brazil/9098/2017**	EPI_ISL_281542	A/Washington/159/2018	EPI_ISL_360913	A/Belgium/G0682/2016	EPI_ISL_415199
**A/Brazil/8298/2017**	EPI_ISL_281544	A/Washington/113/2018	EPI_ISL_360916	A/Belgium/S0849/2017	EPI_ISL_415390
**A/Paraguay/8873/2017**	EPI_ISL_281550	A/Washington/161/2018	EPI_ISL_360917	A/Belgium/S1383/2017	EPI_ISL_415448
**A/Paraguay/9992/2017**	EPI_ISL_281552	A/Washington/162/2018	EPI_ISL_360919	A/Belgium/S1052/2017	EPI_ISL_415414
**A/Lund/4/2017**	EPI_ISL_281575	A/Washington/116/2018	EPI_ISL_360920	A/Belgium/S0896/2017	EPI_ISL_415399
**A/Stockholm/31/2017**	EPI_ISL_281576	A/Wisconsin/117/2018	EPI_ISL_361185	A/Belgium/S1191/2017	EPI_ISL_415428
**A/Colorado/12/2017**	EPI_ISL_281712	A/Wisconsin/194/2018	EPI_ISL_361260	A/Belgium/G0373/2017	EPI_ISL_415261
**A/Iowa/27/2017**	EPI_ISL_281715	A/Wisconsin/205/2018	EPI_ISL_361270	A/Belgium/G0383/2017	EPI_ISL_415263
**A/Hawaii/44/2017**	EPI_ISL_281716	A/Wisconsin/279/2018	EPI_ISL_361309	A/Belgium/S0938/2017	EPI_ISL_415406
**A/Kazakhstan/084/2017**	EPI_ISL_281724	A/Wisconsin/238/2018	EPI_ISL_361331	A/Belgium/G0099/2017	EPI_ISL_415226
**A/Kazakhstan/082/2017**	EPI_ISL_281725	A/Wisconsin/294/2018	EPI_ISL_361338	A/Belgium/S0422/2017	EPI_ISL_415328
**A/Kazakhstan/126/2017**	EPI_ISL_281730	A/Wisconsin/302/2018	EPI_ISL_361353	A/Belgium/S0516/2017	EPI_ISL_415352
**A/Michigan/287/2017**	EPI_ISL_281736	A/Wisconsin/264/2018	EPI_ISL_361365	A/Belgium/S0413/2017	EPI_ISL_415326
**A/Virginia/31/2017**	EPI_ISL_282826	A/Wisconsin/336/2018	EPI_ISL_361397	A/Belgium/G0297/2017	EPI_ISL_415249
**A/North Carolina/32/2017**	EPI_ISL_282834	A/Wisconsin/340/2018	EPI_ISL_361401	A/Belgium/G0323/2017	EPI_ISL_415254
**A/Wisconsin/328/2017**	EPI_ISL_282837	A/Wisconsin/371/2018	EPI_ISL_361431	A/Belgium/S0457/2017	EPI_ISL_415338
**A/New Hampshire/22/2017**	EPI_ISL_282843	A/Wisconsin/378/2018	EPI_ISL_361438	A/Belgium/S0696/2017	EPI_ISL_415369
**A/Costa Rica/4869/2017**	EPI_ISL_282870	A/Wisconsin/400/2018	EPI_ISL_361456	A/Belgium/G0486/2017	EPI_ISL_415289
**A/South Carolina/16/2017**	EPI_ISL_282876	A/Wisconsin/401/2018	EPI_ISL_361457	A/Belgium/G0128/2017	EPI_ISL_415229
**A/Bangladesh/1013/2017**	EPI_ISL_282880	A/Wisconsin/412/2018	EPI_ISL_361468	A/Belgium/S0494/2017	EPI_ISL_415346
**A/Alaska/62/2017**	EPI_ISL_282898	A/Wisconsin/417/2018	EPI_ISL_361473	A/Belgium/S1413/2017	EPI_ISL_415449
**A/Vaxjo/1/2017**	EPI_ISL_282965	A/Wisconsin/419/2018	EPI_ISL_361475	A/Belgium/G0042/2017	EPI_ISL_415211
**A/Stockholm/36/2017**	EPI_ISL_282969	A/Wisconsin/420/2018	EPI_ISL_361476	A/Belgium/G0354/2017	EPI_ISL_415259
**A/Stockholm/37/2017**	EPI_ISL_282970	A/Wisconsin/425/2018	EPI_ISL_361481	A/Belgium/S1166/2017	EPI_ISL_415424
**A/Uppsala/2/2017**	EPI_ISL_282981	A/Wisconsin/430/2018	EPI_ISL_361486	A/Belgium/G0046/2017	EPI_ISL_415214
**A/Singapore/GP1985/2017**	EPI_ISL_283003	A/Wisconsin/439/2018	EPI_ISL_361492	A/Belgium/G0045/2017	EPI_ISL_415213
**A/Singapore/TT1167/2017**	EPI_ISL_283075	A/Wisconsin/464/2018	EPI_ISL_361557	A/Belgium/S0576/2017	EPI_ISL_415360
**A/Singapore/KK0777/2017**	EPI_ISL_283079	A/Wisconsin/470/2018	EPI_ISL_361572	A/Belgium/S1006/2017	EPI_ISL_415411
**A/Singapore/GP1453/2017**	EPI_ISL_283091	A/Wisconsin/471/2018	EPI_ISL_361575	A/Belgium/G0303/2017	EPI_ISL_415251
**A/Bolivia/785/2017**	EPI_ISL_283165	A/Wisconsin/472/2018	EPI_ISL_361578	A/Belgium/S0917/2017	EPI_ISL_415403
**A/Bolivia/866/2017**	EPI_ISL_283167	A/Wisconsin/475/2018	EPI_ISL_361585	A/Belgium/G0437/2017	EPI_ISL_415272
**A/Bolivia/1729/2017**	EPI_ISL_283171	A/Wisconsin/359/2017	EPI_ISL_361660	A/Belgium/S0484/2017	EPI_ISL_415343
**A/Bolivia/1628/2017**	EPI_ISL_283172	A/Wisconsin/360/2017	EPI_ISL_361665	A/Belgium/G0260/2017	EPI_ISL_415243
**A/Bolivia/1679/2017**	EPI_ISL_283173	A/Wisconsin/361/2017	EPI_ISL_361668	A/Belgium/G0369/2017	EPI_ISL_415260
**A/Wisconsin/331/2017**	EPI_ISL_283202	A/Wisconsin/214/2018	EPI_ISL_361682	A/Belgium/S1104/2017	EPI_ISL_415420
**A/New Jersey/33/2017**	EPI_ISL_284063	A/Wisconsin/320/2018	EPI_ISL_361685	A/Belgium/G0300/2017	EPI_ISL_415250
**A/Florida/73/2017**	EPI_ISL_284064	A/Pennsylvania/168/2018	EPI_ISL_361727	A/Belgium/S0593/2017	EPI_ISL_415363
**A/California/106/2017**	EPI_ISL_284158	A/Pennsylvania/173/2018	EPI_ISL_361732	A/Belgium/S0805/2017	EPI_ISL_415388
**A/Hawaii/52/2017**	EPI_ISL_284167	A/Pennsylvania/177/2018	EPI_ISL_361736	A/Belgium/S1199/2017	EPI_ISL_415430
**A/New York/36/2017**	EPI_ISL_284902	A/Pennsylvania/182/2018	EPI_ISL_361739	A/Belgium/S1337/2017	EPI_ISL_415445
**A/Oklahoma/25/2017**	EPI_ISL_284910	A/Pennsylvania/188/2018	EPI_ISL_361751	A/Belgium/S1118/2017	EPI_ISL_415421
**A/New York/38/2017**	EPI_ISL_285659	A/Pennsylvania/202/2018	EPI_ISL_361765	A/Belgium/S0722/2017	EPI_ISL_415370
**A/California/109/2017**	EPI_ISL_285674	A/Pennsylvania/318/2018	EPI_ISL_361766	A/Belgium/S1329/2017	EPI_ISL_415443
**A/Virginia/33/2017**	EPI_ISL_285749	A/Pennsylvania/326/2018	EPI_ISL_361777	A/Belgium/S0322/2017	EPI_ISL_415317

**A/Halmstad/1/2017**	EPI_ISL_285970	A/Pennsylvania/210/2018	EPI_ISL_361779	A/Belgium/G0037/2017	EPI_ISL_415209
**A/Stockholm/43/2017**	EPI_ISL_285972	A/Pennsylvania/227/2018	EPI_ISL_361797	A/Belgium/G0324/2017	EPI_ISL_415284
**A/Bangladesh/64/2017**	EPI_ISL_286060	A/Pennsylvania/341/2018	EPI_ISL_361801	A/Belgium/G0033/2017	EPI_ISL_415208
**A/Bangladesh/1246/2017**	EPI_ISL_286061	A/Pennsylvania/289/2018	EPI_ISL_361818	A/Belgium/G0209/2017	EPI_ISL_415232
**A/Hong Kong/4828/2017**	EPI_ISL_286062	A/Pennsylvania/351/2018	EPI_ISL_361820	A/Belgium/G0296/2017	EPI_ISL_415248
**A/Bangladesh/3012/2017**	EPI_ISL_286120	A/Pennsylvania/352/2018	EPI_ISL_361822	A/Belgium/G0470/2017	EPI_ISL_415287
**A/Bangladesh/601001/2017**	EPI_ISL_286121	A/Pennsylvania/353/2018	EPI_ISL_361824	A/Belgium/G0433/2017	EPI_ISL_415270
**A/Washington/115/2016**	EPI_ISL_286320	A/Pennsylvania/294/2018	EPI_ISL_361829	A/Belgium/G0090/2017	EPI_ISL_415224
**A/Washington/133/2016**	EPI_ISL_286339	A/Pennsylvania/301/2018	EPI_ISL_361841	A/Belgium/G0040/2017	EPI_ISL_415210
**A/Washington/51/2017**	EPI_ISL_286342	A/Pennsylvania/362/2018	EPI_ISL_361842	A/Belgium/G0232/2017	EPI_ISL_415236
**A/Washington/151/2016**	EPI_ISL_286350	A/Pennsylvania/383/2018	EPI_ISL_361856	A/Belgium/G0003/2017	EPI_ISL_415203
**A/Washington/46/2017**	EPI_ISL_286359	A/Pennsylvania/386/2018	EPI_ISL_361858	A/Belgium/G0096/2017	EPI_ISL_415225
**A/Washington/153/2016**	EPI_ISL_286365	A/Macedonia/1380/2019	EPI_ISL_362163	A/Belgium/G0281/2017	EPI_ISL_415246
**A/Washington/148/2016**	EPI_ISL_286370	A/Macedonia/1402/2019	EPI_ISL_362164	A/Belgium/S0282/2017	EPI_ISL_415314
**A/Washington/71/2017**	EPI_ISL_286410	A/Macedonia/1423/2019	EPI_ISL_362165	A/Belgium/G0513/2017	EPI_ISL_415291
**A/Washington/82/2017**	EPI_ISL_286420	A/Macedonia/1350/2019	EPI_ISL_362166	A/Belgium/G0051/2017	EPI_ISL_415215
**A/Washington/106/2017**	EPI_ISL_286421	A/Macedonia/1438/2019	EPI_ISL_362168	A/Belgium/G0340/2017	EPI_ISL_415257
**A/Washington/118/2017**	EPI_ISL_286437	A/Niger/6513/2019	EPI_ISL_362179	A/Belgium/G0427/2017	EPI_ISL_415267
**A/Washington/113/2017**	EPI_ISL_286439	A/Mali/38/2019	EPI_ISL_362182	A/Belgium/G0023/2017	EPI_ISL_415206
**A/Washington/141/2017**	EPI_ISL_286451	A/Utah/36/2019	EPI_ISL_362195	A/Belgium/S0644/2017	EPI_ISL_415364
**A/Washington/179/2017**	EPI_ISL_286460	A/Colorado/37/2019	EPI_ISL_362197	A/Belgium/S0303/2017	EPI_ISL_415316
**A/Washington/132/2017**	EPI_ISL_286466	A/California/135/2019	EPI_ISL_362206	A/Belgium/G0447/2017	EPI_ISL_415277
**A/Washington/186/2017**	EPI_ISL_286474	A/Oregon/24/2019	EPI_ISL_362207	A/Belgium/G0412/2017	EPI_ISL_415266
**A/Washington/137/2017**	EPI_ISL_286484	A/Arizona/26/2019	EPI_ISL_362208	A/Belgium/G0307/2017	EPI_ISL_415252
**A/Washington/122/2017**	EPI_ISL_286498	A/Montana/35/2019	EPI_ISL_362211	A/Belgium/S1314/2017	EPI_ISL_415441
**A/Washington/207/2017**	EPI_ISL_286499	A/Kenya/104/2019	EPI_ISL_362222	A/Belgium/S1306/2017	EPI_ISL_415440
**A/Washington/209/2017**	EPI_ISL_286503	A/Kenya/118/2019	EPI_ISL_362224	A/Belgium/S0453/2017	EPI_ISL_415335
**A/Washington/192/2017**	EPI_ISL_286529	A/Kenya/120/2019	EPI_ISL_362230	A/Belgium/G0434/2017	EPI_ISL_415271
**A/Wisconsin/72/2017**	EPI_ISL_286536	A/Kenya/121/2019	EPI_ISL_362231	A/Belgium/S0551/2017	EPI_ISL_415357
**A/Washington/205/2017**	EPI_ISL_286557	A/Bolivia/237/2019	EPI_ISL_362234	A/Belgium/S0553/2017	EPI_ISL_415359
**A/Wisconsin/104/2017**	EPI_ISL_286588	A/Bolivia/38/2019	EPI_ISL_362242	A/Belgium/S0549/2017	EPI_ISL_415356
**A/Washington/229/2017**	EPI_ISL_286624	A/Tanzania/3117/2019	EPI_ISL_362245	A/Belgium/G0326/2017	EPI_ISL_415255
**A/Washington/249/2017**	EPI_ISL_286638	A/Tanzania/823/2019	EPI_ISL_362246	A/Belgium/S0675/2017	EPI_ISL_415365
**A/Washington/248/2017**	EPI_ISL_286645	A/Tanzania/3112/2019	EPI_ISL_362252	A/Belgium/S0978/2017	EPI_ISL_415410
**A/Washington/263/2017**	EPI_ISL_286652	A/Tanzania/4024/2019	EPI_ISL_362254	A/Belgium/S1264/2017	EPI_ISL_415436
**A/Washington/258/2017**	EPI_ISL_286654	A/Congo/12/2019	EPI_ISL_362395	A/Belgium/S0385/2017	EPI_ISL_415323
**A/Washington/267/2017**	EPI_ISL_286657	A/Congo/27/2019	EPI_ISL_362396	A/Belgium/G0027/2017	EPI_ISL_415207
**A/Washington/282/2017**	EPI_ISL_286669	A/Florida/41/2019	EPI_ISL_362555	A/Belgium/G0259/2017	EPI_ISL_415242
**A/Washington/283/2017**	EPI_ISL_286673	A/West Virginia/34/2019	EPI_ISL_362597	A/Belgium/S0960/2017	EPI_ISL_415409
**A/Wisconsin/54/2017**	EPI_ISL_286695	A/Massachusetts/28/2019	EPI_ISL_362603	A/Belgium/S1246/2017	EPI_ISL_415432
**A/Wisconsin/56/2017**	EPI_ISL_286699	A/Virginia/33/2019	EPI_ISL_362605	A/Belgium/G0054/2017	EPI_ISL_415216
**A/Wisconsin/173/2017**	EPI_ISL_286726	A/Ulaanbaatar/1101/2019	EPI_ISL_362608	A/Belgium/G0409/2017	EPI_ISL_415265
**A/Wisconsin/208/2017**	EPI_ISL_286761	A/Florida/56/2019	EPI_ISL_362609	A/Belgium/G0020/2017	EPI_ISL_415205
**A/Michigan/199/2017**	EPI_ISL_287030	A/Florida/58/2019	EPI_ISL_362630	A/Belgium/S1268/2017	EPI_ISL_415438
**A/Michigan/229/2017**	EPI_ISL_287089	A/Florida/59/2019	EPI_ISL_362631	A/Belgium/S0910/2017	EPI_ISL_415401
**A/Michigan/235/2017**	EPI_ISL_287104	A/Hawaii/38/2019	EPI_ISL_362639	A/Belgium/S0758/2017	EPI_ISL_415377
**A/Michigan/247/2017**	EPI_ISL_287117	A/Neath/3346/2019	EPI_ISL_362658	A/Belgium/S0028/2017	EPI_ISL_415293
**A/Michigan/256/2017**	EPI_ISL_287126	A/Bangladesh/80914003/2018	EPI_ISL_363639	A/Belgium/S0365/2017	EPI_ISL_415320
**A/Texas/268/2017**	EPI_ISL_287131	A/Connecticut/31/2019	EPI_ISL_363827	A/Belgium/S1266/2017	EPI_ISL_415437
**A/Texas/199/2017**	EPI_ISL_287136	A/Connecticut/32/2019	EPI_ISL_363828	A/Belgium/S0772/2017	EPI_ISL_415379
**A/Texas/237/2017**	EPI_ISL_287143	A/Colombia/4252/2019	EPI_ISL_363829	A/Belgium/S0093/2017	EPI_ISL_415298
**A/Texas/252/2017**	EPI_ISL_287144	A/Colombia/4167/2019	EPI_ISL_363830	A/Belgium/S0098/2017	EPI_ISL_415299
**A/Texas/109/2017**	EPI_ISL_287149	A/Colombia/2312/2019	EPI_ISL_363834	A/Belgium/G0379/2017	EPI_ISL_415262
**A/Texas/182/2017**	EPI_ISL_287152	A/Colombia/2313/2019	EPI_ISL_363838	A/Belgium/S0761/2017	EPI_ISL_415378
**A/Wisconsin/215/2017**	EPI_ISL_287200	A/Michigan/382/2019	EPI_ISL_363857	A/Belgium/G0186/2017	EPI_ISL_415230
**A/Wisconsin/226/2017**	EPI_ISL_287206	A/Missouri/25/2019	EPI_ISL_363859	A/Belgium/G0441/2017	EPI_ISL_415274
**A/Wisconsin/235/2017**	EPI_ISL_287215	A/Maryland/27/2019	EPI_ISL_363860	A/Belgium/G0088/2017	EPI_ISL_415223
**A/Wisconsin/237/2017**	EPI_ISL_287217	A/Mali/28/2019	EPI_ISL_363881	A/Belgium/S0194/2017	EPI_ISL_415309
**A/Wisconsin/249/2017**	EPI_ISL_287235	A/Niger/6617/2019	EPI_ISL_363893	A/Belgium/G0226/2017	EPI_ISL_415235
**A/Wisconsin/271/2017**	EPI_ISL_287255	A/Togo/431/2019	EPI_ISL_363894	A/Belgium/S0287/2017	EPI_ISL_415315
**A/Wisconsin/273/2017**	EPI_ISL_287257	A/Togo/353/2019	EPI_ISL_363897	A/Belgium/S0587/2017	EPI_ISL_415362
**A/Wisconsin/282/2017**	EPI_ISL_287262	A/Togo/319/2019	EPI_ISL_363900	A/Belgium/G0044/2017	EPI_ISL_415212
**A/Wisconsin/295/2017**	EPI_ISL_287274	A/Togo/299/2019	EPI_ISL_363901	A/Belgium/S0508/2017	EPI_ISL_415350
**A/Wisconsin/297/2017**	EPI_ISL_287276	A/Togo/264/2019	EPI_ISL_363905	A/Belgium/S0430/2017	EPI_ISL_415331
**A/Wisconsin/300/2017**	EPI_ISL_287279	A/Pennsylvania/869/2019	EPI_ISL_363910	A/Belgium/S0428/2017	EPI_ISL_415330
**A/Wisconsin/306/2017**	EPI_ISL_287285	A/Virginia/30/2019	EPI_ISL_363912	A/Belgium/G0076/2017	EPI_ISL_415222
**A/Wisconsin/309/2017**	EPI_ISL_287288	A/New Jersey/29/2019	EPI_ISL_363913	A/Belgium/S0513/2017	EPI_ISL_415351
**A/Michigan/144/2017**	EPI_ISL_287301	A/Tennessee/67/2019	EPI_ISL_363916	A/Belgium/S1361/2017	EPI_ISL_415447
**A/Michigan/165/2017**	EPI_ISL_287304	A/Guatemala/2906/2019	EPI_ISL_363943	A/Belgium/S0182/2017	EPI_ISL_415307
**A/Michigan/210/2017**	EPI_ISL_287317	A/Florida/62/2019	EPI_ISL_363952	A/Belgium/G0059/2017	EPI_ISL_415217
**A/Michigan/212/2017**	EPI_ISL_287318	A/Florida/64/2019	EPI_ISL_363954	A/Belgium/S1160/2017	EPI_ISL_415423
**A/Michigan/224/2017**	EPI_ISL_287321	A/Minnesota/39/2019	EPI_ISL_363959	A/Belgium/G0219/2017	EPI_ISL_415234
**A/Michigan/231/2017**	EPI_ISL_287323	A/Minnesota/41/2019	EPI_ISL_363961	A/Belgium/G0711/2016	EPI_ISL_415200
**A/Michigan/261/2017**	EPI_ISL_287327	A/Cote D'Ivoire/14/2019	EPI_ISL_363969	A/Belgium/S0154/2017	EPI_ISL_415306
**A/Michigan/68/2017**	EPI_ISL_287330	A/Cote D'Ivoire/51/2019	EPI_ISL_363970	A/Belgium/S0460/2017	EPI_ISL_415340
**A/Michigan/67/2017**	EPI_ISL_287331	A/Delaware/40/2019	EPI_ISL_363972	A/Belgium/S1330/2017	EPI_ISL_415444
**A/Michigan/69/2017**	EPI_ISL_287332	A/Niger/6659/2019	EPI_ISL_363978	A/Belgium/S0856/2017	EPI_ISL_415391
**A/Michigan/70/2017**	EPI_ISL_287333	A/Argentina/55/2019	EPI_ISL_363993	A/Belgium/S0036/2017	EPI_ISL_415294
**A/Michigan/82/2017**	EPI_ISL_287338	A/Delaware/38/2019	EPI_ISL_363997	A/Belgium/S1321/2017	EPI_ISL_415442
**A/Michigan/90/2017**	EPI_ISL_287349	A/Togo/436/2019	EPI_ISL_364003	A/Belgium/S1241/2017	EPI_ISL_415431
**A/Michigan/116/2017**	EPI_ISL_287354	A/Rhode Island/26/2019	EPI_ISL_364004	A/Belgium/G0416/2017	EPI_ISL_415286
**A/Michigan/102/2017**	EPI_ISL_287360	A/West Virginia/35/2019	EPI_ISL_364007	A/Belgium/S0884/2017	EPI_ISL_415396
**A/Michigan/113/2017**	EPI_ISL_287366	A/Togo/35/2019	EPI_ISL_364010	A/Belgium/S0882/2017	EPI_ISL_415395
**A/Michigan/126/2017**	EPI_ISL_287376	A/Massachusetts/31/2019	EPI_ISL_364013	A/Belgium/G0254/2017	EPI_ISL_415241
**A/Michigan/136/2017**	EPI_ISL_287387	A/Texas/346/2019	EPI_ISL_364014	A/Belgium/G0269/2017	EPI_ISL_415244
**A/Michigan/147/2017**	EPI_ISL_287390	A/Irkutsk/RII-3884S/2019	EPI_ISL_364321	A/Belgium/S0939/2017	EPI_ISL_415407
**A/Michigan/156/2017**	EPI_ISL_287397	A/Koygorodok/RII-4264S/2019	EPI_ISL_364323	A/Belgium/S1095/2017	EPI_ISL_415419
**A/Michigan/161/2017**	EPI_ISL_287400	A/Krasnoyarsk/RII-3642S/2019	EPI_ISL_364324	A/Belgium/S0891/2017	EPI_ISL_415397

**A/Michigan/158/2017**	EPI_ISL_287402	A/Noyabrsk/RII-4711S/2019	EPI_ISL_364336	A/Belgium/S1187/2017	EPI_ISL_415427
**A/Texas/141/2017**	EPI_ISL_287424	A/Noyabrsk/RII-4712S/2019	EPI_ISL_364337	A/Belgium/S1010/2017	EPI_ISL_415412
**A/Texas/217/2017**	EPI_ISL_287438	A/Noyabrsk/RII-4717S/2019	EPI_ISL_364338	A/Belgium/S0021/2017	EPI_ISL_415292
**A/Texas/170/2017**	EPI_ISL_287439	A/Saint-Petersburg/RII-4034S/2019	EPI_ISL_364346	A/Belgium/S0069/2017	EPI_ISL_415296
**A/Texas/179/2017**	EPI_ISL_287443	A/Saint-Petersburg/RII-4056S/2019	EPI_ISL_364347	A/Belgium/S0107/2017	EPI_ISL_415300
**A/Texas/162/2017**	EPI_ISL_287445	A/Saint-Petersburg/RII-4112S/2019	EPI_ISL_364348	A/Belgium/S0275/2017	EPI_ISL_415312
**A/Texas/177/2017**	EPI_ISL_287461	A/Saint-Petersburg/RII-4529S/2019	EPI_ISL_364355	A/Belgium/G0244/2017	EPI_ISL_415239
**A/Texas/245/2017**	EPI_ISL_287464	A/Saint-Petersburg/RII-4545S/2019	EPI_ISL_364357	A/Belgium/G0007/2017	EPI_ISL_415204
**A/Texas/229/2017**	EPI_ISL_287491	A/Smolensk/RII-4187S/2019	EPI_ISL_364364	A/Belgium/S0921/2017	EPI_ISL_415404
**A/Texas/116/2017**	EPI_ISL_287494	A/Minnesota/44/2019	EPI_ISL_365746	A/Belgium/S0914/2017	EPI_ISL_415402
**A/Texas/195/2017**	EPI_ISL_287495	A/Guatemala/06/2019	EPI_ISL_365749	A/Belgium/S0875/2017	EPI_ISL_415394
**A/Texas/211/2017**	EPI_ISL_287507	A/Guatemala/27/2019	EPI_ISL_365756	A/Belgium/S0383/2017	EPI_ISL_415322
**A/Texas/213/2017**	EPI_ISL_287516	A/Mozambique/279/2019	EPI_ISL_365770	A/Belgium/S0466/2017	EPI_ISL_415341
**A/Texas/226/2017**	EPI_ISL_287517	A/Mozambique/301/2019	EPI_ISL_365772	A/Belgium/S1195/2017	EPI_ISL_415429
**A/Texas/117/2017**	EPI_ISL_287523	A/Kazakhstan/6/2019	EPI_ISL_365779	A/Belgium/S0777/2017	EPI_ISL_415381
**A/Texas/231/2017**	EPI_ISL_287538	A/Mozambique/206/2019	EPI_ISL_365780	A/Belgium/G0237/2017	EPI_ISL_415268
**A/Texas/249/2017**	EPI_ISL_287540	A/Cote D'Ivoire/527/2019	EPI_ISL_365783	A/Belgium/G0720/2016	EPI_ISL_415202
**A/Texas/255/2017**	EPI_ISL_287543	A/Cote D'Ivoire/650/2019	EPI_ISL_365785	A/Belgium/G0712/2016	EPI_ISL_415201
**A/Texas/264/2017**	EPI_ISL_287544	A/Hawaii/46/2019	EPI_ISL_365824	A/Belgium/G0246/2017	EPI_ISL_415240
**A/Texas/273/2017**	EPI_ISL_287547	A/Arizona/29/2019	EPI_ISL_365825	A/Belgium/S0386/2017	EPI_ISL_415324
**A/Tennessee/53/2017**	EPI_ISL_287553	A/Arizona/28/2019	EPI_ISL_365826	A/Belgium/S0470/2017	EPI_ISL_415342
**A/Tennessee/42/2017**	EPI_ISL_287555	A/Michigan/384/2019	EPI_ISL_365851	A/Belgium/G0294/2017	EPI_ISL_415247
**A/Tennessee/45/2017**	EPI_ISL_287558	A/Cote D'Ivoire/202/2019	EPI_ISL_365974	A/Belgium/G0341/2017	EPI_ISL_415258
**A/Tennessee/49/2017**	EPI_ISL_287559	A/Jamaica/0001/2019	EPI_ISL_366000	A/Belgium/S1247/2017	EPI_ISL_415433
**A/Tennessee/50/2017**	EPI_ISL_287560	A/Bolivia/563/2019	EPI_ISL_366002	A/Belgium/G0438/2017	EPI_ISL_415273
**A/Connecticut/33/2017**	EPI_ISL_287580	A/Togo/199/2019	EPI_ISL_366003	A/Belgium/S0836/2017	EPI_ISL_415389
**A/California/120/2017**	EPI_ISL_287606	A/Togo/189/2019	EPI_ISL_366005	A/Belgium/S0942/2017	EPI_ISL_415408
**A/Idaho/35/2017**	EPI_ISL_287617	A/Mozambique/300/2019	EPI_ISL_366006	A/Belgium/S0503/2017	EPI_ISL_415348
**A/Mozambique/309/2017**	EPI_ISL_287619	A/Texas/8689/2019	EPI_ISL_366131	A/Belgium/S0535/2017	EPI_ISL_415354
**A/Singapore/KK1211/2017**	EPI_ISL_288086	A/Virginia/8705/2019	EPI_ISL_366139	A/Belgium/S1430/2017	EPI_ISL_415450
**A/Singapore/GP2062/2017**	EPI_ISL_288091	A/Colorado/8578/2019	EPI_ISL_366464	A/Belgium/S1091/2017	EPI_ISL_415418
**A/Singapore/TT1517/2017**	EPI_ISL_288099	A/England/8581/2019	EPI_ISL_366466	A/Belgium/S0378/2017	EPI_ISL_415321
**A/Michigan/172/2017**	EPI_ISL_288277	A/Japan/8604/2019	EPI_ISL_366479	A/Belgium/S0695/2017	EPI_ISL_415368
**A/Michigan/178/2017**	EPI_ISL_288279	A/Maryland/8605/2019	EPI_ISL_366480	A/Belgium/S0779/2017	EPI_ISL_415382
**A/Michigan/179/2017**	EPI_ISL_288283	A/Montana/8613/2019	EPI_ISL_366487	A/Belgium/G0488/2017	EPI_ISL_415290
**A/Texas/303/2017**	EPI_ISL_288460	A/New York/8626/2019	EPI_ISL_366493	A/Belgium/S0424/2017	EPI_ISL_415329
**A/South Dakota/36/2017**	EPI_ISL_288469	A/New York/8627/2019	EPI_ISL_366494	A/Belgium/G0442/2017	EPI_ISL_415275
**A/Florida/96/2017**	EPI_ISL_288497	A/Ohio/8634/2019	EPI_ISL_366497	A/Belgium/G0336/2017	EPI_ISL_415256
**A/Ethiopia/1877/2017**	EPI_ISL_288511	A/Oklahoma/8641/2019	EPI_ISL_366503	A/Belgium/S0552/2017	EPI_ISL_415358
**A/Ethiopia/1817/2017**	EPI_ISL_288515	A/South Korea/8668/2019	EPI_ISL_366520	A/Belgium/G0317/2017	EPI_ISL_415253
**A/Ethiopia/2457/2017**	EPI_ISL_288517	A/South Korea/8674/2019	EPI_ISL_366523	A/Belgium/S0724/2017	EPI_ISL_415371
**A/Ethiopia/2438/2017**	EPI_ISL_288519	A/Illinois/8757/2019	EPI_ISL_366528	A/Belgium/S0725/2017	EPI_ISL_415372
**A/Idaho/38/2017**	EPI_ISL_288524	A/Italy/8763/2019	EPI_ISL_366533	A/Belgium/G0386/2017	EPI_ISL_415264
**A/California/128/2017**	EPI_ISL_288539	A/Japan/8769/2019	EPI_ISL_366539	A/Belgium/S0866/2017	EPI_ISL_415392
**A/Texas/306/2017**	EPI_ISL_288674	A/Montana/8781/2019	EPI_ISL_366548	A/Belgium/S1152/2017	EPI_ISL_415422
**A/Pennsylvania/98/2017**	EPI_ISL_289197	A/Nebraska/8782/2019	EPI_ISL_366549	A/Belgium/S1357/2017	EPI_ISL_415446
**A/Pennsylvania/166/2017**	EPI_ISL_289202	A/Nevada/8784/2019	EPI_ISL_366551	A/Belgium/G0463/2017	EPI_ISL_415282
**A/Pennsylvania/104/2017**	EPI_ISL_289211	A/New Mexico/8791/2019	EPI_ISL_366556	A/Belgium/S1468/2017	EPI_ISL_415451
**A/Pennsylvania/231/2017**	EPI_ISL_289224	A/New York/8797/2019	EPI_ISL_366561	A/Belgium/S0794/2017	EPI_ISL_415385
**A/Pennsylvania/60/2017**	EPI_ISL_289227	A/North Carolina/8798/2019	EPI_ISL_366562	A/Belgium/S0525/2017	EPI_ISL_415353
**A/Pennsylvania/219/2017**	EPI_ISL_289235	A/Ohio/8804/2019	EPI_ISL_366568	A/Belgium/S0332/2017	EPI_ISL_415318
**A/Pennsylvania/229/2017**	EPI_ISL_289236	A/Ohio/8808/2019	EPI_ISL_366570	A/Belgium/G0218/2017	EPI_ISL_415233
**A/Pennsylvania/169/2017**	EPI_ISL_289248	A/Ohio/8809/2019	EPI_ISL_366571	A/Belgium/G0482/2017	EPI_ISL_415288
**A/Pennsylvania/186/2017**	EPI_ISL_289249	A/Ohio/8810/2019	EPI_ISL_366572	A/Belgium/S1288/2017	EPI_ISL_415439
**A/Pennsylvania/246/2017**	EPI_ISL_289258	A/South Carolina/8815/2019	EPI_ISL_366574	A/Belgium/S1075/2017	EPI_ISL_415417
**A/Pennsylvania/144/2017**	EPI_ISL_289275	A/South Carolina/8817/2019	EPI_ISL_366576	A/Belgium/S1185/2017	EPI_ISL_415426
**A/Pennsylvania/193/2017**	EPI_ISL_289278	A/South Dakota/8821/2019	EPI_ISL_366580	A/Belgium/S1176/2017	EPI_ISL_415425
**A/Pennsylvania/233/2017**	EPI_ISL_289283	A/South Korea/8823/2019	EPI_ISL_366582	A/Belgium/G0455/2017	EPI_ISL_415279
**A/Pennsylvania/243/2017**	EPI_ISL_289284	A/South Korea/8832/2019	EPI_ISL_366591	A/Belgium/G0072/2017	EPI_ISL_415221
**A/Pennsylvania/34/2017**	EPI_ISL_289291	A/Spain/8834/2019	EPI_ISL_366593	A/Belgium/G0124/2017	EPI_ISL_415227
**A/Pennsylvania/35/2017**	EPI_ISL_289293	A/Texas/8839/2019	EPI_ISL_366598	A/Belgium/G0125/2017	EPI_ISL_415228
**A/Pennsylvania/212/2017**	EPI_ISL_289305	A/Utah/8841/2019	EPI_ISL_366600	A/Belgium/G0428/2017	EPI_ISL_415269
**A/Pennsylvania/121/2016**	EPI_ISL_289306	A/Virginia/8844/2019	EPI_ISL_366602	A/Belgium/G0443/2017	EPI_ISL_415276
**A/Pennsylvania/66/2017**	EPI_ISL_289319	A/Arizona/8871/2019	EPI_ISL_366721	A/Belgium/S0492/2017	EPI_ISL_415345
**A/Pennsylvania/83/2017**	EPI_ISL_289322	A/Arizona/8720/2019	EPI_ISL_366923	A/Belgium/S0754/2017	EPI_ISL_415376
**A/Pennsylvania/247/2017**	EPI_ISL_289330	A/Arizona/8721/2019	EPI_ISL_366924	A/Belgium/S0931/2017	EPI_ISL_415405
**A/Pennsylvania/160/2017**	EPI_ISL_289336	A/Belgium/8725/2019	EPI_ISL_366928	A/Belgium/S0507/2017	EPI_ISL_415349
**A/Pennsylvania/175/2017**	EPI_ISL_289338	A/Florida/8747/2019	EPI_ISL_366949	A/Belgium/S0800/2017	EPI_ISL_415386
**A/Pennsylvania/241/2017**	EPI_ISL_289344	A/Georgia/8748/2019	EPI_ISL_366950	A/Belgium/S0581/2017	EPI_ISL_415361
**A/Pennsylvania/185/2017**	EPI_ISL_289358	A/Germany/8753/2019	EPI_ISL_366952	A/Belgium/S0421/2017	EPI_ISL_415327
**A/Pennsylvania/206/2017**	EPI_ISL_289362	A/Texas/9009/2019	EPI_ISL_366996	A/Belgium/S0391/2017	EPI_ISL_415325
**A/Pennsylvania/242/2017**	EPI_ISL_289363	A/Concepcion/50492/2019	EPI_ISL_367543	A/Belgium/S1055/2017	EPI_ISL_415415
**A/Pennsylvania/106/2017**	EPI_ISL_289371	A/Iquique/10651/2019	EPI_ISL_367546	A/Belgium/G0408/2017	EPI_ISL_415285
**A/Pennsylvania/239/2017**	EPI_ISL_289372	A/Iquique/30817/2019	EPI_ISL_367547	A/Belgium/S0131/2017	EPI_ISL_415302
**A/Pennsylvania/140/2017**	EPI_ISL_289375	A/Martinique/346/2019	EPI_ISL_368163	A/Belgium/S0195/2017	EPI_ISL_415310
**A/Pennsylvania/159/2017**	EPI_ISL_289391	A/Martinique/571/2019	EPI_ISL_368177		

Table S3: Detailed list of reassortments detected by both manual inspection and computational methods

ID	Manual ID (0.95)	GiRaF (0.95)	Combination (0.95)	ID	Manual ID (0.95)	GiRaF (0.95)	Combination (0.95)
16-IG-0682	0	0	0	17-IS-00413	1	0	0
16-IG-0711	0	0	0	17-IS-00421	0	0	0
16-IG-0712	0	0	0	17-IS-00422	1	0	0
16-IG-0720	0	0	0	17-IS-00424	0	0	0
17-IG-00003	1	0	0	17-IS-00428	0	0	0
17-IG-00007	1	1	1	17-IS-00430	0	0	0
17-IG-00020	0	0	0	17-IS-00443	1	1	1
17-IG-00023	1	1	1	17-IS-00447	1	1	1
17-IG-00027	0	0	0	17-IS-00451	1	1	1
17-IG-00033	0	0	0	17-IS-00453	0	0	0
17-IG-00037	0	0	0	17-IS-00455	1	1	1
17-IG-00040	0	0	0	17-IS-00456	1	1	1
17-IG-00042	0	0	0	17-IS-00457	0	0	0
17-IG-00044	0	0	0	17-IS-00459	0	0	0
17-IG-00045	0	0	0	17-IS-00460	0	0	0
17-IG-00046	0	0	0	17-IS-00466	0	0	0
17-IG-00051	0	0	0	17-IS-00470	0	0	0
17-IG-00054	0	0	0	17-IS-00484	0	0	0
17-IG-00059	0	0	0	17-IS-00489	0	0	0
17-IG-00062	0	0	0	17-IS-00492	0	0	0
17-IG-00070	1	1	1	17-IS-00494	0	0	0
17-IG-00071	1	1	1	17-IS-00499	1	1	1
17-IG-00072	1	0	0	17-IS-00503	0	0	0
17-IG-00076	0	0	0	17-IS-00507	0	0	0
17-IG-00088	0	0	0	17-IS-00508	0	0	0
17-IG-00090	1	0	0	17-IS-00513	0	0	0
17-IG-00096	0	0	0	17-IS-00516	1	0	0
17-IG-00099	1	0	0	17-IS-00525	1	1	1
17-IG-00124	0	0	0	17-IS-00535	0	0	0
17-IG-00125	0	0	0	17-IS-00544	1	1	1
17-IG-00128	0	0	0	17-IS-00549	0	0	0
17-IG-00186	0	0	0	17-IS-00551	0	0	0
17-IG-00205	1	1	1	17-IS-00552	0	0	0
17-IG-00209	0	0	0	17-IS-00553	0	0	0
17-IG-00218	0	0	0	17-IS-00576	0	0	0
17-IG-00219	0	0	0	17-IS-00581	0	0	0
17-IG-00226	0	0	0	17-IS-00587	0	0	0

17-IG-00232	0	0	0	17-IS-00593	0	0	0
17-IG-00237	1	1	1	17-IS-00644	0	0	0
17-IG-00240	0	0	0	17-IS-00675	0	0	0
17-IG-00242	0	0	0	17-IS-00679	1	1	1
17-IG-00244	1	0	0	17-IS-00693	1	1	1
17-IG-00246	1	0	0	17-IS-00695	0	0	0
17-IG-00254	0	0	0	17-IS-00696	1	0	0
17-IG-00259	0	0	0	17-IS-00722	1	1	1
17-IG-00260	0	0	0	17-IS-00724	0	0	0
17-IG-00269	0	0	0	17-IS-00725	0	0	0
17-IG-00271	1	1	1	17-IS-00746	1	1	1
17-IG-00281	1	1	1	17-IS-00747	0	0	0
17-IG-00294	0	0	0	17-IS-00750	1	1	1
17-IG-00296	0	0	0	17-IS-00754	0	0	0
17-IG-00297	0	0	0	17-IS-00758	0	0	0
17-IG-00300	0	0	0	17-IS-00761	0	0	0
17-IG-00303	0	0	0	17-IS-00772	0	0	0
17-IG-00307	0	0	0	17-IS-00773	0	0	0
17-IG-00311	1	1	1	17-IS-00777	0	0	0
17-IG-00317	0	0	0	17-IS-00779	0	0	0
17-IG-00323	0	0	0	17-IS-00785	0	0	0
17-IG-00324	1	0	0	17-IS-00789	0	0	0
17-IG-00326	0	0	0	17-IS-00794	0	0	0
17-IG-00336	0	0	0	17-IS-00800	0	0	0
17-IG-00340	1	0	0	17-IS-00803	0	0	0
17-IG-00341	0	0	0	17-IS-00805	0	0	0
17-IG-00354	0	0	0	17-IS-00836	0	0	0
17-IG-00369	0	0	0	17-IS-00849	0	0	0
17-IG-00373	0	0	0	17-IS-00856	0	0	0
17-IG-00379	0	0	0	17-IS-00866	0	0	0
17-IG-00383	0	0	0	17-IS-00872	1	1	1
17-IG-00386	0	0	0	17-IS-00875	1	1	1
17-IG-00408	0	0	0	17-IS-00882	0	0	0
17-IG-00409	1	0	0	17-IS-00884	0	0	0
17-IG-00412	0	0	0	17-IS-00891	0	0	0
17-IG-00416	0	0	0	17-IS-00892	1	1	1
17-IG-00427	1	0	0	17-IS-00896	0	0	0
17-IG-00428	0	0	0	17-IS-00897	1	1	1
17-IG-00433	0	0	0	17-IS-00910	0	0	0

17-IG-00434	0	0	0	17-IS-00914	0	0	0
17-IG-00437	0	0	0	17-IS-00917	0	0	0
17-IG-00438	0	0	0	17-IS-00921	0	0	0
17-IG-00441	0	0	0	17-IS-00931	0	0	0
17-IG-00442	0	0	0	17-IS-00938	1	0	0
17-IG-00443	0	0	0	17-IS-00939	0	0	0
17-IG-00447	1	0	0	17-IS-00942	0	0	0
17-IG-00453	1	1	1	17-IS-00960	0	0	0
17-IG-00455	0	0	0	17-IS-00978	0	0	0
17-IG-00457	0	0	0	17-IS-01006	0	0	0
17-IG-00462	1	0	0	17-IS-01010	0	0	0
17-IG-00463	0	0	0	17-IS-01029	0	1	0
17-IG-00470	0	0	0	17-IS-01052	0	0	0
17-IG-00482	0	0	0	17-IS-01055	0	0	0
17-IG-00486	1	0	0	17-IS-01068	1	1	1
17-IG-00488	0	0	0	17-IS-01075	0	0	0
17-IG-00513	1	0	0	17-IS-01091	0	0	0
17-IS-00021	0	0	0	17-IS-01095	0	0	0
17-IS-00028	0	0	0	17-IS-01104	0	0	0
17-IS-00036	0	0	0	17-IS-01118	0	0	0
17-IS-00058	0	0	0	17-IS-01152	0	0	0
17-IS-00069	0	0	0	17-IS-01160	0	0	0
17-IS-00078	1	1	1	17-IS-01166	0	0	0
17-IS-00093	0	0	0	17-IS-01176	0	0	0
17-IS-00098	0	0	0	17-IS-01185	0	0	0
17-IS-00107	0	0	0	17-IS-01187	0	0	0
17-IS-00113	1	1	1	17-IS-01191	0	0	0
17-IS-00131	0	0	0	17-IS-01195	0	0	0
17-IS-00138	0	0	0	17-IS-01199	1	1	1
17-IS-00146	0	0	0	17-IS-01241	0	0	0
17-IS-00150	1	1	1	17-IS-01246	0	0	0
17-IS-00154	0	0	0	17-IS-01247	1	1	1
17-IS-00182	0	0	0	17-IS-01252	1	1	1
17-IS-00188	0	0	0	17-IS-01264	0	0	0
17-IS-00194	0	0	0	17-IS-01266	0	0	0
17-IS-00195	0	0	0	17-IS-01268	0	0	0
17-IS-00268	0	0	0	17-IS-01288	0	0	0
17-IS-00275	0	0	0	17-IS-01306	0	0	0
17-IS-00281	0	0	0	17-IS-01314	0	0	0

17-IS-00282	1	1	1	17-IS-01321	0	0	0
17-IS-00287	0	0	0	17-IS-01329	1	1	1
17-IS-00303	0	0	0	17-IS-01330	0	0	0
17-IS-00322	1	1	1	17-IS-01337	0	0	0
17-IS-00332	0	0	0	17-IS-01357	1	1	1
17-IS-00354	0	0	0	17-IS-01361	0	0	0
17-IS-00365	0	0	0	17-IS-01383	0	0	0
17-IS-00378	0	0	0	17-IS-01413	0	0	0
17-IS-00383	0	0	0	17-IS-01430	0	0	0
17-IS-00385	0	0	0	17-IS-01468	0	0	0
17-IS-00386	0	0	0	17-IS-01475	0	0	0
17-IS-00391	0	0	0				

Table S4: Statically significant results using the Fisher's exact test with FDR correction for association between host characteristics and sample parameters with the newly-defined phylogenetic groups for the whole-genome and all the individual segments.

The number of samples (n) are indicated for each group. For the HA segment, the number of samples belonging to the same group identified for the whole-genome is indicated (WG ~ %). The magnitude of the significant association is defined by the effect size (ES) and its confidence interval (CI). Confounders of significant associations are also indicated. Results for other segments are presented in Supplementary File S6.

Groups	Whole Genome (WG)	PB2	PB1	PA	HA	NP	NA	MP	NS
Group 3C 2a	n=4	n = 4<br>WG ~ 100%	n = 4<br>WG ~ 100%	n = 4<br>WG ~ 100%	n = 4<br>WG ~ 100%	n = 4<br>WG ~ 100%	n = 4<br>WG ~ 100%	n = 4<br>WG ~ 100%	n = 9<br>WG ~ 44.4%
Group 3C 2a 1	n=26	n = 22<br>WG ~ 45.5%	n = 26<br>WG ~ 73.1%	n = 30<br>WG ~ 80%	n = 36<br>WG ~ 52.8%	n = 22<br>WG ~ 95.5%	n = 22<br>WG ~ 95.5%	n = 28<br>WG ~ 64.3%	n = 12<br>WG ~ 100%
Group 3C 2a 1 (2)	n=62	n = 70<br>WG ~ 77.1%	n = 58<br>WG ~ 93.1%	n = 54<br>WG ~ 96.3%	n = 55<br>WG ~ 90.9%	n = 60<br>WG ~ 100%	n = 67<br>WG ~ 92.5%	n = 54<br>WG ~ 96.3%	n = 59<br>WG ~ 100%
Group 3C 2a 1a	n=25	n = 30<br>WG ~ 73.3%	n = 32<br>WG ~ 75%	n = 24<br>WG ~ 91.7%	n = 27<br>WG ~ 88.9%	n = 23<br>WG ~ 95.7%	n = 26<br>WG ~ 84.6%	n = 42<br>WG ~ 57.1%<br>**Significant results for diabetes:**<br>No diabetes >> Diabetes<br>ES [95% CI]: 2.70E+07 [2.40E-21-9.45E+160]<br>**Significant results for the Severity:**<br>Mild > Moderate > Severe<br>ES [95% CI] (Mild-Severe): 6.65 [1.83-42.85]<br>Confounders: Vaccination, antibiotics, diabetes, immuno-deficiency, age category	n = 24<br>WG ~ 87.5%
Group 3C 2a 1a (2)	n=37<br>**Significant results for the Sampling Period:**<br>Beginning ~ Peak > End<br>ES [95% CI] (Peak-End): 15.00 [3.06-271.34]<br>Confounders: Severity, vaccination, antibiotics, obesity, neurologic diseases, hepatic insufficiency, stay in the ICU	n = 32<br>WG ~ 93.8%<br>**Significant results for the Sampling Period:**<br>Beginning ~ Peak > End<br>ES [95% CI] (Peak-End): 11.43 [2.29-207.65]<br>Confounders: Severity, stay in the ICU	n = 31<br>WG ~ 100%<br>**Significant results for the Sampling Period:**<br>Beginning ~ Peak > End<br>ES [95% CI] (Peak-End): 11.43 [2.29-207.65]<br>Confounders: Severity, antibiotics	n = 43<br>WG ~ 81.4%	n = 31<br>WG ~ 80.6%	n = 40<br>WG ~ 92.5%	n = 26<br>WG ~ 100%<br>**Significant results for a stay in the ICU:**<br>No ICU < ICU<br>ES [95% CI]: 2.29 [0.50-7.86]<br>Confounders: Severity, antibiotics, respiratory diseases, cardiac diseases, diabetes, renal insufficiency	n = 19<br>WG ~ 100%	n = 19<br>WG ~ 94.7%
Group 3C 2a 1b	n=20	n = 19<br>*WG – 100%*	n = 10<br>*WG – 100%*	n = 19<br>*WG – 100%*	n = 19<br>*WG – 100%*	n = 19<br>*WG – 100%*	n = 9<br>*WG – 100%*	n = 23<br>*WG – 82.6%*	n = 27<br>*WG – 70.4%*
Group 3C 2a 2	*n=9*	*n = 8*<br>WG ~ 100%	*n = 7*<br>WG ~ 100%	*n = 9*<br>WG ~ 100%	*n = 8*<br>WG ~ 87.5%	*n = 7*<br>WG ~ 100%	*n = 8*<br>WG ~ 100%	*n = 9*<br>WG ~ 100%	*n = 9*<br>WG ~ 88.9%
Group 3C 2a 3	n=59<br>**Significant results for the Sex:**<br>Male > Female<br>ES [95% CI]: 2.80 [1.51-5.34]<br>Confounders: Vaccination, antibiotics, sampling period<br>**Significant results for the Sampling Period:**<br>Beginning < Peak ~ End<br>ES [95% CI] (Beginning-Peak): 2.99 [1.31-7.76]<br>Confounders: Severity, vaccination, antibiotics, obesity, neurologic diseases, hepatic insufficiency, stay in the ICU	n = 54<br>WG ~ 100%<br>**Significant results for the Sex:**<br>Male > Female<br>ES [95% CI]: 2.57 [1.37-4.98]<br>Confounders: Vaccination, antibiotics, sampling period	n = 55<br>WG ~ 98.2%<br>**Significant results for the Sex:**<br>Male > Female<br>ES [95% CI]: 2.57 [1.37-4.98]<br>Confounders: Vaccination, antibiotics, sampling period<br>**Significant results for the Sampling Period:**<br>Beginning < Peak ~ End<br>ES [95% CI] (Beginning-Peak): 3.83 [1.52-11.70]<br>Confounders: Vaccination, Antibiotics	n = 53<br>WG ~ 100%	n = 55<br>WG ~ 100%<br>**Significant results for the Sex:**<br>Male > Female<br>ES [95% CI]: 2.67 [1.42-5.18]<br>Confounders: Vaccination, antibiotics, sampling period	n = 59<br>WG ~ 100%<br>**Significant results for the Sex:**<br>Male > Female<br>ES [95% CI]: 2.80 [1.51-5.34]<br>Confounders: Vaccination, antibiotics, sampling period	n = 56<br>WG ~ 98.2%	n = 54<br>WG ~ 100%<br>**Significant results for the Sex:**<br>Male > Female<br>ES [95% CI]: 2.86 [1.51-5.63]<br>Confounders: Vaccination, antibiotics, sampling period	n = 52<br>WG ~ 100%<br>**Significant results for the Sex:**<br>Male > Female<br>ES [95% CI]: 2.65 [1.39-5.21]<br>Confounders: Vaccination, antibiotics, sampling period
Group 1	n=8<br>**Significant results for the Surveillance system:**<br>ILI > SARI<br>ES [95% CI]: 13.57 [2.36-255.93]<br>Confounders: Vaccination, antibiotics, cardiac diseases, renal insufficiency, neurological diseases	n = 10<br>WG ~ 50%<br>**Significant results for the Surveillance system:**<br>ILI > SARI<br>ES [95% CI]: 2.61E+08 [1.63E-76-NA]<br>**Significant results for the Age Category:**<br>0-15 << 15-59 > 60+<br>ES [95% CI] (<15 – 15-59): 4.31+07 [0.00-NA]<br>Confounders: Severity, sex, vaccination, alive/death, antibiotics		n = 10<br>WG ~ 60%	n = 11<br>WG ~ 72.7%<br>**Significant results for the Surveillance system:**<br>ILI > SARI<br>ES [95% CI]: 20.13 [3.76-372.65]<br>Confounders: Vaccination, antibiotics, cardiac diseases, renal insufficiency	n = 19<br>WG ~ 42.1%<br>**Significant results for the Surveillance system:**<br>ILI > SARI<br>ES [95% CI]: 32.23 [6.35-586.28]<br>Confounders: Vaccination, antibiotics, cardiac diseases, renal insufficiency	n = 19<br>WG ~ 42.1%<br>**Significant results for the Surveillance system:**<br>ILI > SARI<br>ES [95% CI]: 34.85 [6.91-634.96]<br>Confounders: Vaccination, cardiac diseases, renal insufficiency		n = 37<br>WG ~ 10.8%<br>**Significant results for the Surveillance system:**<br>ILI > SARI<br>ES [95% CI]: 2.99 [1.46-6.25]<br>Confounders: Vaccination, antibiotics, age category<br>**Significant results for the Age Category:**<br>0-15 < 15-59 >> 60+<br>ES [95% CI] (15-59 – 60+): 4.54 [2.00-10.55]<br>Confounders: Severity, vaccination, cardiac diseases, diabetes, sampling period

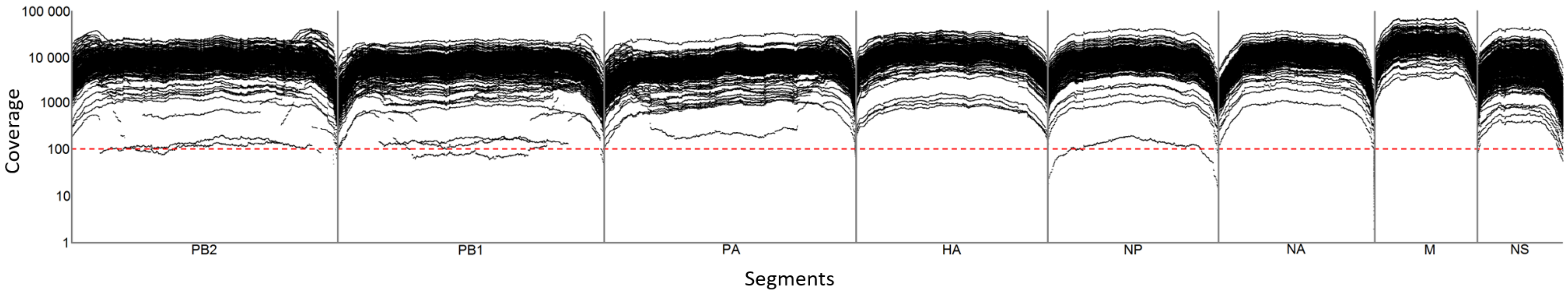

Figure S1: Coverage depth and breadth for the 253 sequenced Influenza A(H3N2) samples using WGS stratified per segment.
Coverage depth and gene segment position are presented on the y-axis and x-axis respectively. Every line represents one sample. The red dotted line represents a coverage depth of 100x.

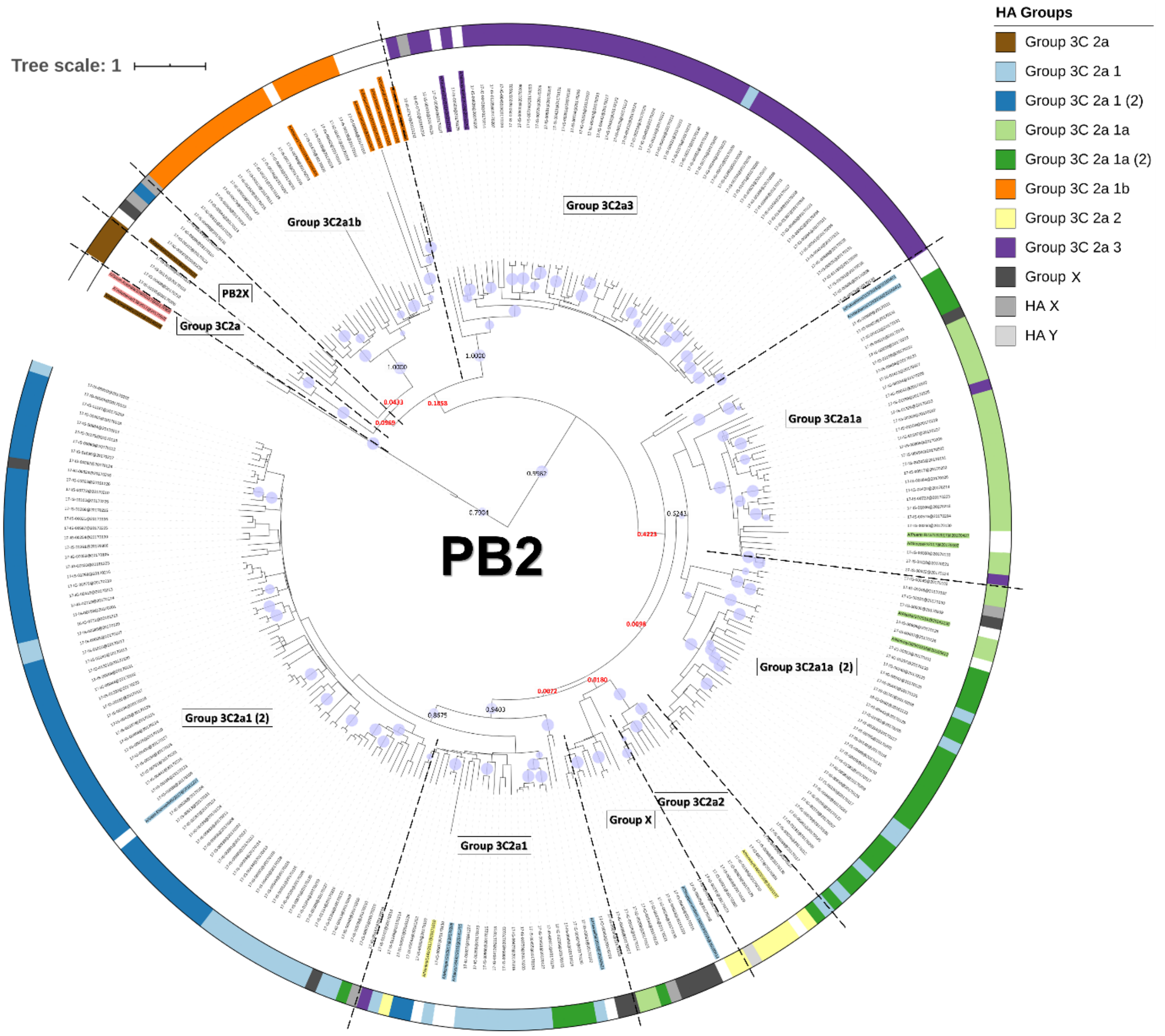

Figure S2: Phylogenetic tree based on the PB2 gene.

Colored names indicate additional references selected from the ECDC reports and the public instance of Nextstrain. The circular colored outer ring around the tree represents the assigned groups based on phylogenetic analysis of the HA segment in BEAST. “Group X” clustered together in a separate cluster from the other phylogenetic groups. Group “PB2X” contains samples that could not be classified. Posterior probability values are indicated on key nodes that separate groups, and colored in red if they are below 0.5. Additionally, the size of blue disks on nodes represent the posterior probability scaled between 0.5 and 1. The scale bar represents the average number of substitutions per site.

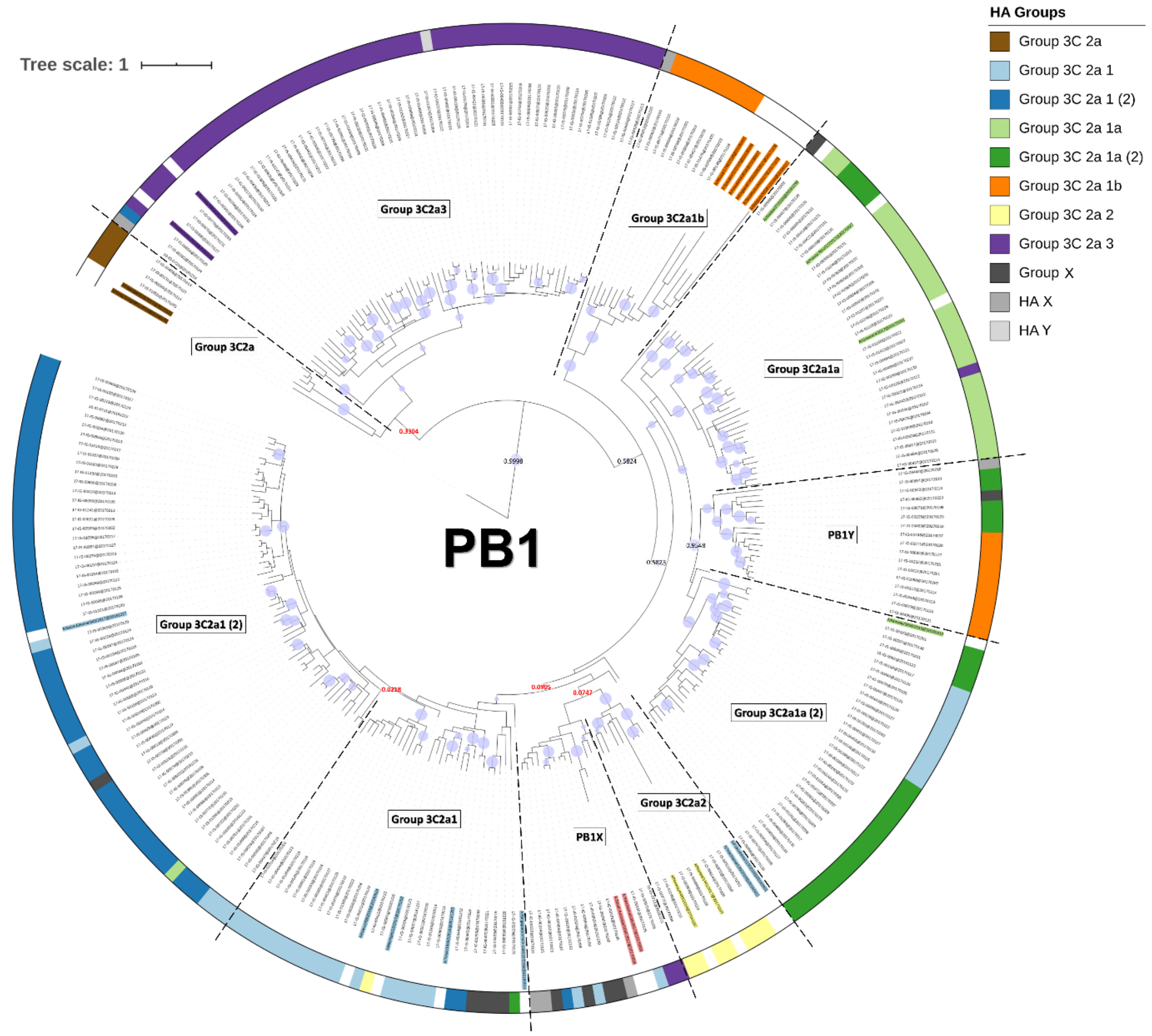

Figure S3: Phylogenetic tree based on the PB1 gene.
The colored names are the references coming from the ECDC reports and Nextstrain. The circular colored outer tree ring around the tree represents the assigned groups based on phylogenetic analysis of the HA segment in BEAST. Groups "PB1X" and "PB1Y" contain samples that could not be classified. Posterior probability values are indicated on the key nodes that separate groups, and colored in red if they are below 0.5. In addition, the size of the blue disks on the nodes represent the posterior probability between 0.5 and 1. The scale bar represents the average number of substitutions per site.

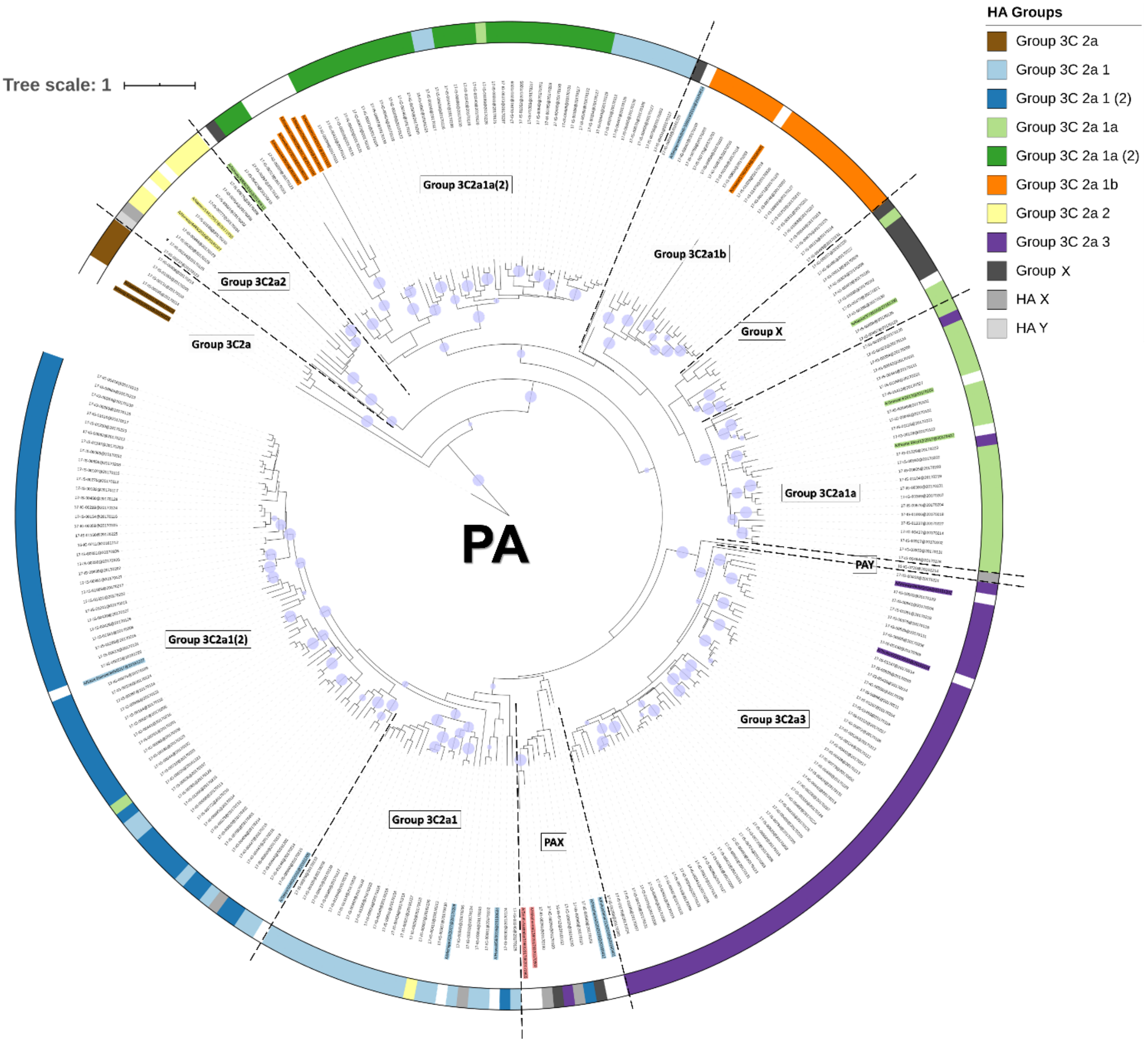

Figure S4: Phylogenetic tree based on the PA gene.
The colored names are the references coming from the ECDC reports and Nextstrain. The circular colored outer tree ring around the tree represents the assigned groups based on phylogenetic analysis of the HA segment in BEAST. "Group X" clustered together in a separate cluster from the other phylogenetic groups. Groups "PAX" and "PAY" contain samples that could not be classified. Posterior probability values are indicated on the key nodes that separate groups, and colored in red if they are below 0.5. In addition, the size of the blue disks on the nodes represent the posterior probability between 0.5 and 1. The scale bar represents the average number of substitutions per site.

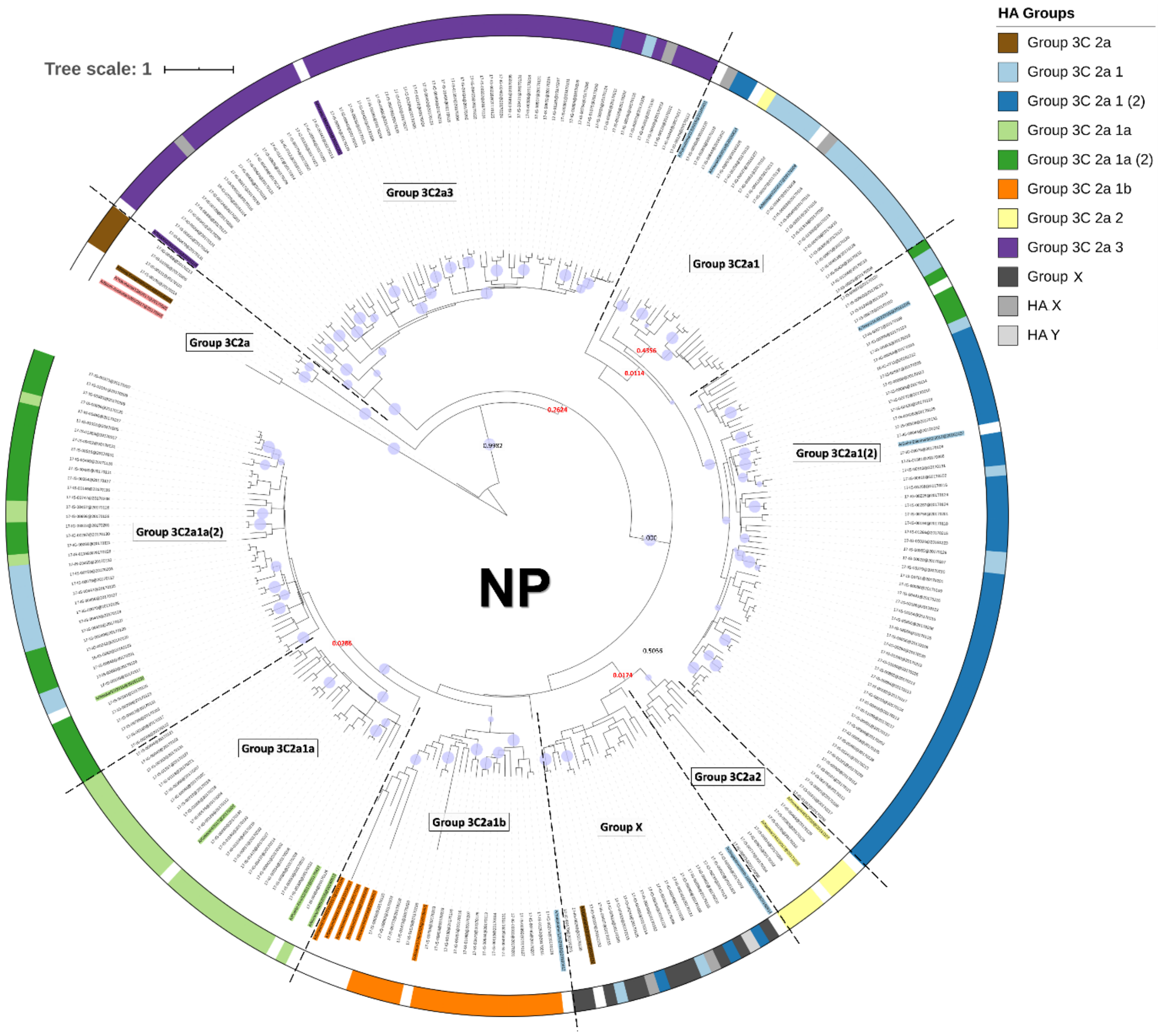

Figure S5: Phylogenetic tree based on the NP gene.
The colored names are the references coming from the ECDC reports and Nextstrain. The circular colored outer tree ring around the tree represents the assigned groups based on phylogenetic analysis of the HA segment in BEAST. “Group X” clustered together in a separate cluster from the other phylogenetic groups. Posterior probability values are indicated on the key nodes that separate groups, and colored in red if they are below 0.5. In addition, the size of the blue disks on the nodes represent the posterior probability between 0.5 and 1. The scale bar represents the average number of substitutions per site.

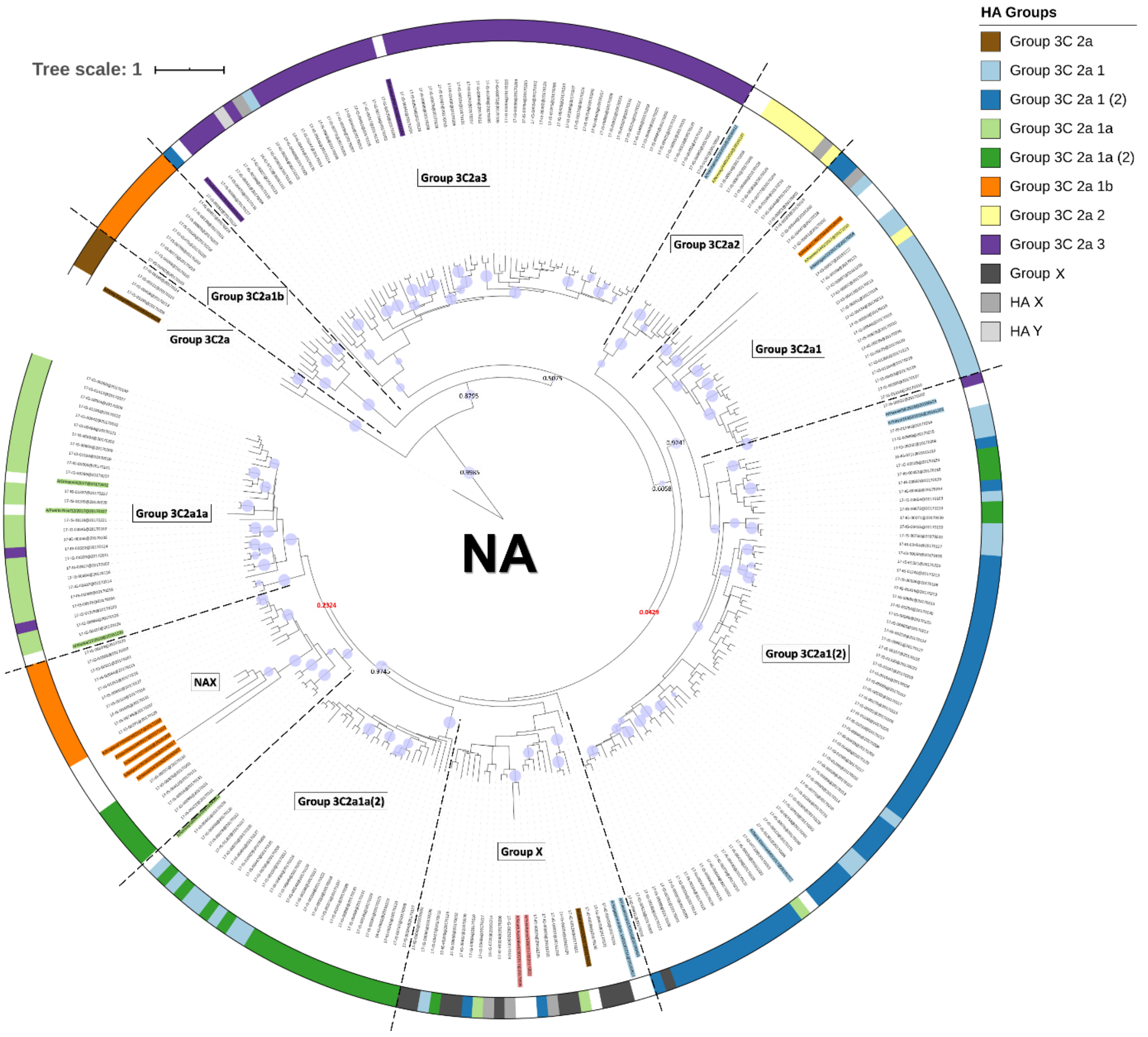

Figure S6: Phylogenetic tree based on the NA gene.

The colored names are the references coming from the ECDC reports and Nextstrain. The circular colored outer tree ring around the tree represents the assigned groups based on phylogenetic analysis of the HA segment in BEAST. "Group X" clustered together in a separate cluster from the other phylogenetic groups. Group "NAX" contains samples that could not be classified. Posterior probability values are indicated on the key nodes that separate groups, and colored in red if they are below 0.5. In addition, the size of the blue disks on the nodes represent the posterior probability between 0.5 and 1. The scale bar represents the average number of substitutions per site.

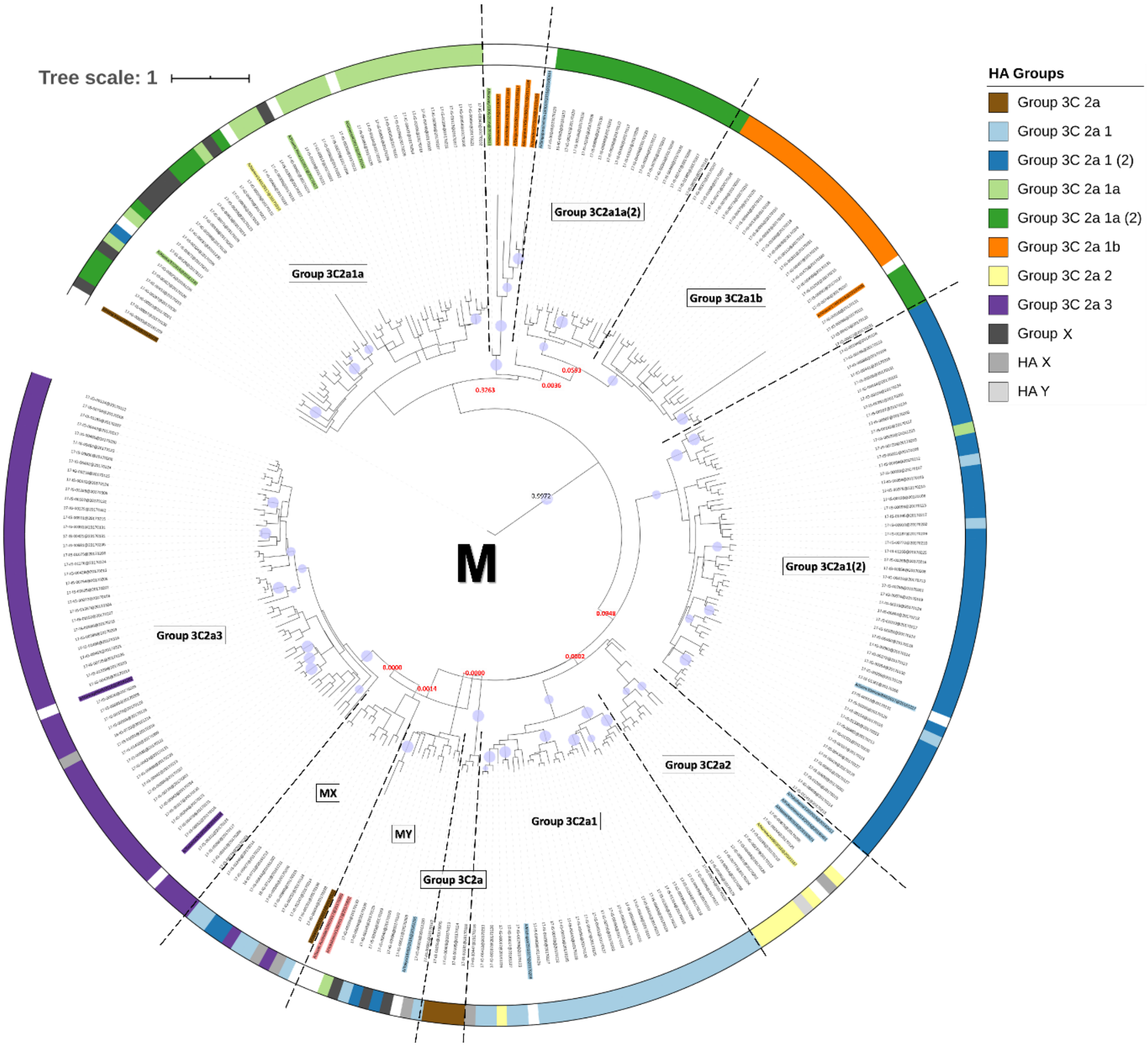

Figure S7: Phylogenetic tree based on the M gene.
The colored names are the references coming from the ECDC reports and Nextstrain. The circular colored outer tree ring around the tree represents the assigned groups based on phylogenetic analysis of the HA segment in BEAST. Groups “MX” and “MY” contain samples that could not be classified. Posterior probability values are indicated on the key nodes that separate groups, and colored in red if they are below 0.5. In addition, the size of the blue disks on the nodes represent the posterior probability between 0.5 and 1. The scale bar represents the average number of substitutions per site.

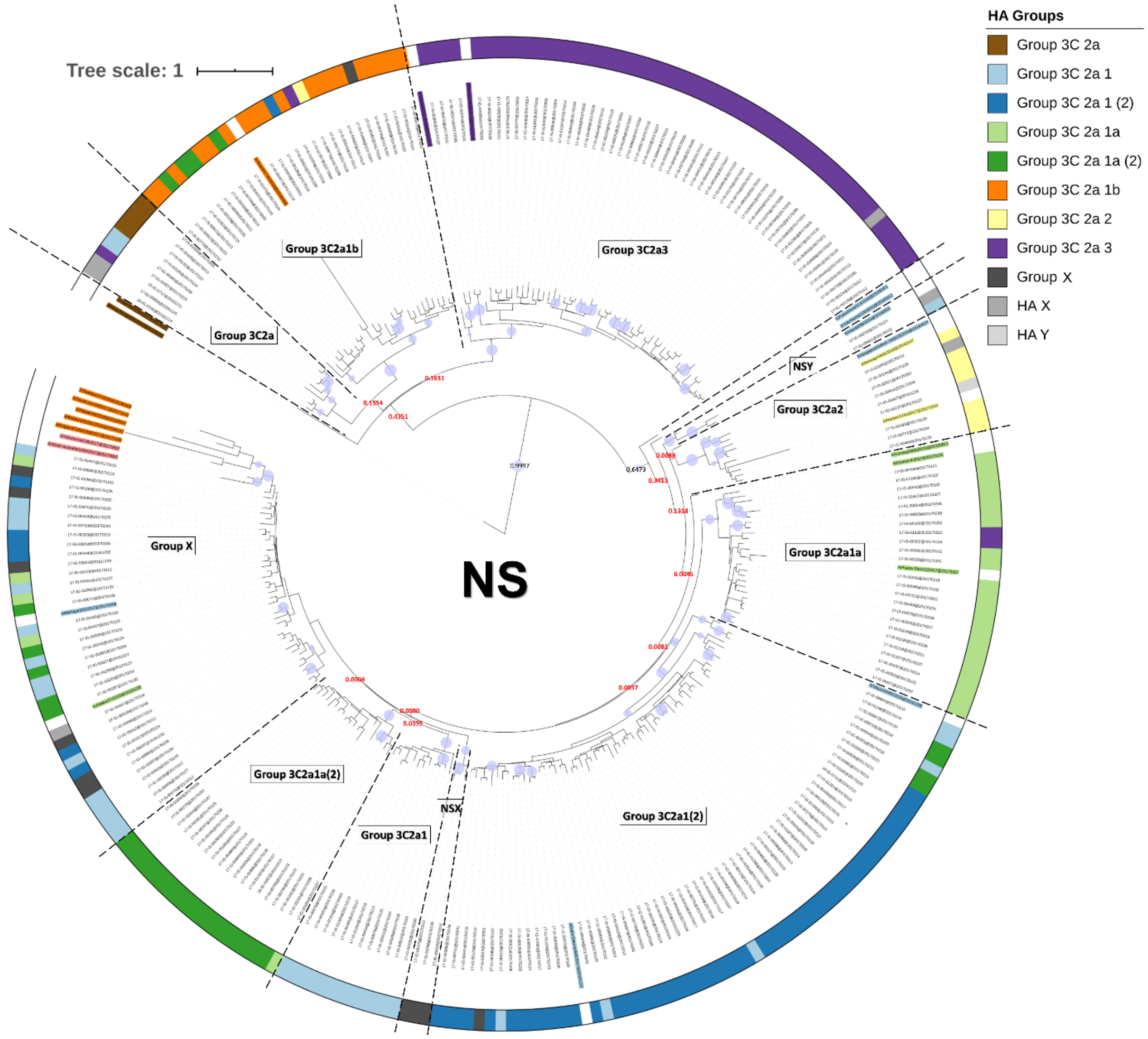

Figure S8: Phylogenetic tree based on the NS gene.

The colored names are the references coming from the ECDC reports and Nextstrain. The circular colored outer tree ring around the tree represents the assigned groups based on phylogenetic analysis of the HA segment in BEAST. "Group X" clustered together in a separate cluster from the other phylogenetic groups. Groups "NSX" and "NSY" contain samples that could not be classified. Posterior probability values are indicated on the key nodes that separate groups, and colored in red if they are below 0.5. In addition, the size of the blue disks on the nodes represent the posterior probability between 0.5 and 1. The scale bar represents the average number of substitutions per site.

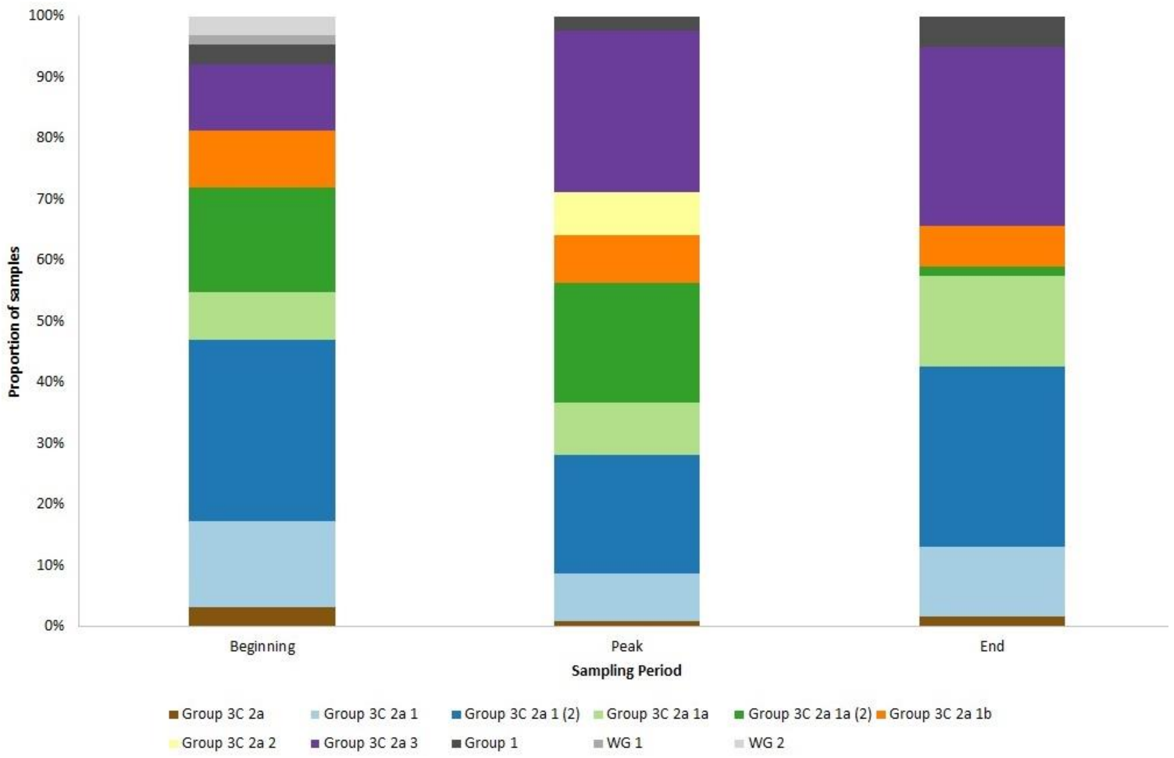

Figure S9: Proportion of phylogenetic groups based on the whole genome tree per sampling period.
